# Bona fide choline monoxygenases evolved in Amaranthaceae plants from oxygenases of unknown function: Evidence from phylogenetics, homology modeling and docking studies

**DOI:** 10.1371/journal.pone.0204711

**Published:** 2018-09-26

**Authors:** Javier Carrillo-Campos, Héctor Riveros-Rosas, Rogelio Rodríguez-Sotres, Rosario A. Muñoz-Clares

**Affiliations:** 1 Departamento de Bioquímica, Facultad de Química, Universidad Nacional Autónoma de México, Ciudad de México, México; 2 Departamento de Bioquímica, Facultad de Medicina, Universidad Nacional Autónoma de México, Ciudad de México, México; Universidade de Lisboa Instituto Superior de Agronomia, PORTUGAL

## Abstract

Few land plants can synthesize and accumulate the osmoprotectant glycine betaine (GB) even though this metabolic trait has major adaptive importance given the prevalence of drought, hypersaline soils or cold. GB is synthesized from choline in two reactions catalyzed by choline monooxygenases (CMOs) and enzymes of the family 10 of aldehyde dehydrogenases (ALDH10s) that gained betaine aldehyde dehydrogenase activity (BADH). Homolog genes encoding CMO and ALDH10 enzymes are present in all known land plant genomes, but since GB-non-accumulators plants lack the BADH-type ALDH10 isozyme, they would be expected to also lack the CMO activity to avoid accumulation of the toxic betaine aldehyde. To explore CMOs substrate specificity, we performed amino acid sequence alignments, phylogenetic analysis, homology modeling and docking simulations. We found that plant CMOs form a monophyletic subfamily within the Rieske/mononuclear non-heme oxygenases family with two clades: CMO1 and CMO2, the latter diverging from CMO1 after gene duplication. CMO1 enzymes are present in all plants; CMO2s only in the Amaranthaceae high-GB-accumulators plants. CMO2s, and particularly their mononuclear non-heme iron domain where the active site is located, evolved at a faster rate than CMO1s, which suggests positive selection. The homology model and docking simulations of the spinach CMO2 enzyme showed at the active site three aromatic residues forming a box with which the trimethylammonium group of choline could interact through cation-π interactions, and a glutamate, which also may interact with the trimethylammonium group through a charge-charge interaction. The aromatic box and the carboxylate have been shown to be critical for choline binding in other proteins. Interestingly, these residues are conserved in CMO2 proteins but not in CMO1 proteins, where two of these aromatic residues are leucine and the glutamate is asparagine. These findings reinforce our proposal that the CMO1s physiological substrate is not choline but a still unknown metabolite.

## Introduction

Land plants are sessile organisms that have evolved a great variety of strategies to escape from, or contend with, the many kinds of abiotic and biotic stresses to which they may be exposed during their lives. Osmotic stress—caused by drought, saline soils or low temperatures—constitutes the major limitation of agricultural production worldwide [1]. To cope with osmotic stress plants synthesize neutral, highly soluble, small organic compounds known as compatible solutes or osmoprotectants because they can be accumulated up to high concentrations without any toxic effect, thus preventing water loss and maintaining cell turgor in a hypertonic environment [2], as well as protecting the intracellular proteins from the noxious effects of abnormally high ion concentrations [3]. Glycine betaine (GB) is the most effective osmoprotectant [4] and those plants that synthesize it—known as GB-accumulators—tolerate osmotic stress much better than the non-accumulators [5]. In addition, given the observed health benefits of the intake of GB in humans [6] and animals [7], the ability of an edible plant to accumulate GB is important not only from an agricultural but also from a nutritional point of view. Because of these reasons and because many important crops and forage plants are GB-non-accumulators, the engineering of the ability to synthesize this osmoprotectant has been, and still is, a biotechnological goal [8]. However, although the synthesis of GB was increased in some transgenic plants, the levels of GB attained under osmotic stress conditions were well below those attained by the natural GB-accumulators (reviewed in [9]). This outcome emphasizes the need to get a deeper understanding of the enzymes involved in GB biosynthesis.

**Fig 1:**
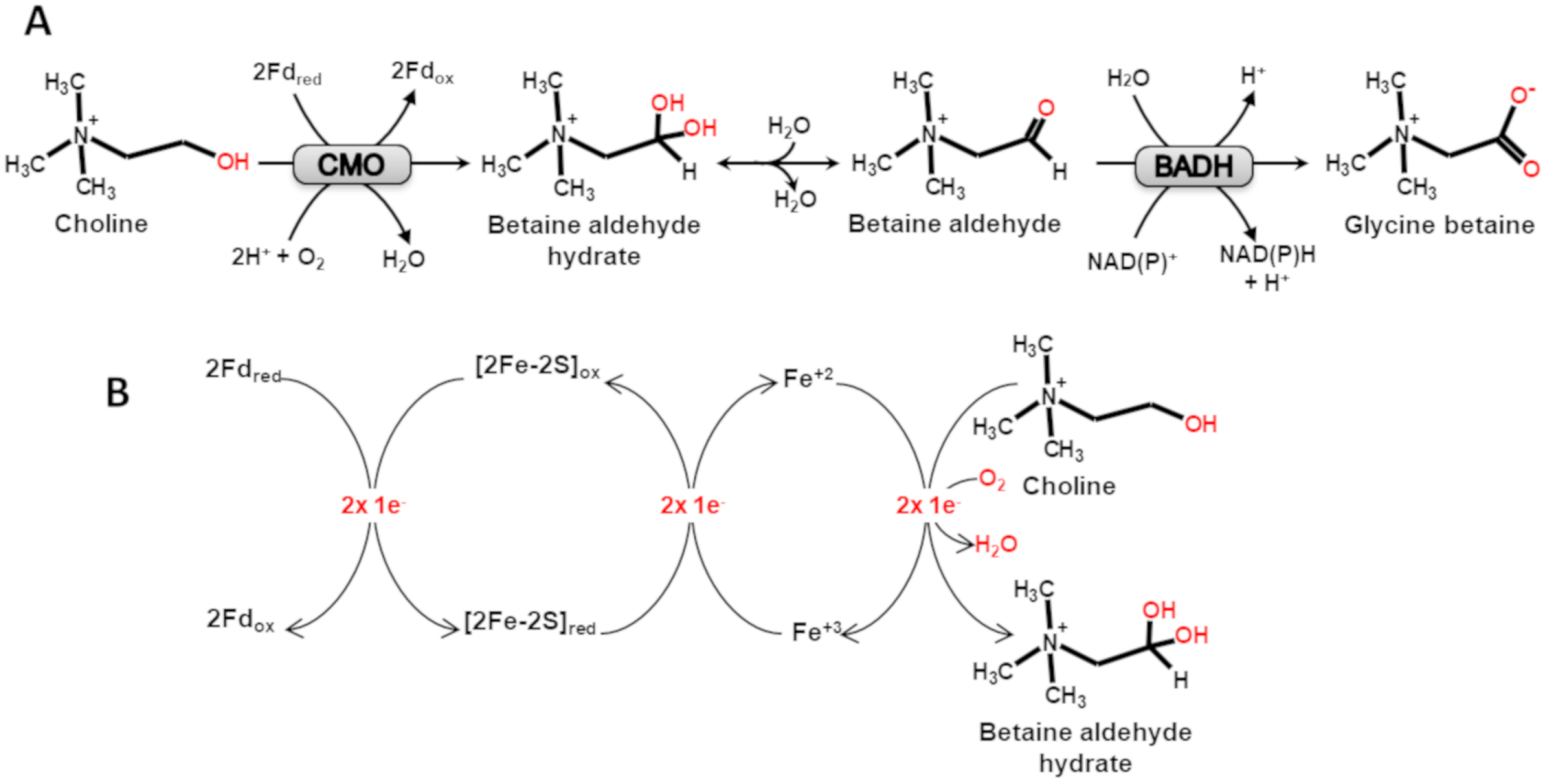
Biosynthesis of glycine betaine in plants. (A) Schematic representation of the two steps from choline. CMO, choline monooxygenase; BADH, betaine aldehyde dehydrogenase; Fd_red and_ Fd_ox_, reduced and oxidized ferredoxin, respectively (B) Schematic representation of the electron transfer pathway from Fd_red_ to choline in the CMO catalyzed reaction. [2Fe-2S]_red_ and [2Fe-2S]_ox_, reduced and oxidized state of the CMO Rieske center, respectively; Fe^2+^ and Fe^3+^, reduced and oxidized state, respectively, of the CMO mononuclear non-heme iron. The betaine aldehyde hydrate formed in this reaction is in equilibrium with the aldehyde form, which is the BADH substrate.

In land plants, GB is formed from choline in a short pathway consisting only of two steps (Fig 1A): formation of betaine aldehyde in a reaction catalyzed by choline monoxygenases (E.C.1.14.15.7; CMOs)—enzymes apparently found only in plants [10]—and the oxidation of betaine aldehyde to GB in a reaction catalyzed by betaine aldehyde dehydrogenases (E.C. 1.2.1.8; BADHs) [11]. Plant BADHs belong to the family 10 of the aldehyde dehydrogenase (ALDH) superfamily [12,13]. CMOs are monooxygenases that contains Rieske-type [2Fe–2S] and mononuclear non-heme iron centers [14]. They catalyze the irreversible hydroxylation of choline (Fig 1B), using molecular oxygen and the electrons provided by reduced ferredoxin (Fd_red_) to form betaine aldehyde hydrate, which in aqueous solution is in equilibrium with betaine aldehyde—the BADH substrate [15].

Gene duplication and a single change of a residue (Ala/Cys441 for Ile441, spinach BADH numbering) allowed the acquisition of the BADH activity by some ALDH10 isozymes [13,16]. The absence of the Ala/Cys441-type isozyme has been proposed to be a major limitation for the synthesis of GB in plants [13,16]. Given the high toxicity of betaine aldehyde [17], the CMO activity would be deleterious for the plant if it takes place without an accompanying BADH activity that converts betaine aldehyde to the innocuous GB. It would be then expected that CMO enzymes would be present only in GB-accumulator plants. However, the *CMO* gene has also been found in GB-non-accumulator plants [18,19], which raises the possibility that in these GB non-accumulators plants either: (i) their *CMO* genes are not expressed or expressed at a very low level; (ii) their CMO proteins are non-functional; or (iii) their CMO enzymes oxidize a substrate different from choline and, therefore, participate in a metabolic pathway different than the synthesis of GB. Abnormally processed *CMO* transcripts were found in rice [20], a GB-non-accumulator plant, and the oxidation of choline to betaine aldehyde by CMO proteins has been so far measured only in GB-accumulators plants that also have active BADH enzymes: *Spinacea oleracea* (spinach) [10,14], *Beta vulgaris* (sugar beet) [21], *Amaranthus caudatus* [21], and *Hordeum vulgare* (barley) [19]. Moreover, *Escherichia coli* cells transformed with the *CMO* gene from *Arabidopsis thaliana*, a GB-non-accumulator plant, expressed the CMO protein but were unable to produce GB when the cells were grown in the presence of choline, whereas the same *E*. *coli* cells transformed with a spinach *CMO* gene did produce GB [22]. These results were interpreted as a proof of *A*. *thaliana* CMO being non-functional, but these data are equally consistent with a different substrate specificity of this enzyme. In others words, the recombinant *A*. *thaliana* CMO protein may not use choline as substrate and, therefore, not be a functional choline monooxygenase, but still be a functional oxygenase acting on a different substrate.

In the present work we aimed at getting a deeper understanding of how the synthesis of GB from choline evolved in land plants by focusing on the study of the evolutive history of the CMO proteins. We carried out a comprehensive phylogenetic analysis of the available plant CMO sequences and found solid evidence supporting that they form two clades, which we named CMO1 and CMO2. CMO1 proteins are present in every higher plant of known sequence, including Amaranthaceae species, while CMO2 is only present in all species studied from the Amaranthaceae family. Also, to explore whether there are structural differences between these two kinds of plant CMO enzymes that could cause possible differences in their substrate specificity, and given that no CMO three-dimensional structure is known, we used homology modeling, molecular dynamics (MD) and docking simulations to study the two CMO proteins from the high-GB-accumulator Amaranthaceae plant *Spinacia oleraceae* (*So*CMO1 and *So*CMO2). The active site aromatic and negatively charged residues that probably interact with the trimethylammonium group of choline in *So*CMO2 are conserved in every CMO2 sequence but absent in the CMO1 sequences, a finding that strongly suggests that the physiological substrate of the latter enzymes is not choline but a still unknown compound.

## Methods

### Sequence analyses and phylogenetic studies

CMO amino acid sequences were retrieved by BLAST searches [23] from the non-redundant (NR) collection at the NCBI site (http://blast.ncbi.nlm.nih.gov/Blast.cgi) [24] or on Phytozome v12.1.6 database (https://phytozome.jgi.doe.gov) [25], using the spinach CMO (NCBI accession number XP_021849509) as bait. Progressive multiple amino acid sequence alignments were performed with ClustalX 2.0 [26] and were corrected manually using BioEdit [27] (http://www.mbio.ncsu.edu/bioedit/bioedit). For each retrieved CMO protein we performed the identification of their conserved domains using the NCBI’s Conserved Domain Database (CDD; http://www.ncbi.nlm.nih.gov/cdd/) [28]. Only those sequences that have the cd03541 Rieske domain and the cd08883 mononuclear non-heme iron domain were considered as CMOs in our study (see Results section). When a retrieved amino acid sequence was incomplete or showed atypical insertions or deletions, the genomic or cDNA sequence for this protein was retrieved and the gene prediction software, Softberry FGENESH+ [29] (http://www.softberry.com/) was used to re-predict intron/exon gene structure, taking into account protein homology information from the more similar known complete CMO sequence. Likewise, if a *CMO* gene was not reported as such in a fully sequenced plant genome of the Phytozome database, a TBLASTN search [23] was performed to locate putative genomic sequences containing this *CMO* gene. Then these sequences were analyzed using FGENESH+ to predict the *CMO* gene structure. The corrected or newly predicted CMO amino acid sequences were included in the final multiple amino acid sequence alignment.

Phylogenetic analysis was carried out with MEGA7 [30]. Phylogenetic relationships were inferred by using the Maximum Likelihood. Initial phylogenetic trees for the heuristic search were obtained automatically by applying Neighbor-Join and BioNJ algorithms to a matrix of pairwise distances estimated using a JTT model, and then selecting the topology with superior log likelihood value. The amino acid substitution model of Whelan and Goldman [31], using a discrete Gamma distribution with 5 categories, was chosen as the best substitution model, since it gave the lowest Bayesian Information Criterion values and corrected Akaike Information Criterion values in MEGA7 [30]. The gamma shape parameter value (+G parameter) was estimated directly from the data with MEGA7. The rate variation model allowed for some sites to be evolutionarily invariable. Confidence for the internal branches of the phylogenetic trees was determined through bootstrap analysis with 500 replicates. The same strategy was used to separately infer the evolutionary history of the Rieske and the mononuclear non-heme iron domains of the CMO proteins. With this aim, we identified the amino acid residues at the beginning and end of both domains in the spinach CMO (XP_021849509) sequence and then, based in the alignment of the complete sequences, we split all CMO sequences in their two domains, ensuring that both domains were complete. Consensus sequences were obtained from the multiple sequence alignments with a cut-off of 60% conservation. The software ESPript 3 [32] was used to show the results of the sequence consensus analysis. Sequence logos of selected active-site residues were constructed using the WebLogo server [33] (http://weblogo.threeplusone.com).

### Homology modeling

To select the crystal structure to be used as template in the generation of the CMOs homology models we performed multiple amino acid sequence alignments with the bacterial Rieske/mononuclear non-heme iron oxygenases with a reported crystal structure, using the L-INS-i method in the MAFFT program [34]. Once the template was selected, the starting model for *So*CMO2 as a trimer was prepared with MODELLER v9.14 [35]. This model was improved subjecting it to approximately 200 ns of molecular dynamics (MD) simulation, which was performed on GROMACS 4.6.5 software with the AMBER99SB-ILDN force-field and using an explicit TIP3P water periodical box with roughly 0.15 M NaCl adjusted to a neutral system. Bond constraints were imposed using the LINCS algorithm to allow a 2 fs integration interval. Electrostatics were handled with the Particle Mesh Ewald (PME) method. The system was maintained at 313 K with a velocity-rescaled Berendsen thermostat and at 1 atm with a Berendsen barostat to provide a constant-temperature, constant-pressure ensemble (NPT). The relaxed model was retrieved by clustering the last 5 ns of simulation, and its geometry was taken to equilibrium by energy minimization. The *So*CMO2 model was used as template to obtain the homology model of the *Arabidopsis thaliana* CMO1 (*At*CMO1), and this model was in turn used as template to obtain the model for the *So*CMO1 sequence. The initial models of the two latter proteins were prepared with MODELLER v9.14 and then subjected to approximately 800 ns (*At*CMO1) or 60 ns (*So*CMO1) of MD simulation using AMBER force-fields and energy minimization, as described above. Disordered regions at the N-terminal of the proteins were predicted using the program IUPred [36].

Topologies for the [2Fe-2S] Rieske and mononuclear non-heme iron centers were built using QM calculations (Hartree-Fock 6-31G**; GAUSSIAN 09) [37], ACPYPE [38] and AmberTools software [39], as reported by Mitra et al. (2013) [40]. To better represent the geometrical and chemical features of the mononuclear non-heme iron, a dioxygen and a water molecule were linked to it. QM calculations were done with the low-spin configuration for an oxidized Rieske [2Fe-2S] center [41], and with and intermediate number of unpaired spin electrons in the case of the Fe^3+^/O_2_/H_2_O mononuclear non-heme Fe center [42]. The validation of these topologies was performed analysing the geometrical fluctuations in short simulations (1 ns) of an isolated molecule comprising the [2Fe-2S] Rieske and its covalently linked amino acid residues, or the mononuclear non-heme Fe and its covalently linked amino acid residues. The final topologies of the [2Fe-2S] Rieske center and mononuclear non-heme iron were manually merged with the models final subunit topology, and duplicated declarations were deleted. The Rd.HMM protocol [43] was used to test the appropriateness of the models backbones as hosts for the *So*CMO2, *At*CMO1 and *So*CMO1 sequences. Briefly, this protocol retrieves from the UniProtKB/Swiss-Prot protein sequence database all sequences that are structurally compatible, in a higher or lesser degree, with the backbone of the homology model tested without considering the sequence information in the PDB. For a good quality model, the protein sequence with the highest score, i.e. the first hit, should correspond to the protein modelled [43]. The Rosetta energy function was also used for evaluation of the models. Structural alignments of the models were performed using the Structural Alignment of Multiple Proteins (STAMP) tool in the VMD software [44].

### Docking simulations

A flexible choline molecule was docked into the active site of the rigid *So*CMO2 model with AutoDock Vina [45], using a box of 10 Å^3^ centered near the mononuclear iron. Sixty poses were visually inspected to select the one that fulfilled the criterion of having a proper orientation of the choline hydroxyl group with respect to the mononuclear non-heme iron, as it would be needed for the reaction to take place. Figures of the homology models and docking simulations were made using the UCSF Chimera software [46]

## Results

### Plant CMOs are bidomain enzymes that form a monophyletic protein subfamily

All identified CMO proteins possess a Rieske-type domain at their N-terminal half and a mononuclear non-heme iron domain at their C-terminal half. Similar domain architecture of the CMO proteins can be observed in several bacterial oxygenases that catalyze the oxidation of a variety of hydrophobic, mainly aromatic, compounds by the insertion of one or two hydroxyl groups [47]. According to the Conserved Domain Database (CDD), the N-terminal Rieske domain of CMO proteins belongs to the cd03541 (Rieske_Ro_Alpha_N_CMO) subfamily of the Rieske_Ro_Alpha_N protein family (cd03469), and their C-terminal catalytic domain belongs to the cd08883 (Rho_Alpha_C_CMO-like) subfamily of the Rho_Alpha_C protein family (cd00680). Indeed, all putative CMO sequences retrieved from the NCBI and Phytozome databases, using the amino acid sequence of *Spinacia oleracea* CMO XP_021849509 as bait, have, without exceptions, an N-terminal domain that belong to cd03541 subfamily and a C-terminal domain that belong to the cd08883 subfamily. Therefore, only those protein sequences with these two domains were used for our sequence and phylogenetic analyses. With this criterion, a total of 167 protein sequences (S1 Table) were selected. Although of all them have both domains, some sequences were incomplete or appear to have atypical insertions or deletions; these sequences were revised and most of them were corrected following the procedure described in the Methods section. These corrected or newly predicted CMO amino acid sequences were also included in the final multiple amino acid sequence alignment (S1 Fig). In a few cases, the FGENESH+ program confirmed that the insertion or deletion was correct and therefore the original reported sequences were used in the multiple amino acid alignment, where they introduced gaps (S1 Table). Because of these gaps, there were a total of 743 positions in the final alignment used for the ensuing phylogenetic analysis.

**Fig 2:**
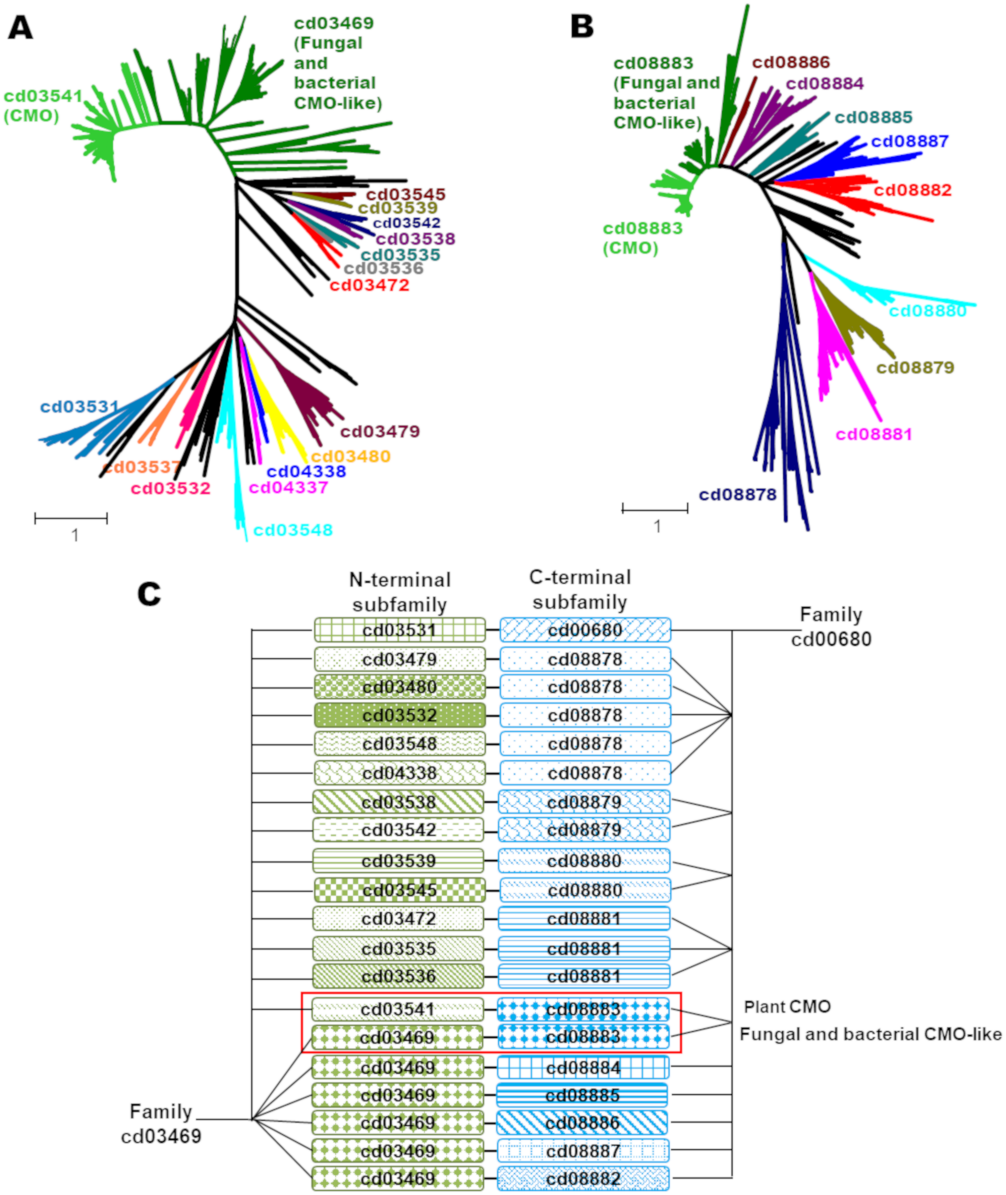
Phylogenetic trees of the bidomain Rieske/mononuclear non-heme iron protein sequences. (A) Tree of the N-terminal Rieske domain of retrieved protein sequences identified at the CDD as members of the oxygenase alpha-subunit N-terminal Rieske domain protein family (RHO_alpha_C, cd03469) or any of its protein subfamilies identified with different cd numbers. The Rieske domain of the bacterial CMO-like (dark green branches) has not a subfamily cd number assigned yet, as well as the Rieske domains of other proteins (black branches). (B) Tree of C-terminal mononuclear non-heme iron domain of retrieved sequences identified at the CDD as members of the oxygenase alpha subunit C-terminal catalytic domain protein family (Rieske_RO_Aplha_N, cd00680), or any of its subfamilies. As in (A) subfamilies to which a cd number has not been assigned yet are depicted as black branches. With the exception of CMOs, all retrieved sequences were bacterial. (C) Schematic representation of the association of the Rieske domains with the mononuclear non-heme iron domains in the retrieved proteins.

To test whether the CMO subfamily is monophyletic, the N-terminal Rieske domains of the CMO sequences were aligned with the Rieske domains identified at the CDD as members of the Rieske_Ro_Alpha_N protein family (cd03469), including its respective protein subfamilies. In the same way, the C-terminal mononuclear non-heme iron domains of the CMO sequences were aligned with the other mononuclear non-heme iron domains identified at the CDD as members of the Rho_Alpha_C family (cd00680) and its respective protein subfamilies. The results of these analyses showed that the two domains of all retrieved CMO sequences, without exception, belong to subfamily cd03541 and subfamily cd08883 and are from eukaryotes (Fig 2A and 2B). Interestingly, CMO sequences were identified also in chordate animals: *Saccoglossus kowalevskii* (acorn worm; hemichordata; 3 sequences), *Branchiostoma floridae* (Florida lancelet; cephalochordata; 3 sequences) and *B*. *belcheri* (Belcher’s lancelet; cephalochordata; 4 sequences), as well as in two protists: *Nannochloropsis gaditana* (microalgae; heterokonthophyta; 1 sequence) and *Acanthamoeba castellanii* (Amoebozoa; 1 sequence). Since the genome of this last species (*A*. *castellanii*) suffered extensive lateral gene transfer through its evolution [48], it is probable that the presence of a CMO in this organism is also a consequence of a lateral gene transfer event. Likewise, a lateral gene transfer event could also explain the presence of CMO in *S*. *kowaleyskii*, *B*. *floridae* and *B*. *belcheri*, because CMOs were not found in any other animal species. Furthermore, CMO proteins were not found either in fungi or archaea, but we retrieved sequences from fungi and bacteria having a C-terminal domain that belongs to the cd08883 subfamily, as do the C-terminal domain of plant CMOs. However, the N-terminal domains (Rieske domains) of these fungal and bacterial proteins do not belong to the cd03541 subfamily, or to any of the other 15 numbered subfamilies of the Rieske_Ro_Alpha_N protein family (cd03469). They do belong to the comprehensive cd03469 family, but a subfamily cd number has not been yet assigned to them (Fig 2A and 2B). We named these proteins as fungal or bacterial CMO-like. Indeed, consistent with this finding, a putative CMO from the yeast *Pichia stipitis* has been reported and named CMO1 because of its homology to plant CMOs and because it appears to be essential for the growth of the yeast on choline as the only nitrogen source, although it was not proved whether choline is a substrate of this enzyme [49].

Other bacterial Rieske/mononuclear non-heme sequences have a variety of different Rieske and mononuclear non-heme domains, as depicted in Fig 2C. As shown in this figure, domain cd03451 is exclusive of plant CMO proteins, as is the particular association of domain cd03541 with domain cd08883; the domain shuffling events that have occurred in other Rieske/mononuclear non-heme monooxygenases were not detected in CMO proteins. The closer homologs of CMOs are the already mentioned fungal and bacterial CMO-like proteins, which have a mononuclear non-heme iron domain of the same subfamily (cd08883) as the one of plant CMO proteins but different Rieske domain. Thus, bacterial CMO-like, fungal CMO-like and plant CMOs proteins possibly share a common ancestor.

**Fig 3:**
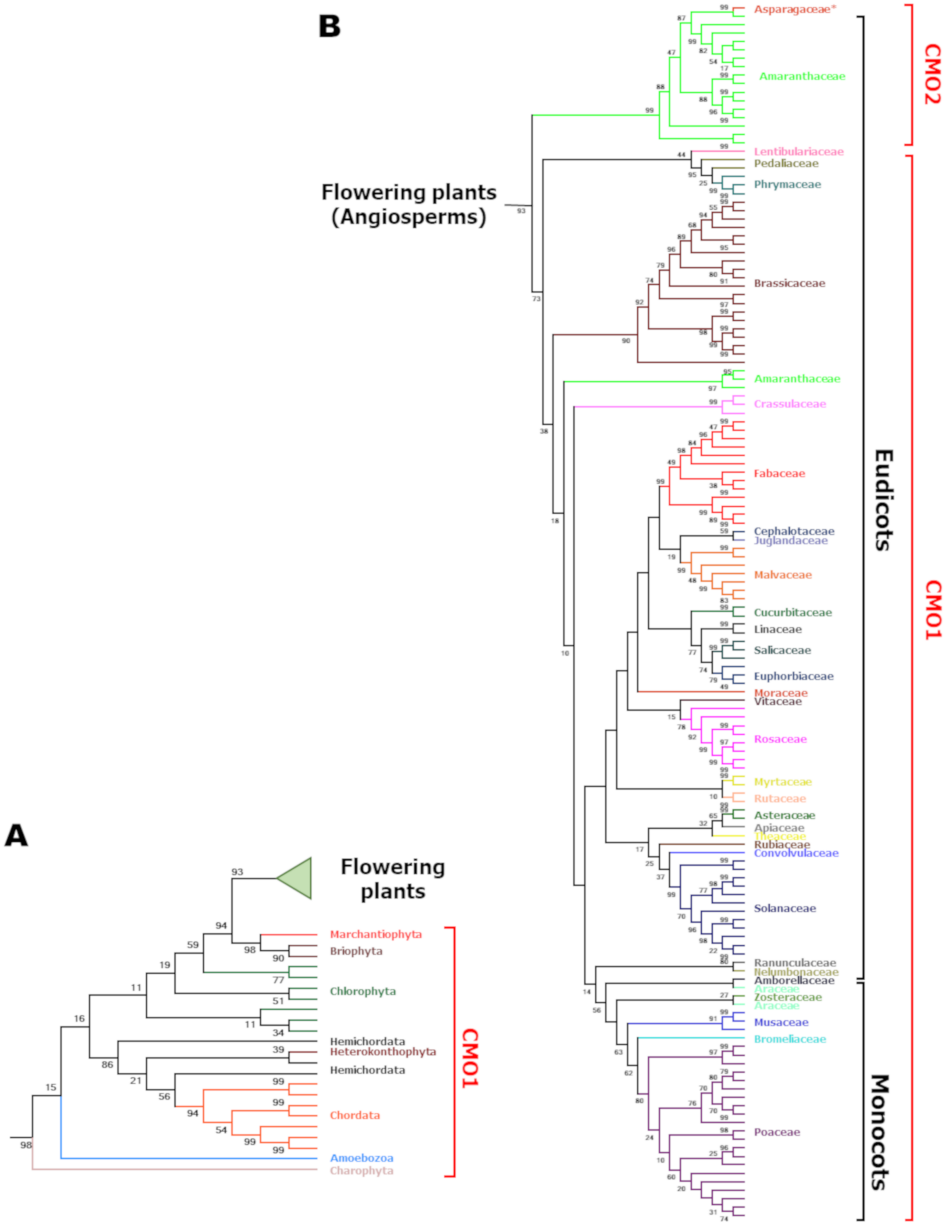
Phylogenetic tree of CMO proteins. (A) Sequences from primitive photosynthetic eukaryotes, chordates and protist species. (B). Flowering Plants. *The mononocot *Ophiopogon japonicus* (Asparagaceae) species groups with the dicot Amaranthaceae species within the CMO2 clade. The tree was inferred using the Maximum Likelihood method based on the Whelan and Goldman model [31] as described in the Methods section. The +*G*, parameter value was 2.0069 and the [+*I*] value was 0.94% of sites. Branches are colored according to the taxonomic family. The best tree with the highest log likelihood (-108,453.30) is shown. The percentage of trees in which the associated taxa clustered together in a bootstrap test (500 replicates) is shown next to the branches. The tree is drawn to scale and only the branch topology is shown, with branch lengths measured in the number of substitutions per site. The analysis involved 167 amino acid sequences (156 from plants and 11 from non-plant eukaryotic organisms) given in S1 Table.

**Fig 4:**
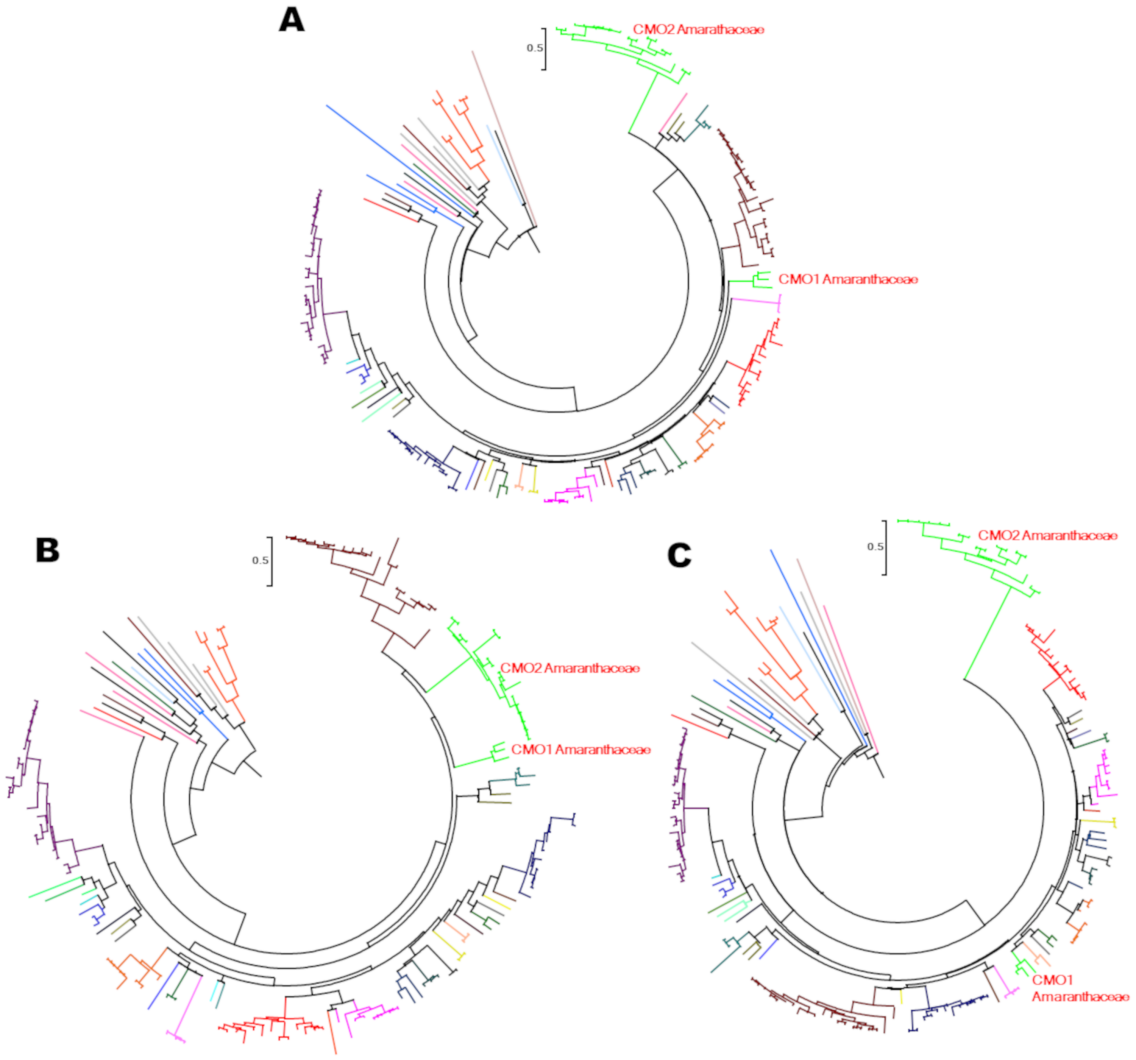
Evolutionary rate of the CMO proteins domains. The evolutionary history of the two domains of CMO proteins was separately inferred by splitting all CMO sequences in their two domains. The phylogenetic trees were obtained using the Maximum Likelihood method, as described in the legend of Fig 3. (A) Circle representation of the tree shown in Fig 3. (B) Circle representation of the phylogenetic tree of the CMO Rieske domain. The tree with the highest log likelihood (-39,113.47) is shown. The +*G*, parameter value was 0.7879 and the [+*I*] value was 0.91% of sites. (C) Circle representation of the phylogenetic tree of the CMO non-heme mononuclear iron domain. The tree with the highest log likelihood (-517,254.51) is shown. The +*G*, parameter value was 0.7024) and the [+*I*] value was 0.14% of sites. In the three panels, the CMO1 and CMO2 proteins of the Amaranthaceae family are indicated.

We performed multiple sequence alignments considering the identified eukaryotic CMO protein sequences that contain the pair domain cd03541/cd08883, and constructed the phylogenetic tree shown in Figs 3 and 4A and S2 Fig. As expected, flowering plants (angiosperms) form a well-supported monophyletic group, as well as all eukaryotic CMO protein sequences. Primitive photosynthetic eukaryotes with a known genome like *Nannochloropsis gaditana* (Heterokonthophyta), *Micromonas pusilla*, *Micromonas* sp. RCC299, *Coccomyxa subellipsoidea* C-169, *Bathycoccus prasinus* (all of them belonging to Chlorophyta), or *Klebsormidium flaccidum* (Charophyta) also contain a CMO enzyme. However, the genomes of the chlorophytes *Chlamydomonas reinhardtii*, *Volvox carteri*, *Ostrococcus tauri* and *O*. *lucimarinus* lack CMO sequences; therefore, secondary events of loss should be postulated to explain *CMO* gene absence in these species. Thus, although genes coding for CMO proteins are present in a few eukaryotic organisms different from photosynthetic eukaryotes ([49] and this work), they more likely resulted from horizontal gene transfer events and therefore it can be safely concluded that CMO proteins containing the pair domains cd03541/cd08883 exclusively evolved in photosynthetic eukaryotes.

The CMO phylogenetic tree shows two clades: one containing CMO proteins from all plant families that have at least one species with a sequenced *CMO* gene, including Amaranthaceae, and another one including only CMO proteins from Amaranthaceae species, which most likely resulted from a duplication gene event. One of the two duplicates evolved rapidly, significantly diverging from the other and eventually forming a different clade with a good bootstrap support (94%) (Figs 3 and 4A). We named this clade as CMO2 to differentiate it from the other clade, which includes all other plant CMO proteins, hereafter referred as CMO1. Sequence identity between the proteins grouped in clade CMO1 is higher than 50%, and between proteins of the clade CMO2 is higher than 85%, while sequence identity between CMO1 and CMO2 proteins is 30% at the most. Our results indicate that during plant evolution after monocot-eudicot divergence several other independent *CMO* gene-duplication events occurred in 9 plant families different from Amaranthaceae. Specifically, we found duplicates in *Camelina sativa*, *Arabis alpina*, *Raphanus sativus*, *Kalanchoe laxiflora*, *Phaseolus vulgaris*, *Gossypium hirsutum*, *Linum usitatissimum*, *Malus domestica*, *Nicotiana*, *tabacum*, *Panicum virgatum*, *Erythranthe guttata*, *Solanum penelli* and *Solanum lycopersicum*. However, these other duplication events are recent, as indicated by the high sequence identity between the proteins encoded by the duplicated genes.

CMO from *Ophiopogon japonicus* (Asparagaceae, a monocot) has higher amino acid sequence identity with CMO2 proteins from the Amaranthaceae family than with CMO1 from monocots (Fig 3 and S2 Fig). This unexpected position of the CMO from *O*. *japonicus* was also reported by Joseph *et al*. [18]. Interestingly, we have previously found that the *O*. *japonicus* ALDH10 sequence clusters with the ALDH10 proteins of the Amaranthaceae 2 clade, which includes enzymes with proved or predicted betaine aldehyde dehydrogenase activity [13]. One possible explanation for these findings is that both genes were acquired by *O*. *japonicus* by horizontal gene transfer from an unidentified Amaranthaceae, a possibility supported by the fact that horizontal gene transfer is a significant force in the evolution of plant genomes [50,51]. Further studies are needed to provide evidence in favor or against this possibility.

### The non-heme monuclear iron domains of CMO1 and CMO2 proteins evolved at different rates

To explore whether the Rieske and the non-heme mononuclear Fe domains of the plant CMO proteins evolved at the same or at a different rate, we constructed phylogenetic trees of each of these two domains separately (Fig 4B and 4C). We found that within the CMO1 sequences the rate of evolution of both domains was the same, and comparable to the rate of evolution of the Rieske domain of the CMO2 proteins, but the non-heme mononuclear domains of the CMO2 proteins appears to have evolved at a much faster rate, as can be inferred by the length of their branches (Fig 4C). These findings suggest that the catalytic domain of the CMO2 proteins was under some kind of evolutionary pressure that probably led to the acquisition of new substrate specificity, as it will be shown below.

**Fig 5:**
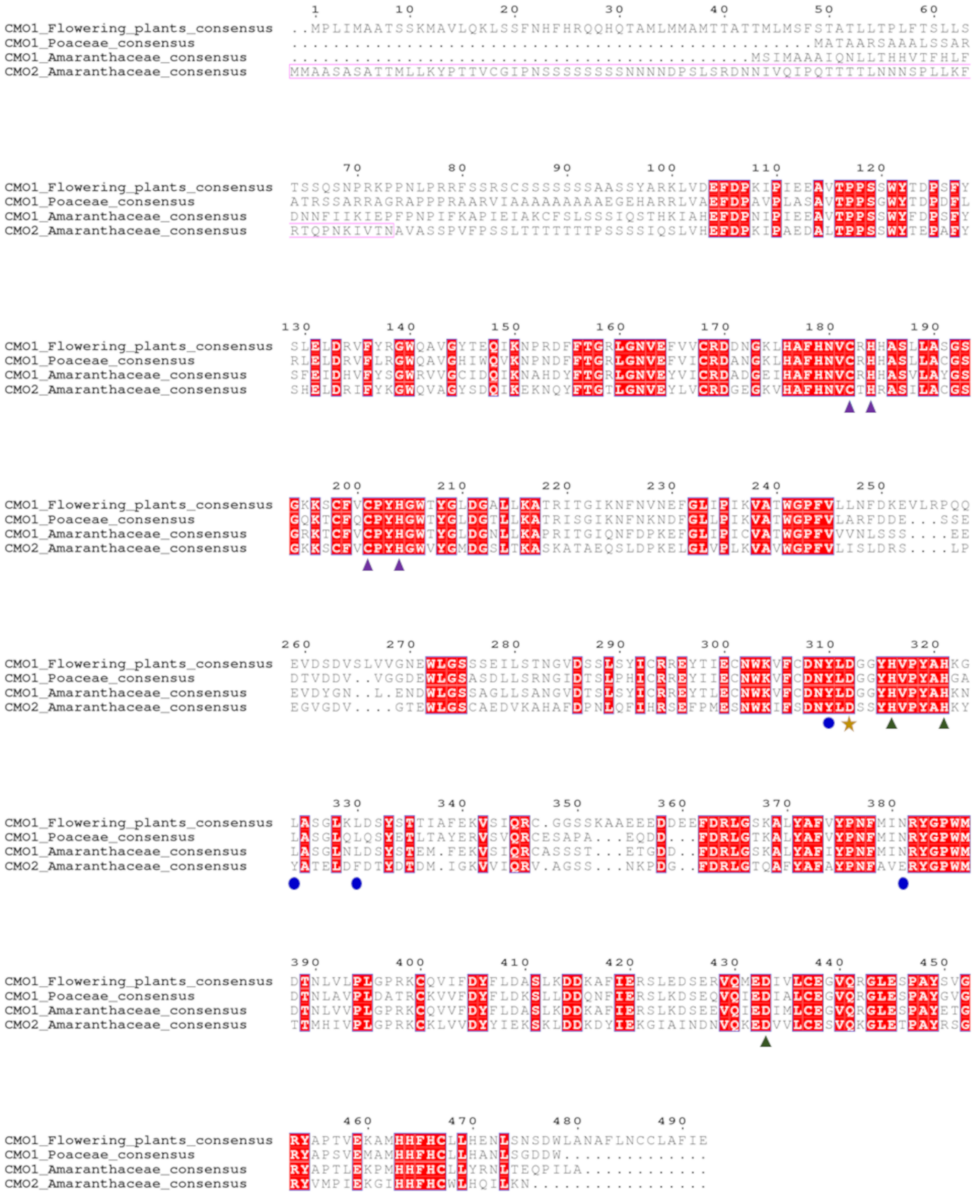
Alignment of CMO consensus sequences of all CMO1 proteins, CMO1 proteins from the Poaceae and CMO1 and CMO2 proteins from the Amaranthaceae. To obtain the consensus sequences we used a cut-off of 60% identity. Residues in red boxes are conserved in at least 60% of all sequences. Residues marked with a purple triangle are those involved in coordinating the iron atoms of the Rieske center; residues marked with green triangles are involved in coordinating the non-heme mononuclear iron; the residue marked with a gold star is the aspartate involved in electron transfer between the Rieske and non-heme mononuclear iron centers; residues corresponding to putative key active site residues involved in choline binding in CMO2 enzymes, as shown below in the docking simulations, are marked with blue circles. The sequence enclosed in a purple frame corresponds to the predicted chloroplast signal peptide in CMO2 enzymes.

The alignments of CMO1 and CMO2 consensus amino acid sequences (Fig 5) show differences between them scattered throughout the entire sequences. The possible chloroplast signal peptide, which was proposed to be located at the N-terminal region in the *So*CMO2 protein [14], is well conserved in CMO2 sequences but not in the CMO1 ones. Moreover, alignments of the consensus CMO1 sequences with the consensus CMO1 and CMO2 sequences from Amaranthaceae and CMO1 sequences from Poaceae (Fig 5) showed that the N-terminal region of the CMO1 proteins from these two families is shorter than that of the rest of the CMO1 proteins. The amino acids involved in the Rieske center and in binding the mononuclear non-heme iron, as well as the aspartate residue that participate in the electron transfer between the two, are fully conserved in all CMO1 and CMO2 proteins, indicating that all of them are active oxygenases.

### The homology models of *So*CMO2 and *So*CMO1 proteins show important differences between them

Given that to date no three-dimensional structure of any CMO protein has been reported, to analyze structural features that could provide insight into the possible differences in substrate specificity between CMO1 and CMO2 proteins, we constructed and analyzed homology models of the *So*CMO2, *So*CMO1 and of the CMO1 from *Arabidopsis thaliana* (*At*CMO1). We also built topologies for the [2Fe-2S] Rieske and mononuclear non-heme iron centers, which correctly predicted the geometries found in X-ray structures of oxygenase enzymes with the same kinds of iron centers.

**Fig 6:**
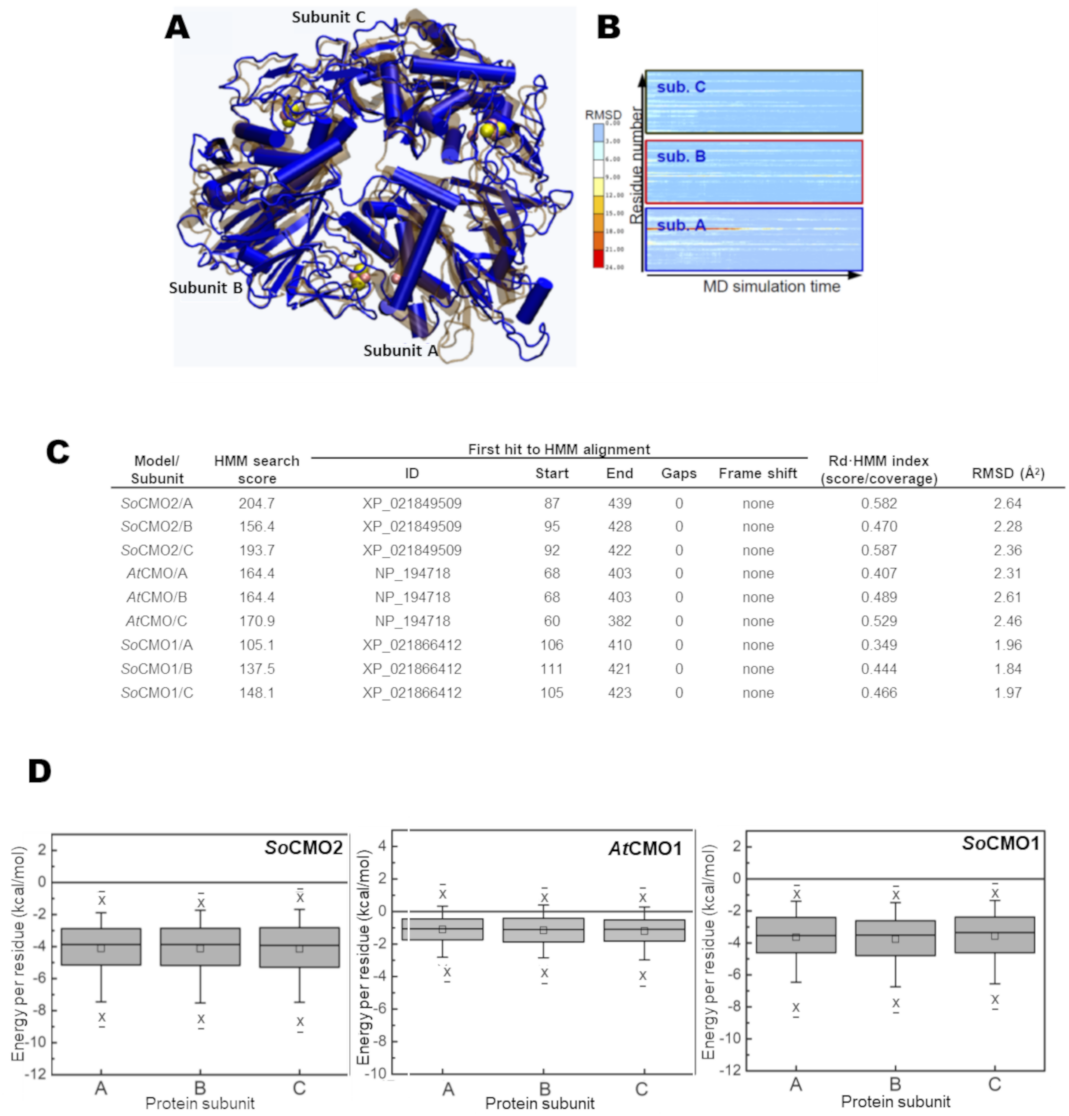
Qualitative evaluation of the final homology models. (A) Three-dimensional predicted structure for *So*CMO2 after 200 ns of MD simulations is shown as blue cartoons and the prediction before MD simulations is shown superimposed as ocher translucent cartoons. Iron and sulfur atoms are shown as orange and yellow spheres, respectively. (B) Heat maps showing the changes in RMSD relative to the final structure per residue of each subunit along MD simulations. The bar plot on the left shows the color range associated with the RMSD values, from red (high) to white (medium) to blue (low). (C) Results of the Rd.HMM analysis of the final *So*CMO2, *So*CMO1 and *At*CMO1 homology models shown per subunit. Note that in this kind of analysis a Rd-HMM index value of 0.6 is obtained for structures solved by X-ray crystallography [42]. The RMSD values given are relative to the bacterial monoxygenase (PDB: 3N0Q) used as template for modeling *So*CMO2 and were calculated using STAMP [43] to match equivalent positions in the structures. (D) Box plots of the energy per residue of the *So*CMO2, *So*CMO1 and *At*CMO1 homology models. The evaluation was performed using the Rosetta energy scoring function.

We started building the *So*CMO2 model because this is the best characterized CMO to date. This model was made without the putative chloroplast peptide signal, which was proposed to consist of 60 residues [14], and without the next segment of 19 residues, which scored as highly mobile in order/disorder predictions [35]. Therefore, the model considers a sequence that is 79 residues shorter than the complete one, i.e., from residues 80 to 439, excluding in this way the highly variable (poorly conserved) region of CMO proteins. From sequence alignment analysis we conclude that the best template to build the *So*CMO2 model was the X-ray structure of a bacterial Rieske-mononuclear non-heme dioxygenase from *Rugeria sp*. (PDB: 3N0Q), which has only 24% identity with *So*CMO2; other Rieske/mononuclear non-heme oxygenases have even lower identity with *So*CMO2. This low degree of identity between the protein model and the protein being modeled made very difficult the construction of a good quality model. Nevertheless, after following the steps described in the Methods section, we obtained a *So*CMO2 homology model that complied with the expected functional requirements for a Rieske/mononuclear non-heme oxygenase enzyme. As the first *So*CMO2 model obtained with MODELLER was not satisfactory, we improved its quality by repeated MD simulations. A comparison of the final *So*CMO2 model with the initial one is shown in Fig 6A and the evolution of the model along the MD simulation, as assessed by the Root Mean Square Deviation (RMSD) per residue between the starting and final models, is shown in Fig 6B.

As an example of CMO1 proteins, and because at the time that we were doing these experiments the *So*CMO1 sequence was not yet available, we then followed the same procedure to obtain the *Arabidopsis thaliana* (*At*CMO1) homology model using as template the, *So*CMO2 model. The *At*CMO1 model was constructed from residue 58 to residue 422, eliminating the N-terminal region that contains a putative signal peptide (47 residues) and an additional 11 residues that were predicted to be disordered. When we had a reasonable good *At*CMO1 model, we became aware that the *So*CMO1 sequence had been reported and so we decided to use this *At*CMO1 model as template to make the *So*CMO1 3D-structure prediction. For the same reasons as before, only residues 103 to 426 of the complete *So*CMO1 sequence (426 residues) were included for the model. Considering this region, *At*CMO1 and *So*CMO1 share a 63% of sequence identity.

Analyzing the three homology models with the Rd.HMM protocol [42] we found that, out of all sequences in the NCBI-RefSeq database, the *So*CMO2 amino acid sequence (XP_021849509), the *At*CMO1 amino acid sequence (NP_194718) and the *So*CMO1 amino acid sequence (XP_021866412) were the first retrieved when using the *So*CMO2, *At*CMO1 and *So*CMO1 homology models, respectively, indicating that the models indeed can accommodate the respective sequences better than the sequences of any other protein in the NCBI RefSeq database. The results of the validation of the homology models shown in Fig 6C indicate an appropriate backbone for all models, with a Rd.HMM index value just slightly below the value reported for X-ray crystallography determined structures [43]. In addition, the quality of the structural features of the models was tested using the Rosetta-energy scoring function. The box plots of the energy per residue (Fig 6D**)** show the absence of abnormally strained, high-energy residues, and that the overall energy of the models was negative.

**Fig 7:**
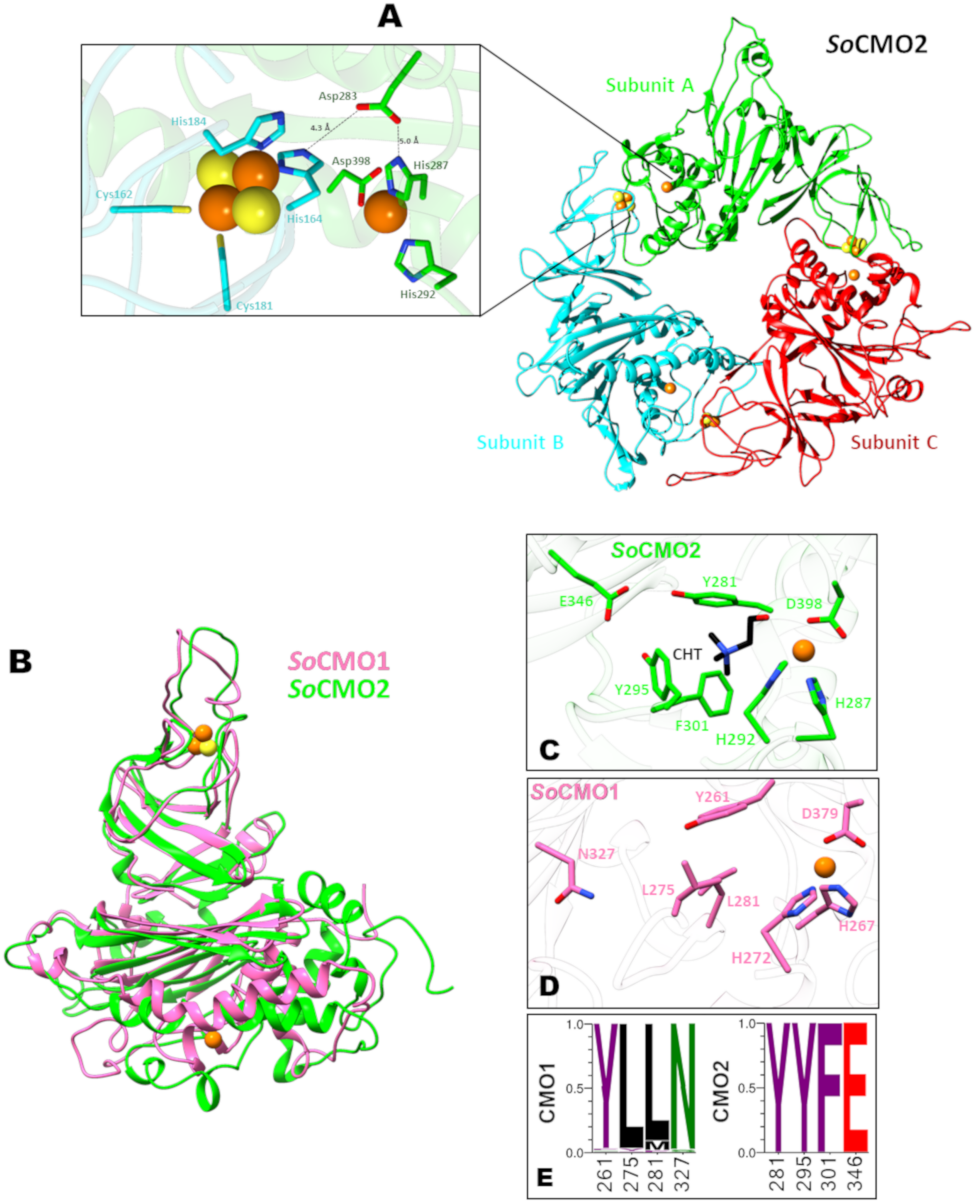
Homology models of *So*CMO1 and *So*CMO2 proteins. (A) Cartoon representation of the *So*CMO2 trimer with each subunit in a different color; the close-up shows the residues involved in iron coordination in the Rieske center (Cys162, His164, Cys181, His184) and in the non-heme mononuclear iron (His287, His292, Asp398), as well as the aspartate residue involved in the transfer of electrons between the two iron centers (Asp283). (B) Cartoon representation of aligned *So*CMO1 (pink) and *So*CMO2 (green) monomers. In panels A and B, carbon atoms are colored in pink (*So*CMO1) or green (*So*CMO2), oxygen atoms in red and nitrogen atoms in blue and iron and sulfur atoms are shown as spheres colored orange and yellow, respectively. (C) Cartoon representation of the modeled *So*CMO2A active site showing a choline molecule (black carbon atoms) docked into it. The choline trimethylammonium group is in a favorable position to interact with the aromatic box formed by residues Tyr281, Tyr295, Phe301, as well as with the near Asp346 residue. The choline hydroxyl group is oriented towards the iron atom as needed to establish a coordination bond with it. (D) Cartoon representation of the modeled *So*CMO1 active site, showing the putative active site residues at position equivalent to the ones in the *So*CMO2 model. (E) Sequence logos of selected positions of CMO1 and CMO2 proteins showing the conservation of residues putatively involved in choline binding in CMO2. Residue conservation is shown as probability. The amino acids color scheme of the logos is according to their chemical properties: polar (S, N), green; aromatic (Y, F), purple; acidic (E), red; and hydrophobic (L, M), black. Residue numbers correspond to the complete *So*CMO2 sequence (XP_021849509) or to the complete *So*CMO1 sequence (XP_021866412), as appropriate.

In the three homology models, the residues involved in iron coordination in the Rieske and mononuclear non-heme centers are properly positioned and the distance between them was similar to the one found in the known crystal structures and the appropriate one for the transfer of electrons from the Rieske to the mononuclear non-heme iron center, as exemplified in Fig 7A with the *So*CMO2 model. Also, the carboxyl group involved in the transfer of electrons between the iron centers in the Rieske/mononuclear non-heme oxygenases [52], which belongs to the side chain of an aspartate in CMO enzymes, was correctly positioned to perform this function. A comparison of the subunit A backbone of the *So*CMO2 and *So*CMO1 models is shown in Fig 7B.

In the active site of the *So*CMO2 model (Fig 7C)—located in the mononuclear non-heme Fe domain as in every Rieske/mononuclear non-heme oxygenase—we found three residues, Tyr281, Tyr295, and Phe301, forming an aromatic box of the kind that has been found in other proteins to be involved in binding a trimethylammonium group, as the one present in choline, through cation-π interactions [16,53–55]. In addition, there was a glutamate residue, Glu346, which could be also involved in an ionic interaction through its side chain carboxyl group with the positively charged trimethylammonium group, as was also found in some choline/acetylcholine binding proteins [55,56]. Indeed, the presence of these aromatic residues and the carboxylate group has been considered a evolutive convergent trait critical for choline binding [56]. Interestingly, except for Tyr281, conserved in most CMO proteins (see below), in the modeled active site of *So*CMO1—as well as in that of the *At*CMO1 model (not shown)—there are no negatively charged or aromatic residues. In *So*CMO1, Glu346 is Asn327, Tyr295 is Leu275, and Phe301 is Leu281 (Fig 7D). These active-site residues changes strongly suggest that both *So*CMO1 and *At*CMO1would have a very low affinity for choline, if any. The hydrophobic nature of the *So*CMO1 and *At*CMO1 active sites, as well as their ample space, suggests that a nonpolar, aromatic compound could be the physiological substrate of these enzymes.

### A choline molecule fits well in the active site of *So*CMO2

We predicted the possible position and contacts of choline bound into the active site of the *So*CMO2 homology model by performing docking simulations, as described in the Methods section. The most populated cluster of choline poses was observed close to the mononuclear non-heme iron center and the three coordinated iron ligands (His287, His292 and Asp398). We selected the best pose (depicted in Fig 7C) by visual inspection and using the orientation of the hydroxyl group of choline relative to the mononuclear non-heme iron as an additional criterion. In the selected pose this hydroxyl group is close to the iron though not near enough to form a coordinated covalent bond, which is not unexpected because the AutoDock Vina forcefield uses molecular mechanics and it cannot predict the formation or breaking of bonds. Interestingly, in this docking pose the quaternary ammonium of choline is inside the aromatic box formed by Tyr281, Tyr295 and Phe301 and near Glu346, as we expected. The three aromatic rings provide an environment rich in electron-density and may participate in cation-π interactions with the trimethylammonium group, whereas the negative charge of the neighbor Glu346 should also compensate the quaternary ammonium electric charge. In addition, this docking pose is consistent not only with the binding of choline but also with the binding of the molecular dioxygen needed for the reaction to take place, since the adduct with choline apparently would not obstruct dioxygen binding. In summary, the docking pose shown in Fig 7C is consistent with what should be expected for a pre-catalytic enzyme-substrate complex for a *bona fide* CMO.

### Putative critical active site residues are conserved within the two kinds of CMO enzymes

**Fig 8:**
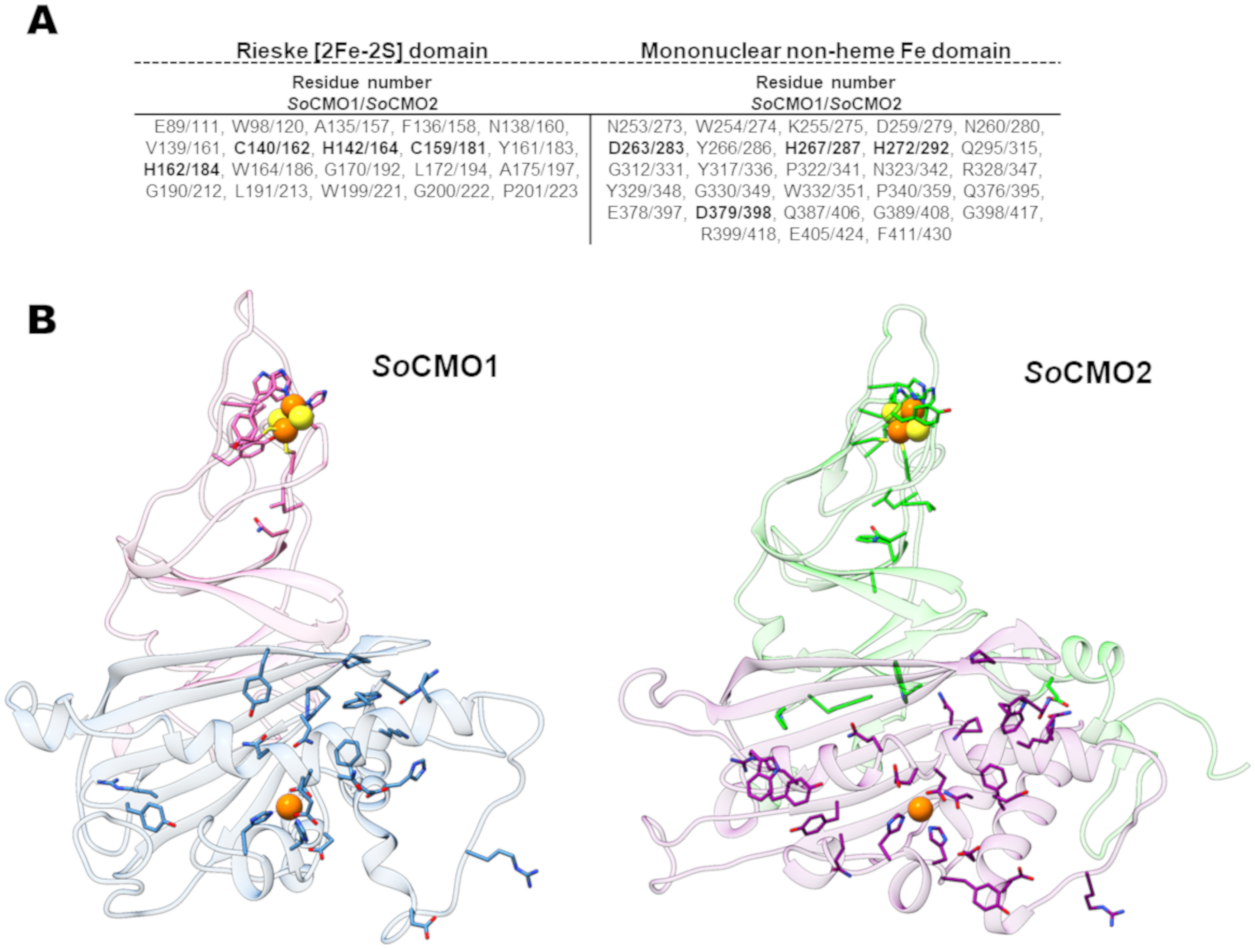
Totally conserved residues in CMO proteins. (A) List of the totally conserved residues in the Rieske [2Fe-2S] and the mononuclear non-heme iron domains of all analyzed CMO proteins. Residues involved in coordination of the Rieske and mononuclear non-heme irons, as well as the aspartate involved in the transfer of electrons between the two iron centers, are in bold. Numbering is according to *So*CMO1 and *So*CMO2 complete sequences. (B) Cartoon representation of a monomer of the *So*CMO1 and *So*CMO2 homology models showing the location of the totally conserved residues in the predicted three-dimensional structure. Rieske domain and mononuclear non-heme iron domains are colored pink/blue and green/purple in the *So*CMO1 and *So*CMO2 monomers, respectively. Residues are shown as sticks, with nitrogen atoms colored in blue, oxygen in red, iron (shown as a sphere) in orange and sulfur (shown as a sphere) in yellow.

Interestingly, three of the residues suggested by the *So*CMO2 homology model to be in the active site—Tyr295, Phe301 and Glu346 (*So*CMO2 numbering)—are conserved in every known CMO2 sequence, while the residues at equivalent positions in *So*CMO1—Leu275, Leu291 and Asn327 (*So*CMO1 numbering)—are also highly conserved in all known CMO1 sequences (Fig 7E). Out of the 150 plant sequences included in the CMO1 clade, only in one of the duplicates from *E*. *guttata*, *S*. *penelli* and *S*. *lycopersicum* Leu275 has been changed for a phenylalanine (in *E*. *guttata*) or tyrosine (in *S*. *penelli* and *S*. *lycopersicum*). Also, only in these three enzymes Asn327 has been changed for a serine residue. The conservation of such different active site residues within the two kinds of CMO enzymes strongly suggests differences between them in substrate specificity. In addition to these active-site residues and to those involved in coordinating the mononuclear non-heme iron, there are several other residues totally conserved among the CMO enzymes in both the Rieske and mononuclear non-heme iron domains (Fig 8A). Their location in the *So*CMO1 and *So*CMO2 homology models is depicted in Fig 8B). These conserved residues may be important for activity (particularly, for binding of the Fd protein and/or electron transfer) and/or for the stability of the native trimeric structure. In addition, the active-site tyrosine at position 281 in *So*CMO2 is highly conserved in plant CMO proteins; only in one of the CMO1 duplicate of *E*. *guttata* and the CMO1 of *Capsella rubella* the residue at this position has been changed to phenylalanine. The aromatic nature of this residue at this position is not conserved in other Rieske/mononuclear non-heme oxygenases (results not shown), which suggests that, although it is not needed for the oxygenase activity, it may be important for substrate specificity, not only of the CMO2 but also of the CMO1 enzymes.

## Discussion

During evolution of land plants some of them gained the ability to synthesize the osmoprotectant GB, which allowed their growing in very adverse environments where saline soils are prevalent, like in coastal highly saline lands, or in dry or cold climates. This metabolic trait confers a great adaptive advantage not only to halophytes plants, but also to mesophytes, which may experience sporadic episodes of water deficit or freezing temperature. As have been previously shown [16], the change of a single residue in some plant ALDH10 enzymes suffices to confer them BADH activity, which is a *sine qua non* condition for the plant to become a GB-accumulator. But the current experimental evidence obtained with wild-type and transgenic plants suggests that the acquisition of the ability to synthesize GB by evolving a BADH activity should have been accompanied by other adaptations, such as the gain of a significant CMO activity. In the present work we provided phylogenetic and structural evidence of how the CMO activity might have evolved in some of the CMO proteins.

The results of our phylogenetic analysis show that plant CMO proteins are monophyletic and clearly form two clades indicating the existence of two kinds of CMO proteins: CMO1 and CMO2. Limited phylogenetic studies have been previously reported [18,19,57] and one of them suggested the existence of two groups within the plant CMO proteins [18]. By the inclusion in our analysis of a much larger number of sequences (almost ten-fold more) we confirmed and better supported this observation. In addition, we found that the proteins belonging to the clade that we named CMO1 are present in every terrestrial plants having a known genome, regardless of its ability to synthesize and accumulate GB, while proteins of the clade that we named CMO2 were only found in the high GB-accumulator plants of Amaranthaceae species. CMO1 proteins are also present in primitive plants and other unicellular photosynthetic eukaryotes, which suggest that they are the closer descendants from to the ancestral plant CMO. Indeed, CMO2 proteins appear to have evolved from CMO1 as a consequence of a unique event of a *CMO1* gene duplication event that seems to have taken place early in the evolution of the Amaranthaceae family. Although we found other episodes of *CMO* gene duplication in other plants, these events seem to be more recent, as suggested by the few residues that have been changed in the resultant proteins, and probably they did not give rise to new functions. CMO2 proteins clearly evolved at a much higher rate than CMO1 proteins, which led to the formation of a new clade within the CMO subfamily. Of the two CMO protein domains, the mononuclear non-heme domain, where the active site is located, is the one that diverged faster. Moreover, the active site residues that probably are important for determining substrate specificity—as indicated by the homology models and docking simulations—are different in CMO1 and CMO2 enzymes, which strongly suggests functional differences between them. Based on the kind of these active site residues—aromatic and glutamate in CMO2 enzymes *versus* glutamine and non-polar, non-aromatic in CMO1 enzymes—, we think that only CMO2 are *bona fide* choline monooxygenases, *i*.*e*. their physiological activity is choline monooxygenase, while most of the CMO1s, including those of the Amaranthaceae species, are not. Indeed, our results hint at the possibility of CMO1s acting on aromatic substrates. It is probable that CMO1s retained the original and so far unknown physiological activity of the ancestor, an activity that seems important for the plant given the conservation of the *CMO* gene in every terrestrial plant of known genome. Interestingly, in the fungal and bacterial CMO-like enzymes the active site residues at the equivalent position of the ones that we propose in this work to be characteristic and possibly determinants of the substrate specificity of the CMO2 enzymes—Tyr295, Phe301 and Glu346 (*So*CMO2 numbering)—are changed to non-aromatic, non-charged residues identical or similar to the ones in CMO1 proteins, suggesting either that choline is not the substrate of none of these enzymes or that the reaction that they catalyze is different than the one catalyzed by CMO2 enzymes and requires a different mode of binding of choline at the active site. Unfortunately, none of the plant CMO1 or of the bacterial or fungal CMO-like proteins have been biochemically characterized as yet, so their substrate specificity and the reaction that they catalyze are unknown. The aromatic nature of the tyrosine residue at position 281 (*So*CMO2 numbering) is also conserved in the CMO-like proteins, which suggest that this aromatic residue is characteristic of the cd08883 domain and may be important for their general function. Regardless of their specificity, these findings suggest that photosynthetic eukaryotic CMO1 proteins and fungal CMO-like proteins evolved in parallel from an ancestral bacterial CMO-like protein, forming independent protein subfamilies with similar architecture and putative catalytic properties, which will be analyzed in a forthcoming work. On the other hand, it is also possible that CMO1 proteins do have a low CMO activity. Even more, this activity might have been present in the ancestral CMO1 protein. To date, most of the experimentally characterized enzymes exhibiting CMO activity belong to the CMO2 clade: spinach [10,14,58], sugar beet [21], and *A*. *caudatus* [21]. Only a recombinant CMO1 protein from barley has been assayed for CMO activity in the crude extracts of yeast cells where it was expressed and found that it can oxidize choline [19], but how the CMO activity level of this enzyme compares with that of CMO2 enzymes remains to be evaluated.

Many plants of the Amaranthaceae family are halophytes that tolerate salty and dry soils, and probably the ancestor that gave origin to this plant family also colonized osmotically stressed habitats. The acquisition of a functional CMO together with the acquisition of a functional BADH, which also occurs in the Amaranthaceae family [13], granted them a clear evolutionary advantage through the synthesis of glycine betaine. The evolutionary mechanism that gave rise to a functional CMO activity in these plants likely was the same as the one pointed out by others to explain similar acquisition of new enzymatic activities after a gene duplication event [59]: one of the two resulting *CMO* gene copies was free to undertake multiple mutations, which, under the strong pressure of the osmotically stressed environment, eventually lead to the acquisition and fixation of the CMO activity in these proteins. The low number of known CMO1 sequences from the Amaranthaceae family (just three sequences) contrasts with the higher number of known CMO2 sequences from the same family (17 sequences). This numerical discrepancy can be explained by the few known genomes of Amaranthaceae species to date (only those of the same three species) and by the fact that the *CMO2* gene sequences were obtained from cDNA when the plants were subjected to osmotic stress, indicating that in this family the *CMO2* gene, and not the *CMO1* gene, is the one up-regulated by these adverse environmental conditions. In these studies, the increase in the levels of the *CMO2* gene transcripts and/or CMO2 protein correlated with significant increases in the levels of GB, indicating that this is the protein that exhibits CMO activity and participates in the synthesis of this osmoprotector, at least in the high GB-accumulator species of this plant family. Indeed, osmotic stress response elements for salt, dehydration and cold were found in the *CMO2* gene promoter of the *Amaranthaceae* halophyte, GB-accumulator plant *Suaeda liaotungensis* [60].

Together, our results prompted us to propose as a hypothesis that only CMO2 proteins have CMO activity *in vivo* and that the CMO1 proteins do not. But then the question is, which enzyme produces betaine aldehyde from choline in those GB-accumulators plants that do not have the CMO2 protein and only have CMO1? Antibodies raised against CMO from spinach did not detect any protein in mangrove leaves (*Avicennia marina*) [61], barley (both monocot medium GB-accumulator plants) [19], sunflower and *Arabidopsis* (dicot GB-non-accumulator plants) [22], a finding that was taken as indicative of either significant structural differences between the spinach CMO and the CMO proteins of these plants, or of the absence of a CMO protein in these plants and the presence instead of a choline dehydrogenase (CDH) or choline oxidase protein [22]. However, the CMO protein used to raise the antibody was *So*CMO2; therefore, their results may be explained by the fact that all the other CMO proteins tested against this antibody were CMO1 proteins, which, as shown here, have low sequence identity with the CMO2 proteins. It seems therefore that the likely explanation of these results is the lack of cross-reactivity of the polyclonal anti-CMO2 antibodies against CMO1 proteins, due to the important differences between the two kinds of CMO proteins. Regarding the proposal of the possible existence of homologs of CDH or choline oxidase in land plants, we did not find any in pBLAST searches. It may be possible to hypothesize that another, non-yet described enzyme able to convert choline to betaine aldehyde was recruited for GB synthesis. In this respect, it results interesting that a recent report described a plant oxidase named GB1 that greatly increased the levels of GB when overexpressed in maize and soybean plants [62]. We found that GB1 enzymes are present in all plants of known genome (data not shown) and that they belong to the hydroxylase superfamily cl27195 (COG3000). Therefore, it could be speculated that they may participate in a biosynthetic route of GB forming the hydrate species of betaine aldehyde from choline, particularly when overexpressed, although this activity may not be their primary one. Unfortunately, their physiological substrates and the reaction that they catalyze are still unknown. Alternatively, CMO1s may have a vestigial CMO activity that could be exploited if they have access to choline in their cellular environment and their protein level and/or activity are up-regulated by osmotic stress. Indeed, this may be the case of the barley CMO1, which exhibited CMO activity as mentioned above [19], since the plant possesses an ALDH10 enzyme with proved BADH activity. But since barley CMO1 has been reported as peroxisomal and barley BADH as cytosolic, the different intracellular location of the two enzymes may impede their sharing of substrates or products.

## Supporting information

S1 Fig Complete alignment of CMO protein sequences. (PDF)

S2 Fig Unrooted phylogenetic tree of the complete CMO protein sequences given in S1 Table. (TIFF)

S1 Table Proteins identified as members of the CMO subfamily. (PDF)
