# Supplementary material for: Bona fide choline monoxygenases evolved in Amaranthaceae plants from oxygenases of unknown function: Evidence from phylogenetics, homology modeling and docking studies
Source: PLoS One. 2018 Sep 26;13(9):e0204711. doi: 10.1371/journal.pone.0204711 (PMC6157903; doi:10.1371/journal.pone.0204711)
Supplement: S1 Fig — (PDF) [file pone.0204711.s001.pdf]

Supplementary Material

Bona fide choline monoxygenases evolved in Amaranthaceae plants from oxygenases of unknown function: evidence from phylogenetics, homology modeling and docking studies

Javier Carrillo-Campos[1], Héctor Riveros-Rosas[2], Rogelio Rodríguez-Sotres[1], and Rosario A. Muñoz-Clares[1*]

[1]Departamento de Bioquímica, Facultad de Química, Universidad Nacional Autónoma de México, Ciudad de México, 04510. México.

[2]Departamento de Bioquímica, Facultad de Medicina, Universidad Nacional Autónoma de México, Ciudad de México, Ciudad de México, 04510. México.

***Correspondence:** Rosario A. Muñoz-Clares, Departamento de Bioquímica, Facultad de Química, Universidad Nacional Autónoma de México, Ciudad de México, 04510. México.

***E-mail:** clares@unam.mx

Tel: +52 5556223718

Running title: Evolution of choline monooxygenase proteins

```
                                                           1        10        20         30        40
BAC77698|Atriplex_nummularia                               .MAASA..TTMLLKYPTTVCGIPN.....SSSNNSTDPS....NNIVQTPQTTTT...NSPL
AGC13074|Atriplex_semibaccata                              .MAASA..TTMLLKYPTTVCGIPN.....SSSNNSTDPS....NNIVQIPQTTTT...NSPL
AHH24260|Atriplex_amnicola                                 .MAASA..TTMLLKYRTTVCGIPN.....SSSNNSTDPS....NNIVQIPQTTTT...NSPL
AFG28558|Atriplex_canescens__four-winged_saltbush          .MAASA..TTMLLKYPTTVCGIPN.....SSSNNSTDPS....NNIVQIPQTTTT...NSPL
Q9LKN0|Atriplex_hortensis                                  .MAASA..TTMLLKYPTTVCGIPN.....SSANNSTDPS....NNIVQIPQTTTT...NSPL
AAL92561|Atriplex_prostrata                                .MAASA..TTMLLKYPTTVCGIPN.....SSSNNSTDPS....NNIVQIPQTNTT...KSPL
O04121|Spinacia_oleracea__spinach_                         MMAASASATTMLLKYPTTVCGIPN.....PSSNNNNDPS....NNIVSIPQNTTN.....PT
ABG34274|Ophiopogon_japonicus                              .MAASASATTMLLKYPTTVCGIPN.....PSSNNNNDPS....NNIVSIPQNTTN.....PT
AEW31326|Haloxylon_persicum                                .MAGAASATTMLLKYPATLCSNS......GVSNNNNESSLSRDNNNLSIPQTNNNNNNKNPM
ACX47904|Haloxylon_ammodendron                             .MAGAASATTMLLKYPATLCSNS......GVSNNNNESSLSRDNNNLSIPQTNNNNNNNNPM
AJF98574|Salicornia_bigelovii                              .MAAAASATTMLLKYPS.LCS.LPNSSSS.PSNNNNEC..SRD...LNIPQTNT......PL
AAV91779|Salicornia_europaea                               .MAAAASATTMLLKYPS.LCSLPNSSSSS.SPSNNNEC..SRD...LNIPQTNT.....PPL
AFW04225|Suaeda_maritima                                   .MAASASATTMLLKYPT.ICG...........VPNNES..SSCSPKDNHLNVSQQQNNNNPL
AAM43920|Suaeda_liaotungensis                              .MAASASATTMLLKYPT.ICG...........VPNNES..SSCSPKDNHLNVSQQQNNNNPL
XP_010682183|Beta_vulgaris_subsp._vulgaris__sugar          ...MAASATTMLLKYPT.LCAMPNSSSSS....NNNDLPTSIPLNNNNNLLS..NKNKILQT
AHYPO_002206-RA|Amaranthus_hypochondriacus_439             ....MASSASMLINYPTTFCGVRNSS......NPNND......QFSDQ.INIPSSLNNNKI.
BAF93187|Amaranthus_tricolor                               ....MASSASMLINYPTTFCGVRNSS......NPNND......QFSDQ.INIPSSLNNNINI
XP_012828802|Erythranthe_guttata__spotted_monkey_          ......MAATMVK.............................LAFPHHFFPQIQEPN.FRLI
Migut.J00779.1|Mimulus_guttatus                            ......MAATMVK.............................LAFPHHFFPQIQEPN.FRLI
EYU18199|Erythranthe_guttata__spotted_monkey_flow          ......MAATTVK.............................LAFPNH..........FPLI
KQ992415.1|Dorcoceras_hygrometricum_corrected              ......MAAT....MMIQKLPTF.......HQSAQILPKPRTHFEFVKKRINKSQSNEIFVR
XP_011094129|Sesamum_indicum__sesame_                      ....MAMAAT....MMLPKLASFNH.....CPEIRNPYF..RFSRKNPRRCLTNNGNSFPVK
XP_015080536|Solanum_pennellii__Lycopersicon_penn          .............MVVLQKFTSF.......HQ.................FNKHKL.......
XP_004242785|Solanum_lycopersicum__Lycopersicon_e          .............MVVLQKFTSF.......HQ.................FNKHKS.......
NP_001275051|Solanum_tuberosum__potato_                    .............MVVLQRFTSF.......HQ.................FNKHKL.......
XP_016556147|Capsicum_annuum                               .............MEVLHKLTSF.......HQ.................FKNPKLCTSL..T
ACR15118|Lycium_barbarum                                   .............MALLQQLSSF.......HQ.................FKKPKLCCPL..N
XP_009757252|Nicotiana_sylvestris__wood_tobacco_           .............MAVLQKLSSFNHFSRQQHQ.............KLSQFKNPKLC......
XP_016460838|Nicotiana_tabacum_(common_tobacco)            .............MAVLQKLSSFNHFSRQQHQ.............KLSQFKNPKLC......
OIT26339|Nicotiana_attenuata                               .............MAVLQKLSSFNHFSRQQHQ.................FKKPKLCCPL...
XP_009601434|Nicotiana_tomentosiformis                     .............MAVLQKLSSFNHFPRQQSQ.................FKKPKLCCPL...
XP_016508540|Nicotiana_tabacum_(common_tobacco)            .............MAVLQKLSSFNHFPRQQSQ.................FKKPKLCCPL...
XP_015081828|Solanum_pennellii__Lycopersicon_penn          .............MEILQNLSSFNQ.....................................
XP_004243034|Solanum_lycopersicum__Lycopersicon_e          .............MAMVQNLSSFNQ.....................................
CDP08949|Coffea_canephora                                  .............MIQRSITSSFLVEIKNPNN........KFPLHYLYKIGPRSFCCN....
XP_019182381|Ipomoea_nil__Japanese_morning_glory_          ................................................MASGCIAATIPFSV
XP_010433050|Camelina_sativa__false_flax_                  ..........................................MTTMMMIRSTANLTEYLTPT
XP_010436340|Camelina_sativa_false_flax_corr               ..........................................MTTMMMIRSTANLTEYLTPT
XP_010438247|Camelina_sativa__false_flax_                  ..........................................MTTMMMIRSTATLKEYLTPT
XP_010447794|Camelina_sativa__false_flax_                  ..........................................MTTMMMIRSTATMTEYLTPT
XP_006285614|Capsella_rubella                              .............................................MSMIRSTATMSELLTPT
Cagra.0268s0015.1|Capsella_grandiflora                     .............................................MTMIRSTATMTELLTPT
Bostr.7867s0836.1|Boechera_stricta                         ............................................MMMMTTLKATMPEFLKPK
NP_194718|Arabidopsis_thaliana__thale_cress_               ..............................................MMTTLTATVPEFLPPS
XP_002869406|Arabidopsis_lyrata_subsp._lyrata              ..............................................MVTTLRASVPEFPSPS
Araha.6052s0002.1|Arabidopsis_halleri                      .............................................MMMTTLTSTVPEFLTPT
XP_006412796|Eutrema_salsugineum                           ........................................MVTITATVWEFPTPRSNPTSKY
KFK29548|Arabis_alpina__gray_rockcress_                    .................................................MVTTATVPEFLTP
KFK40841|Arabis_alpina__gray_rockcress_                    ..............................................................
XP_018481725|Raphanus_sativus__radish_                     .................MKQSMAVMTMM.....ATITPEFLSSTLISTTTNGYLKTRFDFGF
XP_018435688|Raphanus_sativus__radish_                     .................MKQSMAVMTMM.....ATITPEFLSSTLISTTTNGYLKTRFDFGF
XP_013705395|Brassica_napus__rape_                         ..................................MMTMLATITPPSSTLKSTTTGYLKLHLK
XP_013596413|Brassica_oleracea_var._oleracea               ..................................MMTMLATITPQSSTLKSTTTGYLNLHLK
XP_009137872|Brassica_rapa__field_mustard_                 .................................MMTMLATI.......KSTTTGYLKLHLKF
XP_013738248|Brassica_napus__rape_                         .................................MMTMLATITSPSSTLKSTTTGYLKLHHKF
XP_010541096|Tarenaya_hassleriana                          ..........................MA.....AITTMLPTAEIPEIFTPVLRVTGKFCDSG
Kaladp0809s0111.1|Kalanchoe_fedtschenkoi                   ..MPLISSFSSSSTSLHHHSLPTHHHHS.....LRLHHQTTRFSPNHPSSSLSLFTPPGRHP
Kalax.0333s0026.1|Kalanchoe_laxiflora_corr_462aa           ..MPLISSSSSSSTPLHHHRLPTHHHHS.....LRLHHQTTQFSPDHPSSSLSLFTPPGRHP
Kalax.0012s0114.1|Kalanchoe_laxiflora_corr_462aa           ..MPLISSSSSSTPLHHHHRLPTHHHHS.....LRLHHQTTQFSPNHPSSSLSLFNPPGRHP
XP_010667950|Beta_vulgaris_subsp._vulgaris__sugar          ...............................................MSAAKFNRLTHHVTF
AH021158-RA|Amaranthus_hypochondriacus_421aa-corr          ...............................................MAAAIQNLFNLPFGN
XP_021866412|426|Spinacia_oleracea_(spinach)               ............................................MSITHSITQNPLTNHVTL
BAG74777|Hordeum_vulgare_subsp._vulgare__domestic          ......................................................MATAQFRP
AKZ66518|Triticum_monococcum_subsp._aegilopoides           ..............................................................
FAOM01578495|Triticum_aestivum_UpdateAMA02065              ......................................................MATAQFLA
EMT07994|Aegilops_tauschii_                                ..............................................................
ACZ92178|Triticum_turgidum_subsp._durum__durum_wh          ..............................................................
KD266899|Triticum_urartu_UpdateEMS46986                    ..............................................................
ABV64740|Leymus_chinensis                                  ..............................................................
Brast07G193900.1|Brachypodium_stacei                       .......................................................MAIAQFL
XP_003563491|Brachypodium_distachyon__stiff_brome          .......................................................MAIAQVL
XP_006657294|Oryza_brachyantha__malo_sina_                 ..............................................................
XP_015643170|Oryza_sativa_Japonica_Group__Japanes          .........................................................MAIAQ
EEC81252|Oryza_sativa_Indica_Group__long-grained_          .........................................................MAIAQ
XP_002437525|Sorghum_bicolor__sorghum_                     ..........................................................MATG
NP_001105926|Zea_mays                                      ..........................................................MATG
Pavir.Db00260.1|Panicum_virgatum_CORR_316f                 ..............................................................
LWDX02005496.1|Dichanthelium_oligosanthes_update_          ...................................................MSTARSLAALS
Sevir.4G257300.1|Setaria_viridis                           ...................................................MAAARSLAALS
XP_004966128|Setaria_italica__foxtail_millet_              ...................................................MAAARSLAALS
Oropetium_20150105_16264A|Oropetium_thomaeum               ..................................................MATTTLSLAAFS
Pahal.D00004.1|Panicum_hallii                              ..................................................MATARSLAALLS
Pavir.J39646.1|Panicum_virgatum_Corr_363f                  ..................................................MATARSLAALPS
OAY65202|Ananas_comosus__pineapple_                        ....................................MAIGET.LAALSSSKTLILKPSPSPT
XP_009410214|Musa_acuminata_subsp._malaccensis__w          ....................................MAIVVAKIGAVLSAKTLALER.....
XP_010927268|Elaeis_guineensis__African_oil_palm_          ....................................MAIGET.LAALCSSKTLI.....PRS
XP_008801514|Phoenix_dactylifera__date_palm_               ....................................MAIGQA.LAALCSSKTLI.....PRS
KMZ72255|Zostera_marina                                    ........................................................MEIPLV
JAT51600|Anthurium_amnicola                                ..............................................................
Spipo5G0003400|Spirodela_polyrhiza                         ..............................................................
XP_006845775|Amborella_trichopoda                          .............................................MEVLRSSILGSQQLFPP
AFP19450|Camellia_sinensis                                 .................................MAMTLVKPNSFQLLESKKPRNQNHPFFNS
XP_017254907|Daucus_carota_subsp._sativus                  .....................................................MTTMMIVKP
ABX57826|Chrysanthemum_lavandulifolium                     ..............................................................
LEKV01000036.1|Cynara_cardunculus_update_KVI11819          ................................................MATMMMMMKPIGFQ
XP_008443631|Cucumis_melo__muskmelon_                      ...................................................MATLTKHIQIH
XP_004139149|Cucumis_sativus__cucumber_                    ...................................................MAMLTKHIQVH
XP_008351406|Malus_domestica__apple_                       ......................................................MAMTVATF
XP_008391562|Malus_domestica__apple_                       ......................................................MAMTVATF
XP_009343851|Pyrus_x_bretschneideri__Chinese_whit          ......................................................MAMTVATF
AER10510|Pyrus_betulifolia_corr_405aa                      ......................................................MAMTVATF
XP_008223853|Prunus_mume__Japanese_apricot_                .................................................MEMAMTVMNVATL
ONI27175|Prunus_persica__peach_                            .................................................MEMAMTVMNAATL
```

XP_004301419|Fragaria_vesca_subsp._vesca	....MTLTMSLASL
XP_015889685|Ziziphus_jujuba__common_jujube_	....MAMTMMLKP.
NW_010360102.1|Morus_notabilis_corrected	....MATAMSMSTT
Lus10032689|Linum_usitatissimum	....................................MAAVMASPETIKITVQPTLNRKAGNA
Lus10008571|Linum_usitatissimum_corrected	....................................MAAVMGSPETIKITVQPTLNRKTGNA
XP_011020077|Populus_euphratica__Euphrates_poplar	.....................................MTMITATTM......AVLLKPMTTA
XP_002308100|Populus_trichocarpa__Populus_balsami	.....................................MTMITATTM......AVLLKPMITA
SapurV1A.0198s0060.1|Salix_purpurea	.....................................MTMTTATTM......AVLLKPMISA
OAY46667|Manihot_esculenta__cassava_	.....................................MAIATALIP.............FKL
XP_002518256|Ricinus_communis__castor_bean_	.....................................MTIITVAAMITLTPI..........
XP_012074438|Jatropha_curcas	.....................................MSMTTAISMISLRPI..........
XP_010046909|Eucalyptus_grandis	.....................................MSMA..TTMIAIKPLFSPLTHHRSS
ABS71853|Eucalyptus_camaldulensis_Murray_red_gum_	.....................................MSMA..TTMIAIKPLFSPLTHHRSS
XP_006474292|Citrus_sinensis__sweet_orange_	....MAMTMSSTA
XP_006453220|Citrus_clementina_reviewed	....MAMTMSSTA
XP_017612508|Gossypium_arboreum	.............MHSNGSETRQLETELRGRNIHNLVSHRVETEMTLLLKPI..TRLSLFQT
XP_016686114|Gossypium_hirsutum__cotton_	....MTLLLKPI..TRLSLFQT
XP_012459381|Gossypium_raimondii	....MTLLLKPI..TRLSLFQT
XP_016719040|Gossypium_hirsutum__cotton_	....
EOY05290|Theobroma_cacao__cacao_	....MTLLLKPI..SEVFLFQS
AWWV01015497-AWWV01004954_Corchorus_capsularis_39	....MSYVFHS
OMO51681|Corchorus_olitorius	....
GAV59711|Cephalotus_follicularis	....MMMTMLMKQM
XP_018807260|Juglans_regia__English_walnut_	....MAMILKPIGTHLILRRPKYT
XP_019078540|Vitis_vinifera__wine_grape_	...........................MKGVDTQSARKRAQNLSDKGSSINMAIIFNVPLSS
Aqcoe7G261800.1|Aquilegia_coerulea	....MALLLKPISLTLFPFVKFKKTI
XP_010276721|Nelumbo_nucifera__sacred_lotus_	....MAM.LKPISFTISS......KR
XP_016201893|Arachis_ipaensis	....MHMQMALTMQLTPF.IP
XP_015973237|Arachis_duranensis	....MHVQMAVTMQLTPF.IP
Tp57577_TGAC_v2_mRNA5577|Trifolium_pratense	..............................MGTKGFRPTIQFRVRPRTQMAMIMQVTPF.IP
GAU38011|Trifolium_subterraneum	....MAMIMKVTPF.IP
XP_003610028|Medicago_truncatula__barrel_medic_	....MAMNMQVTPFNIP
XP_004507911|Cicer_arietinum__chickpea_	....MAMIMQMTPF.IP
XP_019463425|Lupinus_angustifolius__narrow-leaved	....MAMQLKPF.TL
KYP52554|Cajanus_cajan__pigeon_pea_	....MQMTTSLQLTPF.VS
XP_003549280|Glycine_max__soybean_	....MQMQMAMNIQLTPF.IS
XP_007134857|Phaseolus_vulgaris_CHR1_corr_208aaf	....
XP_007154695|Phaseolus_vulgaris_CORR	....MQMAMST..QLIPF.TS
XP_017410379|Vigna_angularis__adzuki_bean_	....MKMAMIM..QLIPF.IS
XP_014508581|Vigna_radiata_var._radiata__mung_bea	....MKMATIT..QLIPF.IS
AUSU01000986.1|Genlisea_aurea_corrected-f	....
OAE24413|CORR_Marchantia_polymorpha	....MASGGGMRLVRSTLQSLLSGR
Sphfalx0064s0059.1|Sphagnum	...................................MWRQAFKNNDALWKRVSCYAPRFELAD
XP_001752587|Physcomitrella_patens	....MSGTIWGGEN
XP_002508933|Micromonas_sp._RCC299	....
XP_003061830|Micromonas_pusilla_CCMP1545	....
XP_005645368|Coccomyxa	....
ADIC01002027.1|Chlorella_variabilis_corrected_pre	....
XP_013903967|Monoraphidium_neglectum_rev	....
XP_005644270|Coccomyxa_subellipsoidea_C-169	....
NC_024001.1|Bathycoccus_prasinos_corrected	....
EWM23896|Nannochloropsis_gaditana	......................MPIAVTFRKHHKLLAIMKPPTVLVKARCLFRYRSPMLQLL
XP_002738379|Saccoglossus_kowalevskii	....MPSRR
XP_006820984|Saccoglossus_kowalevskii	....
XP_002737044|Saccoglossus_kowalevskii	....
XP_002597329|Branchiostoma_floridae__Florida_lanc	....
XP_019618440|Branchiostoma_belcheri__Belcher's_la	....
XP_019644608|Branchiostoma_belcheri__Belcher's_la	................................MASLITSLGAARVRIFRPFTAVNRCFTTAD
XP_019644788|Branchiostoma_belcheri__Belcher's_la	................................MASLITSLGTARVRIFRPFTAVNRCFTTAD
XP_002610312|Branchiostoma_floridae__Florida_lanc	....
XP_002599795|Branchiostoma_floridae__Florida_lanc	....
XP_019633636|Branchiostoma_belcheri__Belcher's_la	....
XP_004333794|Acanthamoeba_castellanii_str._Neff	.......................MRSVVVARHETRSRATSLLDRASSGLWGSQGGLFTHHAK
GAQ84770|Klebsormidium_flaccidum	..........MAVSQSLLQSRAMSSALASISPSLASSKHRFASSAFVGEGLSHAKLGKDALP

	50 60 70 80 90
BAC77698|Atriplex_nummularia	LKFRT.TNKPI.NAVAA.PAPPS.VTTTTTSTP..PSIQSLVKDFDP....LVPAEDALTPP
AGC13074|Atriplex_semibaccata	LKFRT.TNKPI.NAVAA.PAPPS.VTTTTTTTP..PSIQSLVKDFDP....LVPAEDALTPP
AHH24260|Atriplex_amnicola	LKFRT.PNKPI.NAVAA.PASPS.VTTTKTTTP..PSIQSLVKDFDP....LVPAEDALTPP
AFG28558|Atriplex_canescens__four-winged_saltbush	LKFRT.PNKPI.NAVAA.PAFPS.VTTATTTTP..PSIQSLVKDFDP....LVPAEDALTPP
Q9LKN0|Atriplex_hortensis	LKFRT.PNKPV.NAVAA.PAFPS.VTTTTTTTP..SSIQSLVKDFDP....LVPAEDALTPP
AAL92561|Atriplex_prostrata	LKCRT.PNKPV.NAVAA.PAFPS.ATTITTTTP..PSIQSLVKDFDP....SIPAEDAFTPP
O04121|Spinacia_oleracea__spinach_	LKSRT.PNKITTNAVAA.PSFPS.L...TTTTP..SSIQSLVHEFDP....QIPPEDAHTPP
ABG34274|Ophiopogon_japonicus	LKSRT.PNKITTNAVAA.PSFPS.L...TTTTP..SSIQSLVHEFDP....QIPPEDAHTPP
AEW31326|Haloxylon_persicum	LKFRAQPNKIVTNAVAS.PVFPT.I.KTTTTTP..SSVQELVYKFDP....TIPAEDALTPP
ACX47904|Haloxylon_ammodendron	LKFRAQPNKIVTNAVASSPVFPA.I.KTTTTTP..SSVQELVYKFDP....TIPAEDALTPP
AJF98574|Salicornia_bigelovii	LKFRAQPNKLVANAVAS.PVFPC.L..TTTTTP..SSVKQLVHEFDP....KIPAEDALTPP
AAV91779|Salicornia_europaea	LKFRAQPNKLVANAVAS.PVFPS.L..TTTTTP.SSSVNQLVHEFDP....KIPAEDALTPP
AFW04225|Suaeda_maritima	LKFRTQPTKLVANAVAS.PVFPA.S.STTTSSPSSSSINQLVHEFDP....KIPPEDAFTPP
AAM43920|Suaeda_liaotungensis	LKFRTQPTKLVANAVAS.PVFPA.S.STTTSSPSSSSINQLVHEFDP....KIPPEDAFTPP
XP_010682183|Beta_vulgaris_subsp._vulgaris__sugar	PNINTSTNKIITKAVAS.PVFPT.L.KTTSNTP..SSIRSLVHEFDP....EIPPEDALTPP
AHYPO_002206-RA|Amaranthus_hypochondriacus_439	..TNNKTNKIIPKAVAS.PVIPS.INSNNITTTTTTNIKKIIHEFDP....KVAAQDAFTPP
BAF93187|Amaranthus_tricolor	SKITSKTNKIIPKAVAS.PVIPSSINSNNITTTT.PNIKRIIHEFDP....KVPAEDGFTPP
XP_012828802|Erythranthe_guttata__spotted_monkey_	TKEPIKPTNRAHLGKHYSHCRSKNGNFSTISENNNGKIRSLIEEFNP....EIPIEEAFTPP
Migut.J00779.1|Mimulus_guttatus	TKEPIKPTNRAHLGKHYSHCRSKNGNFSTISENNNGKIRSLIEEFNP....EIPIEEAFTPP
EYU18199|Erythranthe_guttata__spotted_monkey_flow	TKEPTKPGNRAHSSKNYSLCRSKNGNFSTSSEYKNGRVKKMIGEFNR....EIPIEEAFTPP
KQ992415.1|Dorcoceras_hygrometricum_corrected	RC..............GDRGDYLQRNCVEEINDVHGRARRLVQEFDP....RIPIEEAWTPP
XP_011094129|Sesamum_indicum__sesame_	SSSIQ........................GISGDERKVRRLVQEFNP....KIPIEEAVTPP
XP_015080536|Solanum_pennellii__Lycopersicon_penn	.SSIQFFTCPIKSKKHHL...........SFKISCNYSQKLVLEFNP....KIPIEEAITPP
XP_004242785|Solanum_lycopersicum__Lycopersicon_e	.SSIQFFTCPVKPKKHHF...........SFKISCNYNQKLVLEFNP....KIPIEEAITPP
NP_001275051|Solanum_tuberosum__potato_	.SSIQFFTCSVKPRKHHL...........SFKISCDSNQKLVLEFDP....KIPIEEAITPP
XP_016556147|Capsicum_annuum	PINTQISTCSLIPIKHNPSFQKVSC....SF...QDYNKKLVQEFVP....KIPIEEAITPP
ACR15118|Lycium_barbarum	PLKNQIFTYFIKPRKHLSSFQVSSSL...DYSNNHNYTKKLVQEFDP....NIPIEEAVTPP
XP_009757252|Nicotiana_sylvestris__wood_tobacco_	........CPIKHRIHHPSFQVSSSL.NTNYSNYHDYAQKLVQEFDP....KIPIEEAVTPP
XP_016460838|Nicotiana_tabacum_(common_tobacco)	........CPIKHRIHHPSFQVSSSL.NTNYSNYHDYAQKLVQEFDP....KIPIEEAVTPP
OIT26339|Nicotiana_attenuata	..KIQIFSCPIKHRIYHPSFQVSSSLINTNYSNHHDYN....QEFDP....KIPVEEAVTPP
XP_009601434|Nicotiana_tomentosiformis	PLKIQIFSCPIKHRIYHPSFQVSSSLINTNYSNHHDYAQKLVQEFDP....KIPIEEAVTPP
XP_016508540|Nicotiana_tabacum_(common_tobacco)	PLKIQIFSCPIKHRIYHPSFQVSSSLINTNYSNHHDYAQKLVQEFDP....KIPIEEAVTPP
XP_015081828|Solanum_pennellii__Lycopersicon_penn	......SIKITPLKNHIFTCSVKPKRHYHSFQISCNYNQKLVLEFDP....KIPIEEAITPP
XP_004243034|Solanum_lycopersicum__Lycopersicon_e	......SIKITPLKIHIFTCSVKPRRHYHSSQISCNYYRKLVLEFDP....KIPIEEAITPP
CDP08949|Coffea_canephora	.......FNLSKPKKH.....PIYKCSFTVSSSLDKEAQTLIQQFNP....QIPIEEALTPP
XP_019182381|Ipomoea_nil__Japanese_morning_glory_	HRREPSALSTPRYCRLQFTNGMHRLRLGVLPLSCGNASFSLTREFNP....DIPIEEAQTPP
XP_010433050|Camelina_sativa__false_flax_	LKSTKGNFSSHGGFGVSVSQFSRRRFLNPSRVFAVSDINKLVKEFDP....KIPLERASTPP
XP_010436340|Camelina_sativa_false_flax_corr	LKSTKGNFSSHGGFGVSVSQFSRRRFLNPSRVFAVSDINKLVKEFDP....KIPLERASTPP
XP_010438247|Camelina_sativa__false_flax_	LKSTKGNFSSHGEFGVSVSQFSRRRYLSPSRVFAVSDINKLVTEFDP....KIPLERASTPP
XP_010447794|Camelina_sativa__false_flax_	QKSTRGYFNSHGEFGVSVSQFSRRRFLSPSRVFAVSDINKLVMEFDP....KIPLERASTPP
XP_006285614|Capsella_rubella	LKSSRGYFNAHGKFGVSVSQFSRRRFLNPSRVFAVSDISKLVTEFDP....NIPLERASTPP
Cagra.0268s0015.1|Capsella_grandiflora	LKSTRGYFNAHGKFGVSVSQFSRRRFLNPSRVFAVSDISKLVTEFDP....NIPLERASTPP
Bostr.7867s0836.1|Boechera_stricta	LKSTRGYFNSHGEFGVSVSQFGWRRFLNTSRVFAVSDISKLVTEFDP....EIPLERASTPP
NP_194718|Arabidopsis_thaliana__thale_cress_	LKSTRGYFNSHSEFGVSISKFSRRRFHNPTRVFAVSDISKLVTEFDP....KIPLERASTPP
XP_002869406|Arabidopsis_lyrata_subsp._lyrata	LKTTRGYFNSHGDFGVSVSQFGRRRFLIPSRVFAVSDINKLVTEFDP....KIPLERASTPP
Araha.6052s0002.1|Arabidopsis_halleri	LKSTCRYFNFHGEVSVSVSQFGRRRFHNPSRVFAVSDINKHVTEFDP....KIPLERASTPP
XP_006412796|Eutrema_salsugineum	LSSHREFGVSVSQFRRRRFLYPTRDPSSSSIVFAVSEISKLVAEFDP....EIPLERASTPP
KFK29548|Arabis_alpina__gray_rockcress_	TTLKSTIRFINSHRQFFGRRFLDPIPNSSSRIFAVSEISKLVTEFDP....EIPLERASTPP
KFK40841|Arabis_alpina__gray_rockcress_	..................MSFEVSLPG.....FWIYGVDVVVFESD................
XP_018481725|Raphanus_sativus__radish_	SVSQFDRQRRRKCLLHPTLFCTTSSSSGRVSGAASSIISNLVEEFDP....EIPVERASTPP
XP_018435688|Raphanus_sativus__radish_	SVSQFDRQRRRKCLLHPTLFCTTSSSSGRVSGAASSIISNLVEEFDP....EIPVERASTPP
XP_013705395|Brassica_napus__rape_	FSVSVSQSARRRCLDQSLFCTRDPISSSSRVVAAASNTSNLVEEFDP....EIPVERALTPP
XP_013596413|Brassica_oleracea_var._oleracea	FSVSVSQSARRRCLDQSLFCTRDPISSSSRVVAAASNTSNLVEEFDP....EIPVERALTPP
XP_009137872|Brassica_rapa__field_mustard_	SVSVSQFVRGRRRIHQTLLCTRDPISSSSRVFAAASNTSNLVEEFDP....EIPVERALTPP
XP_013738248|Brassica_napus__rape_	SVSVSQFRHRRRRIHQTLLCTRDPISSSSRVFAAASNTSNLVEEFDP....EIPVERALTPP
XP_010541096|Tarenaya_hassleriana	GEIFSSGSRFRRRRSLRSILSCRRDTSSSRRIAAVSEVRKLIDEFDP....EIPVEWSATPP
Kaladp0809s0111.1|Kalanchoe_fedtschenkoi	AALDHHRQLFCIPVTTNTCFNPSSVISSDAEQVAKAAALSLVDEFDP....TIPIQTALTPP
Kalax.0333s0026.1|Kalanchoe_laxiflora_corr_462aa	AALDHHRRLFCIPITTNTCFDPSSVISSDAEQVAKAAALSLVDEFDP....TIPIQTALTPP
Kalax.0012s0114.1|Kalanchoe_laxiflora_corr_462aa	AALDHHRRLFCIPVTTKTCFNPSSVISSDAEQVAKAAALSLVDEFDP....TIPIQTALTPP
XP_010667950|Beta_vulgaris_subsp._vulgaris__sugar	HLFDNNLKRQRFPFNFPNYKIQTPIKLNICLSSSNQSTHKIVHEFDP....NIPIEEAVTPP
AH021158-RA|Amaranthus_hypochondriacus_421aa-corr	YPRRNINRIPLI.LPNPKFRVPIEVANGFSSSSLQSKTHKILHQFDP....NLPLEEAVTPP
XP_021866412|426|Spinacia_oleracea_(spinach)	QSFGNNFIPKIERFPNRIHQAPIKLTKCLSNSSSIQSTHKIAHEFDP....NIPIEEAQTPP
BAG74777|Hordeum_vulgare_subsp._vulgare__domestic	LSSSSSASAAARPRAFRAAPSRV....AAAASASGEPARRLAAEFDP....AVPLASAVTPP
AKZ66518|Triticum_monococcum_subsp._aegilopoides	....
FAOM01578495|Triticum_aestivum_UpdateAMA02065	LSSSSSASAAARPRACRAAPSRVCVAAAAAASSSGEPARRLAAEFDP....AIPLACAVTPP
EMT07994|Aegilops_tauschii_	....
ACZ92178|Triticum_turgidum_subsp._durum__durum_wh	....
KD266899|Triticum_urartu_UpdateEMS46986	......................................MATAQEFDP....AIPLASAVTPP
ABV64740|Leymus_chinensis	....
Brast07G193900.1|Brachypodium_stacei	ALSSSTFSFARTDRPKVRRAAPSRVAASEAGATVGEPARRLVAAFDP....AIPLASAVTPP
XP_003563491|Brachypodium_distachyon__stiff_brome	ALSSSTSSFVRTDRPKVRRAAPSRVAAAAAGATVGEPARRLVAAFDP....AIPLASAVTPP
XP_006657294|Oryza_brachyantha__malo_sina_	....FDP....AVPLASAVTPP
XP_015643170|Oryza_sativa_Japonica_Group__Japanes	SAAAVSSAARASRPRPTRAAPRRIAASASSVAPPEPAARRLVAAFDP....AVPLASAVTPP
EEC81252|Oryza_sativa_Indica_Group__long-grained_	SAAAVSSAARASRPRPTRAAPRRIAASASSVATPEPAARRLVAAFDP....AVPLASAVTPP
XP_002437525|Sorghum_bicolor__sorghum_	RSLAAVYSARASRTVPFRAGAR..PARAVAAAAAAAEHVRRLVAEFDP....AVPLDSAITPP
NP_001105926|Zea_mays	RSLAGVYSARATRPMPLRAGARAPCAGAAAAEPAAEHVRRLVAEFDP....AVPLDSAVTPP
Pavir.Db00260.1|Panicum_virgatum_CORR_316f	....
LWDX02005496.1|Dichanthelium_oligosanthes_update_	SARATRHRCRRAGGAWPPRAVAAGRATAEPTPA.PEHVRRLVAEFDP....AVPLASAVTPP
Sevir.4G257300.1|Setaria_viridis	SARATRLGPRRAG.AWPPR...VSSAAARAVAAEPEHARRLVAEFDP....AVPLDAAVTPP
XP_004966128|Setaria_italica__foxtail_millet_	SARATRPGPRRAG.AWPPR...VSAAVARAVAAEPEHARRLVAEFDP....AVPLDAAVTPP
Oropetium_20150105_16264A|Oropetium_thomaeum	SS...SSLNSRTGRPRTPR........AAFGAAERERALRLVAEFDP....AVPLASAVTPP
Pahal.D00004.1|Panicum_hallii	.ARATRPGPRRAGGAWPPR.......FAARAAAEPEHARRLVAEFDP....AVPLEAAVTPP
Pavir.J39646.1|Panicum_virgatum_Corr_363f	.AGATRPGSRLAGRARPPR......VAAARAAAESDLARRLVAEFDP....AVPLEAAVTPP
OAY65202|Ananas_comosus__pineapple_	TSSSSTRSLRHWIPCFSRHHPSLATSSSAAAAAAAEARRSMVEAFDP....TIPLAEALTPP
XP_009410214|Musa_acuminata_subsp._malaccensis__w	FLSSPSYSSSTRGVGL..RVACRSSPSPSTETRISRAQRRMVEAFNP....DIPLAEAITPP
XP_010927268|Elaeis_guineensis__African_oil_palm_	VPSSSSASARPRGVRLACSLRFPSSLAPEIGIFDTQALRRMVEAFDS....KIPLAEALTPP
XP_008801514|Phoenix_dactylifera__date_palm_	FLSSSSASARPRGVRLACSSWSLPSPSPEIGISDTHALRRTVEAFDP....RIPLAEALTPP
KMZ72255|Zostera_marina	RHCLTTRHLNHHTLLPKTRLPGLKFREPINSRCHSTSSRSVVDAFDP....TIPLSRAQTPP
JAT51600|Anthurium_amnicola	.........................PPPPPPPLADSVHRRMVEGFDP....SVPLSQAHTPP
Spipo5G0003400|Spirodela_polyrhiza	....
XP_006845775|Amborella_trichopoda	LKLYSFKTRFSLSKLIPFSSSLSSSSSLSSLREDRGKFRSLIESFDP....KTPLEEAFTPP
AFP19450|Camellia_sinensis	SFGQRRSINPIFKSSYNPSFSSSYSSPSSSSLETLDEARRLVYQFDP....KIPLEEALTPP
XP_017254907|Daucus_carota_subsp._sativus	LHLFQTSNTTAATKTSPFSQSPRSLSKISCVSSPQTPTRLLVDEFNP....KIPIEKALTPP
ABX57826|Chrysanthemum_lavandulifolium	....
LEKV01000036.1|Cynara_cardunculus_update_KVI11819	FSDLKKLRNPYPNSPLIRRSH...EPNHGLLQMSQDLDRSLVHEFNP....SIPIQEALTPP
XP_008443631|Cucumis_melo__muskmelon_	FFQPPSISFNFHFCNHRSPSRISASLSFRSPDSRFIEARKLVDDFDP....EIPLEKALTPP
XP_004139149|Cucumis_sativus__cucumber_	FFQLPSTSFNFHSCNHRSPPRISAALSFRNPDSRLIEARKLVDDFDP....QIPLEKALTPP
XP_008351406|Malus_domestica__apple_	QPKTLRPKSPYLLPNLPRRVCNFKAFSLHNPQ.....LPSLVGQFEP....TIPIERAGTPP
XP_008391562|Malus_domestica__apple_	QPKTLRPKSPYLLPNLPRRVCNFKAFSLHNPQ.....LPSLVGQFEP....TIPIERAGTPP
XP_009343851|Pyrus_x_bretschneideri__Chinese_whit	QPKTLRPKSPYLLPNLPRKVCNFKAFSLHNPQ.....LPSLVGQFEP....TIPIERAGTPP
AER10510|Pyrus_betulifolia_corr_405aa	QPKTLRPKSPYLLPNLPRKVCNFKAFSLHNPQ.....LPSLVGQFEP....TIPIERAGTPP
XP_008223853|Prunus_mume__Japanese_apricot_	QPKPLRPKSPYLLPN.PRRVFNFKASSLHNPQVSSIGAPNLVGQFDP....TIPIERAVTPP
ONI27175|Prunus_persica__peach_	QPKPLRPKGPYLLPN.PRRVINFKASSLHNPQVSSIGAPTLVGQFDP....TIPIERAVTPP

**XP_004301419|Fragaria_vesca_subsp._vesca**	KPKPIRPKSPYL.PQTLNSSRIFKSLSLPNPSV.GVGAPTLVDQFDP....TIPIERAVTPP
**XP_015889685|Ziziphus_jujuba__common_jujube_**	..TSPVFFHQTKNPN..RRRS.FKAFSLHNSETSSLPPSTLVHQFDP....RIPIEEALTAP
**NW_010360102.1|Morus_notabilis_corrected**	..TATATTAKTTSLMSVSLR...RSWSLKAFSVKAASSRCSTSEFDP....KIPIEKAPTPP
**Lus10032689|Linum_usitatissimum**	VFHWNPIIRNTSQQLRRRRRPIIVGSSSAGQSWKRSHCENLVHEFDP....TIPIEKALTPP
**Lus10008571|Linum_usitatissimum_corrected**	VFHWNPIIRNTSQQLRRRRRPIIVSSSSAGQSWKRSHCENLVHEFDP....TIPIEKALTAP
**XP_011020077|Populus_euphratica__Euphrates_poplar**	HRLLLFQTQKHTSIIAKQQKQHGSLLVASSVRQSDNYCQNLVDEFDP....NIPIEKALTPP
**XP_002308100|Populus_trichocarpa__Populus_balsami**	HRLLLFQTQKHTSIIAKQQKQHGSLLVASSVRQSDNYCQNLVDEFDP....NIPIEKALTPP
**SapurV1A.0198s0060.1|Salix_purpurea**	QRLLLFQTQKHTSIIAQQPKQHGSLLVASSVRHSDNYCQNLVDEFDP....NIPIEKALTPP
**OAY46667|Manihot_esculenta__cassava_**	INGRRFQSKNHTPILAQRLHFKVSCCVL.NPISRDSHYQKLVDEFDP....RIPIEKALTPP
**XP_002518256|Ricinus_communis__castor_bean_**	....VSRSLQIKNQSSVTAQHRSFHSLPKNSHSLQTHFQNLVNKFDP....HIPVEEAFTPP
**XP_012074438|Jatropha_curcas**	....SSRVHQIQQHNPIIAQHR...PSLTVSFSLQTHCQKLVDEFDP....HIPIEKASTPP
**XP_010046909|Eucalyptus_grandis**	RNARLHHHTTRMPLSLNRVATRLATSLASAAAAPPSSAESLVDDFDP....ELPIEEALTPP
**ABS71853|Eucalyptus_camaldulensis_Murray_red_gum_**	RNARLHHHTTRMPLSLNRVATRLATSLASAAAAPPSSAESLVDDFDP....ELPIEEALTPP
**XP_006474292|Citrus_sinensis__sweet_orange_**	MIMKIPNHNTFKSHQLLNCVKKRSCSSSSCYSENLVAAQKLVYEFNP....QIPIEKALTPP
**XP_006453220|Citrus_clementina_reviewed**	MIMKIPNHNTFKSHQLLNC..............NLVAAQKLVYEFNP....QIPIEKALTPP
**XP_017612508|Gossypium_arboreum**	HFQIINNNNPRARARARARAISVSCS.......WSHEAHKIVREFDP....KIPIEKAITPP
**XP_016686114|Gossypium_hirsutum__cotton_**	HFQIINNNNPRA....RARAISVSCS.......WSHEAHKIVREFDP....KIPIEKAITPP
**XP_012459381|Gossypium_raimondii**	HFQINNNNNP......RARETSVSCS.......WSHGAHKIVREFDP....KIPIEKAITPP
**XP_016719040|Gossypium_hirsutum__cotton_**	................RARATSVSCS.......WSHGAHKIVREFDP....KIPIEKAITPP
**EOY05290|Theobroma_cacao__cacao_**	HFQH.........PRLIANFSSVSCCS....DRESYKAQTMVREFDP....KIPIEKALTPP
**AWWV01015497-AWWV01004954_Corchorus_capsularis_39**	QNQI.........QRLMAKFSSISCC.......WSRQVESMVRQFDP....KIPIEEAVTPP
**OMO51681|Corchorus_olitorius**	....MVREFDP....KIPIEEAVTPP
**GAV59711|Cephalotus_follicularis**	VPKCKSQNHNQNQNQNENGKVNRILNSCYSVDNPKEKAQNLVYQFDP....KIPIEKATTPP
**XP_018807260|Juglans_regia__English_walnut_**	NPNIHSCKHKHHHRPLTAAFCSIHGPNSSSEDISLHQAQKMVNQFDP....KVPIEEAVTPP
**XP_019078540|Vitis_vinifera__wine_grape_**	SSSSRSKFNSRNSHILFQKRCPFPNRTIVNSSSAAGKAPTLLHKFNP....RIPVEQALTPP
**Aqcoe7G261800.1|Aquilegia_coerulea**	ILEKTTTRRRTSNSTQLTHQFFFKQISNCFDDTSSKQISRMHDEFNP....EIRIEEAETPP
**XP_010276721|Nelumbo_nucifera__sacred_lotus_**	TGRRNPDDPSQGGLFKLVCNSYSCNKSNLPSSEASVGALRLVHEFNP....KIPLEEAVTPP
**XP_016201893|Arachis_ipaensis**	KLGRQRHSHNLNFHHKTASTSIIACCSVRNS....SEAEVLVSEFNP....KIPIEEAVTPP
**XP_015973237|Arachis_duranensis**	KLRRLRSSHNLNFHNKTAATSIIACCSARNS....SEAEVLVSEFNP....KIPIEEAVTPP
**Tp57577_TGAC_v2_mRNA5577|Trifolium_pratense**	NLQKG.HPQNLNFSNKH..SSKLICCSLNNS..DHVVSQKLAQHFNP....NIPIEKAVTPP
**GAU38011|Trifolium_subterraneum**	NLQKR.QPQNLNFPNKH..SSKLICCSLNSS..DHVVSQKLAQHFNP....NIPIEKAVTPP
**XP_003610028|Medicago_truncatula__barrel_medic_**	NLQKR.QPQNLNFPNKH..SSKPICCSLSSS..DMVVSQKLAQQFNP....NIPIEKAVTPP
**XP_004507911|Cicer_arietinum__chickpea_**	NLRKG.QTQNLNFPNKH..SSIRSCCSVGSS..DLVVSQRLVQQFNP....KIPVEEAVTPP
**XP_019463425|Lupinus_angustifolius__narrow-leaved**	YLGQS.QPRNLNFPNKH...KIITCCSVRDS.VSNTQNHKLVHQFNP....KIPIEEAVTPP
**KYP52554|Cajanus_cajan__pigeon_pea_**	NLRQR.QLLNPNFPKKH...ATFTCCTIRNSGHLVTETQSLVHQFNP....KIPIEEAVTPP
**XP_003549280|Glycine_max__soybean_**	NPRQG.QLLNLNFPNKH...STLTCCAIRNSDLKLSQTQRLVHHFNP....KTPIEEAVTPP
**XP_007134857|Phaseolus_vulgaris_CHR1_corr_208aaf**	....
**XP_007154695|Phaseolus_vulgaris_CORR**	TPRQG.QLINLNFPNKS...LTITRCVIRNSDVHTSETQRLVHHFNP....KTPIEEAVTPP
**XP_017410379|Vigna_angularis__adzuki_bean_**	TPRQG.QLMNLNFSNRS...SILSRCLIHNSDVQTSQTQRLVHHFNP....KTPIEEAITPP
**XP_014508581|Vigna_radiata_var._radiata__mung_bea**	TPRQG.QLVNLNFSNKS...SILSRCLIHNSDVQTSQTQRLIHHFNP....KTPIEEAITPP
**AUSU01000986.1|Genlisea_aurea_corrected-f**	....................................RAGRLVAEFDP....EIPIEEAVTPP
**OAE24413|CORR_Marchantia_polymorpha**	RPLGSLGVIPTVECSIWKRRNVNNSHPPGFVPNRWSSVEAEISRFDR....VIQIEEASTPP
**Sphfalx0064s0059.1|Sphagnum**	CKSLHVAVRREHSGSQSDLVSSSGHLTTDDDGVGDRPLRATVMAFQS....SKPIEEAVTPP
**XP_001752587|Physcomitrella_patens**	LTQIVESPLGGLSCRNRSSVGGDDDLTGLKTTPSSPRVERTLREFRR....HAPVEEASTPP
**XP_002508933|Micromonas_sp._RCC299**	....VASSATPP
**XP_003061830|Micromonas_pusilla_CCMP1545**	....ASTPP
**XP_005645368|Coccomyxa**	....QASTPP
**ADIC01002027.1|Chlorella_variabilis_corrected_pre**	......................MKPLAQIG.ITTSKLAIQCQAPPAP....AQPISRAATPP
**XP_013903967|Monoraphidium_neglectum_rev**	....MISAGLEPWADHARPIEEATTLP
**XP_005644270|Coccomyxa_subellipsoidea_C-169**	....MAYA.......PIEEANTPP
**NC_024001.1|Bathycoccus_prasinos_corrected**	..............MGFSTPKIFKEEEEEEEEREEDARTTQPHDFTPWTSFDKAIEDASPIP
**EWM23896|Nannochloropsis_gaditana**	SRTCRAGNSSSSSLPYSTGAKASTSPFERSLFQYKDYLKRELARFDP....DVPIELASTPP
**XP_002738379|Saccoglossus_kowalevskii**	LAVLKRATSSSSNL............RTGDTNDPSFDLRREVAKFDA....NIPVEAATTPP
**XP_006820984|Saccoglossus_kowalevskii**	.....MITEKLYNATTVNFLKASATVALRRYASWDVNPRLDVHTFQC....DLPVEEATTPP
**XP_002737044|Saccoglossus_kowalevskii**	.MFRICVYSLRISRVLQAQLARTRSTAAVCAELENGFVEKEVLKFDP....QLPVERAPTPP
**XP_002597329|Branchiostoma_floridae__Florida_lanc**	.MGRLFKVGCAFVRHRSSVLTPILQVVTKPLSTTSTSVTTEVHKFCL....ETPIDEATTPP
**XP_019618440|Branchiostoma_belcheri__Belcher's_la**	.MRGLYKVGCIFVKD..PTLTSILQVFTKPLSTTSSSVAAEVHKFCL....ETPIEEATTPP
**XP_019644608|Branchiostoma_belcheri__Belcher's_la**	RRFKTADRRFQTVDRRFKTADRRFQTVDRRFTKIDRAVADEVLRFSL....DAPVQEAFTPP
**XP_019644788|Branchiostoma_belcheri__Belcher's_la**	RRFK.......SVDRRFQTADRRFKTVDRRFTKIDPAVADEVLRFSL....DAPVHEALTPP
**XP_002610312|Branchiostoma_floridae__Florida_lanc**	....
**XP_002599795|Branchiostoma_floridae__Florida_lanc**	.......MFRKTVLVSCRLAGRRFRSSVVAPSTTDAYVAEEVQKFCT....ETPVERATTPP
**XP_019633636|Branchiostoma_belcheri__Belcher's_la**	.......MFRRTVLISFRFAERRYRSSAVAPSARDAYVAEEVQKFCT....ETPVERATTPP
**XP_004333794|Acanthamoeba_castellanii_str._Neff**	GGHLRTTASATTKARASSRLLSTAAAAATRTLNDDALFAREAHRWDP....QVPIERATTPP
**GAQ84770|Klebsormidium_flaccidum**	RHVEEGPARIQAVMESTEQQLRNLERALKAFPSASSDKPPVSSTWDP....KLPLDEGSCPP

```
                                                      100        110        120        130        140
BAC77698|Atriplex_nummularia                          SSWYTEPAFY.AHELDRIFY..KGWQVAGYSDQVKEANQYFTGTL.........GNVEYLV
AGC13074|Atriplex_semibaccata                         SSWYTEPAFY.AHELDRIFY..KGWQVAGYSDQVKEANQYFTGTL.........GNVEYLV
AHH24260|Atriplex_amnicola                            SSWYTEPAFY.AHELDRIFY..KGWQVAGYSDQVKEANQYFTGTL.........GNVEYLV
AFG28558|Atriplex_canescens__four-winged_saltbush     SSWYTEPAFY.AHELDRIFY..KGWQVAGYSDQVKEANQYFTGTL.........GNVEYLV
Q9LKN0|Atriplex_hortensis                             SSWYTEPAFY.AHELDRIFY..KGWQVAGYSDQVKEANQYFTGTL.........GNVEYLV
AAL92561|Atriplex_prostrata                           SSWYTEPAFY.AHELDRIFY..KGWQVAGYSDQVKEANQYFTGTL.........GNVEYLV
O04121|Spinacia_oleracea__spinach_                    SSWYTEPAFY.SHELERIFY..KGWQVAGISDQIKEPNQYFTGSL.........GNVEYLV
ABG34274|Ophiopogon_japonicus                         SSWYTEPAFY.SHELERIFY..KGWQVAGISDQIKEPNQYFTGSL.........GNVEYLV
AEW31326|Haloxylon_persicum                           STWYTEPAFY.SHELERIFY..KGWQIAGYSDQIKEKNQYFTGRL.........GNVEFLV
ACX47904|Haloxylon_ammodendron                        STWYTEPAFY.SHELERIFY..KGWQIAGYSDQVKEKNQYFTGRL.........GNVEFLV
AJF98574|Salicornia_bigelovii                         SSWYTEPAFY.SHELDRIFY..KGWQVAGISDQIKEKNQYFTGRL.........GNVEYVV
AAV91779|Salicornia_europaea                          SSWYTEPAFY.SHELDRIFY..KGWQVAGISDQIKEKNQYFTGRL.........GNVEYVV
AFW04225|Suaeda_maritima                              SSWYTEPAFY.SHELDRIFY..KGWQVAGISDQIKEKNQYFTGTL.........GNVEYVV
AAM43920|Suaeda_liaotungensis                         SSWYTEPAFY.SHELDRIFY..KGWQVAGISDQIKEKNQYFTGRL.........GNVEYVV
XP_010682183|Beta_vulgaris_subsp._vulgaris__sugar     STWYTEPAFY.SHELERIFY..KGWQVAGYSEQVKEKNQYFTGSL.........GNVEYLV
AHYPO_002206-RA|Amaranthus_hypochondriacus_439        STWYTEPSLY.SHELDRIFY..KGWQVAGYSDQIKEPNQYFTGSL.........GNVEYLV
BAF93187|Amaranthus_tricolor                          STWYTDPSLY.SHELDRIFY..KGWQVAGYSDQIKEPNQYFTGSL.........GNVEYLV
XP_012828802|Erythranthe_guttata__spotted_monkey_     SSWYTDPDFY.SHELDHIFY..TGWHPVGYTDQIKEPRDFFTGRL.........GSIEYVV
Migut.J00779.1|Mimulus_guttatus                       SSWYTDPDFY.SHELDHIFY..TGWHPVGYTDQIKEPRDFFTGRL.........GSIEYVV
EYU18199|Erythranthe_guttata__spotted_monkey_flow     SSWYTDSEFY.SHELTQIFY..RGWQPVGYTDQVKEPRDFFTGRL.........GSVEYVV
KQ992415.1|Dorcoceras_hygrometricum_corrected         SCWYTDPDFY.ALELQQVFR..KGWHPVGYTDQIRDPRDFFTGSL.........GSIEYVV
XP_011094129|Sesamum_indicum__sesame_                 SSWYTDPDFY.SLELDQVFY..RGWQAVGYTEQIKEPREFFTGRL.........GSIEYVV
XP_015080536|Solanum_pennellii__Lycopersicon_penn     SSWYTDSCFY.THELNQVFF..KGWQSVGYTEQIKEPRQYFTGRL.........GNVEYVV
XP_004242785|Solanum_lycopersicum__Lycopersicon_e     SSWYTDSCFY.THELNQVFF..KGWQSVGYTEQIKEPRQYFTGRL.........GNVEYVV
NP_001275051|Solanum_tuberosum__potato_               SSWYTDSCFY.THELNQVFF..KGWQAVGYTEQIKEPRQYFTGRL.........GNVEYVV
XP_016556147|Capsicum_annuum                          SSWYTDSSFY.SHELNHVLF..KGWQAVGYTEQIKEPRQYFTGRL.........GNVEYVL
ACR15118|Lycium_barbarum                              SSWYTDTSFY.THELNQVFF..KGWQVVGYSEQIKEPSQYFTGRL.........GNVEYVV
XP_009757252|Nicotiana_sylvestris__wood_tobacco_      SSWYTDPSFY.NQELNQVFF..KGWQAVGYTEQIKEPSQYFTGRL.........GNVEYVV
XP_016460838|Nicotiana_tabacum_(common_tobacco)       SSWYTDPSFY.NQELNQVFF..KGWQAVGYTEQIKEPSQYFTGRL.........GNVEYVV
OIT26339|Nicotiana_attenuata                          SSWYTDPSFY.NHELNQVFF..KGWQAVGYTEQIKEPRQYFTGRL.........GNVEYVV
XP_009601434|Nicotiana_tomentosiformis                SSWYTDPSFY.NHELNQVFF..KGWQAVGYTEQIKEPSQYFTGRL.........GNVEYVV
XP_016508540|Nicotiana_tabacum_(common_tobacco)       SSWYTDPSFY.NHELNQVFF..KGWQAVGYTEQIKEPSQYFTGRL.........GNVEYVV
XP_015081828|Solanum_pennellii__Lycopersicon_penn     SSWYTDSCFY.THELNQVFF..KGWQSVGYAEQIKDPGQYCTGRL.........GNVEYVV
XP_004243034|Solanum_lycopersicum__Lycopersicon_e     SSWYTDSCFH.THELNQVFF..KGWQSVGYAGQIKDPGQYFTGRL.........GNVEYVV
CDP08949|Coffea_canephora                             SSWYTDPSFH.SLELQQVFY..RGWQAVGYTEQIKDPHQYFTGRL.........GNVEYVV
XP_019182381|Ipomoea_nil__Japanese_morning_glory_     TSWYTDPSFY.SLELSRVFY..RGWQAVGYTEQVNEPRQFFTGRL.........GNIEYVV
XP_010433050|Camelina_sativa__false_flax_             SSWYTDPQFY.SFELDRVFY..GGWQAVGYSDQIKESRDFFTGRL.........GGVEYVV
XP_010436340|Camelina_sativa_false_flax_corr          SSWYTDPQFY.SFELDRVFY..GGWQAVGYSDQIKESRDFFTGRL.........GGVEYVV
XP_010438247|Camelina_sativa__false_flax_             SSWYTDPQFY.SYELDRVFY..GGWQAVGYSDQIKESRDFFTGRL.........GGVEYVV
XP_010447794|Camelina_sativa__false_flax_             SSWYTDPQFY.SFELDRVFY..GGWQAVGYSDQIKESRDFFTGRL.........GDVEYVV
XP_006285614|Capsella_rubella                         SSWYTDPQFY.SFELDRVFY..GGWQAVGYSDQIKESRDFFTGKL.........GDVEFVV
Cagra.0268s0015.1|Capsella_grandiflora                SSWYTDPQFY.SFELDRVFY..GGWQAVGHSDQIKESRDFFTGKL.........GDVEFVV
Bostr.7867s0836.1|Boechera_stricta                    SLWYTDPQFY.SFEIDRVFY..GGWQAVGYSDQIKESRDFFTGRL.........GDVEFVV
NP_194718|Arabidopsis_thaliana__thale_cress_          SSWYTDPQFY.SFELDRVFY..GGWQAVGYSDQIKESRDFFTGRL.........GDVDFVV
XP_002869406|Arabidopsis_lyrata_subsp._lyrata         SSWYTDPQFY.SFELDRVFY..GGWQAVGYSDQIKESRDFFTGRL.........GDVEYVV
Araha.6052s0002.1|Arabidopsis_halleri                 SSWYTDPKFY.SFELDRVFY..GGWQAVGYSDQIKESRDFFTGRL.........GDVEYVV
XP_006412796|Eutrema_salsugineum                      SSWYTDPQFY.RFELDRVFY..GAWQAVGYSDQIKENRDFFTGRL.........GEVEFVV
KFK29548|Arabis_alpina__gray_rockcress_               SSWYTDPRFY.SFELDRVFY..GGWQVVGFSEQIKDNRDFFTGRL.........GDVEFVV
KFK40841|Arabis_alpina__gray_rockcress_               .......GFY.SFEPDRVFY..GGWQAVGFSEQIKDNRDFFTGRL.........GDVEFVV
XP_018481725|Raphanus_sativus__radish_                SSWYTDHQFH.RYELHRVFY.GGGWQAVGYSDQIKDNRHFFTGRL.........GEVEFVV
XP_018435688|Raphanus_sativus__radish_                SSWYTDHQFH.RYELDRVFY.GGGWQAVGYSDQIKDNRHFFTGRL.........GEVEFVV
XP_013705395|Brassica_napus__rape_                    SSWYTDHQFH.RGELDRVFY..GGWQVVGCSDHIKDNRDFFTGRL.........GEVEFVV
XP_013596413|Brassica_oleracea_var._oleracea          SSWYTDHQFH.HDELDRVFY..GGWQVVGCSNQIKDNRDFFTGRL.........GEVEFVV
XP_009137872|Brassica_rapa__field_mustard_            SSWYTDHQFH.RCELDRVFY..GGWQVIGYSDQIKANRDFFTGRL.........GEVEFVV
XP_013738248|Brassica_napus__rape_                    SSWYTDYQFH.RCELDRVFY..GGWQVVGYSDQIKANRDFFTGRL.........GEVEFVV
XP_010541096|Tarenaya_hassleriana                     SSWYTDPDFH.RLELDRVFY..GGWQAVGFSEQIKEPHHFFTGRL.........GDVEYLV
Kaladp0809s0111.1|Kalanchoe_fedtschenkoi              SSWYTNPSFH.PLELSRVFY..TGWQAVGYSQQLENPGDFFTGSL.........GNVEYVV
Kalax.0333s0026.1|Kalanchoe_laxiflora_corr_462aa      SSWYTNPSFH.PLELSRVFY..TGWQAVGYSQQLENPGDFFTGSL.........GNVEYVV
Kalax.0012s0114.1|Kalanchoe_laxiflora_corr_462aa      SSWYTNPSFH.PLELSRVFY..TGWQAVGYSQQLENPGDFFTGSL.........GNVEYVV
XP_010667950|Beta_vulgaris_subsp._vulgaris__sugar     SSWYFDPSFY.SFEIDHVFY..SGWRVVGCIDQIKNVHDYFTGRL.........GNVEYVI
AH021158-RA|Amaranthus_hypochondriacus_421aa-corr     SSWYFDPSFY.SLELDHVFY..TGWQVVGCIDQIKNAQEYFTGRL.........GDVEYVI
XP_021866412|426|Spinacia_oleracea_(spinach)          CSWYSDPEFY.SHEIDRVFY..SGRVVGCVDQIKNAHDYFTGRL.........GNVEYVI
BAG74777|Hordeum_vulgare_subsp._vulgare__domestic     SGWYTDPGFL.RLELDRVFL..RGWQAVGHIGQVKNPNDFFTGSL.........GNVEFVI
AKZ66518|Triticum_monococcum_subsp._aegilopoides      ...........................................................
FAOM01578495|Triticum_aestivum_UpdateAMA02065         SGWYIDPGFL.RLELDRVFL..RGWQAVGHIGQVKNPNDFFTGSL.........GNVEFVI
EMT07994|Aegilops_tauschii_                           SGWYTDPGFL.RLELDRVFL..RGWQAVGHIGQVKNPNDFFTGSL.........GNVEFVI
ACZ92178|Triticum_turgidum_subsp._durum__durum_wh     ...........................................................
KD266899|Triticum_urartu_UpdateEMS46986               SGWYTDPEFL.RLELDRVFL..RGWQAVGHIGQVKNPNDFFTGSL.........GNVQFVI
ABV64740|Leymus_chinensis                             ..WYTDPEFL.RLELDRVFL..CGWQAVGHIGQVKNPNDFFTGSL.........GNVQFVI
Brast07G193900.1|Brachypodium_stacei                  SEWYTDPEFL.RLELDRVFL..RGWQAVGHIGQVKNPNDFFTGRL.........GNVEFVI
XP_003563491|Brachypodium_distachyon__stiff_brome     SEWYTDPEFL.RLELDRVFL..RGWQAVGHIGQVKNPNDFFSGRL.........GNVEFVI
XP_006657294|Oryza_brachyantha__malo_sina_            SGWYTDPDLL.WLELDRVFL..RGWQAVGHIWQVKNPGDYFTGRL.........GNVEFVV
XP_015643170|Oryza_sativa_Japonica_Group__Japanes     SGWYTDPDFL.RLELDRVFL..RGWQAVGHIWQVKNPNDYFTGRL.........GNVEFVI
EEC81252|Oryza_sativa_Indica_Group__long-grained_     SGWYTDPDFL.RLELDRVFL..RGWQAVGHIWQVKNPNDYFTGRL.........GNVEFVI
XP_002437525|Sorghum_bicolor__sorghum_                SGWYTDPGFL.QHELDRVFH..RGWQAVGHIWQVKNPNDFFTGRL.........GNVEFVI
NP_001105926|Zea_mays                                 SGWYTDPDFL.QLEIDSVFF..RGWQAVGHIWQVKNPNDFFTGRL.........GNVEFVV
Pavir.Db00260.1|Panicum_virgatum_CORR_316f            ..............IDRVFL..RGWQAVGHIWQVENPHDFFTGRL.........GNVEFVI
LWDX02005496.1|Dichanthelium_oligosanthes_update_     SGWYTDPDFL.RLELDRVFL..RGWQAVGHIWQVKNPNDFFTGRL.........GNVEFVI
Sevir.4G257300.1|Setaria_viridis                      SGWYADQDFL.RLELDRVFL..RGWQAVGHIWQVKNPNDFFTGRL.........GNVEFVV
XP_004966128|Setaria_italica__foxtail_millet_         SGWYADQDFL.RLELDRVFL..RGWQAVGHIWQVKNPNDFFTGRL.........GNVEFVV
Oropetium_20150105_16264A|Oropetium_thomaeum          SGWYTDQDFL.RLELDRVFH..RGWQAV.................................
Pahal.D00004.1|Panicum_hallii                         SGWYTDADFL.RLELDRVFL..RGWQAVGHIWQVKNPNDFFTGRL.........GNVEFVI
Pavir.J39646.1|Panicum_virgatum_Corr_363f             SGWYTDADLL.RLELDRVFL..RGWQAVGHIWQVKNPHDFFTGRL.........GNVEFVI
OAY65202|Ananas_comosus__pineapple_                   SMWYIDSSFL.ALEFDRVFF..RGWRAVGGNNRL...................GNVEFVI
XP_009410214|Musa_acuminata_subsp._malaccensis__w     SSWCTDPSFL.ALEFDRVFF..RGWQAVGYTEQIEQPRDFFTGRL.........GNVEFIV
XP_010927268|Elaeis_guineensis__African_oil_palm_     SSWYTDPSFF.ALEFERVFL..RGWQAVGYTEQIKNPRDFFTGRL.........GNVEFVI
XP_008801514|Phoenix_dactylifera__date_palm_          SSWYTDPSFF.AFEFERVFL..RGWQAVGYTEQIKNPHDFFTGRL.........GNVEFVI
KMZ72255|Zostera_marina                               SSWYTDPSFL.DLEFDRVFF..RGWQTVGFTAQVENPNQFFTGRL.........GALQFVV
JAT51600|Anthurium_amnicola                           SSWYTDPSFL.DLEFDRVFF..CGWQAVGYTEQIQNPHDFFTGRL.........GTVQFVV
Spipo5G0003400|Spirodela_polyrhiza                    ...SSDP......................PQQNLISAFLNIRL.........GGVQFVV
XP_006845775|Amborella_trichopoda                     SSWYTDPSFY.ALELEKVFY..KGWQAVGYVEQVKSSHDFFTGRL.........GSVQFVI
AFP19450|Camellia_sinensis                            SSWYTQPSFL.SLEFDRVFF..RGWQAVGCTEQIKESGNFFTGRL.........GNVEYVV
XP_017254907|Daucus_carota_subsp._sativus             SSWYTHPSFL.SLELDRVFY..KGWQAVGYSEQVKEVGDFFTGRL.........GNIEYVV
ABX57826|Chrysanthemum_lavandulifolium                ...........................................................
LEKV01000036.1|Cynara_cardunculus_update_KVI11819     SSWYTCASFL.SLELNQVFF..KGWQAVGCTHQIQEANSFFTGRL.........GNVEYVV
XP_008443631|Cucumis_melo__muskmelon_                 SSWYIDPSFF.ALELDRVFY..RGWQAVGYVEQLKDAHDFFTGRL.........GNVEYVV
XP_004139149|Cucumis_sativus__cucumber_               SSWYIDPSFF.ALELNHVFY..RGWQAVGYVEQLKDAHDFFTGRL.........GNVEYVV
XP_008351406|Malus_domestica__apple_                  SSWYTNPSFY.SLEINTLFY..RGWQAVGYTEQISNAGEFFTGRL.........GNVEFVV
XP_008391562|Malus_domestica__apple_                  SSWYTNPSFY.SLEIXTLFY..RGWQAVGYTEQISNAGEFFTGRL.........GNVEFVV
XP_009343851|Pyrus_x_bretschneideri__Chinese_whit     SSWYTDPSFY.SLELDTLFY..RGWQAVGYTEQIRNAGEYFTGRL.........GNVEFVV
AER10510|Pyrus_betulifolia_corr_405aa                 SSWYTDPSFY.SLELDTLFY..RGWQAVGYTEQIRNAGEFFTGRL.........GNVEFVV
XP_008223853|Prunus_mume__Japanese_apricot_           CSWYTDPSFY.SLELDTLFY..RGWQAAGYTEQIKNPGEFFTCRL.........GNAEFVV
ONI27175|Prunus_persica__peach_                       SSWYTDPSFY.SLELDTLFY..RGWQAAGYTEQIKNPGEFFTCRL.........GNAEFVV
```

**XP_004301419|Fragaria_vesca_subsp._vesca**	SSWYTDPSFY.AQELHSVFY..RGWQAVGYTEQIKNAGDFFTGRL.........GNVEFVV
**XP_015889685|Ziziphus_jujuba__common_jujube_**	SSWYADPSFY.SFELDQIFY..RGWQAIGCTEQIKRPRDFFTGRL.........GNVEFVV
**NW_010360102.1|Morus_notabilis_corrected**	FSWYSLPSFH.SLELDRIFY..KTWQVVGCSEEIKDPGDYFTGRV.........GSVEFVV
**Lus10032689|Linum_usitatissimum**	SSWYTDSSLY.DFELSRVFY..RGWQAVGYTEQIKEPRDFFTGRL.........GNVEFVV
**Lus10008571|Linum_usitatissimum_corrected**	SSWYTDSSLY.DFELSRVFY..RGWQAVGYTEQIKEPRDYFTGRL.........GNIEFVV
**XP_011020077|Populus_euphratica__Euphrates_poplar**	SSWYTDPSFF.DFELHHVFY..KGWQAVGYTEQMKNPRDFFTGRL.........GNVEFLV
**XP_002308100|Populus_trichocarpa__Populus_balsami**	SSWYTDPSFF.DFELHRVFY..KGWQAVGYTEQIKNPRDFFTGRL.........GNVEFLV
**SapurV1A.0198s0060.1|Salix_purpurea**	SSWYTDPSFF.DFELHRVFY..RGWQAVGYTEQIKNPRDFFTGRL.........GNVEFLV
**OAY46667|Manihot_esculenta__cassava_**	SSWYTDPSFY.EYELHRVFY..GGWQAVGYTEQVKDPGDFFTGRL.........GNVEFLV
**XP_002518256|Ricinus_communis__castor_bean_**	SSWYTDPSFY.DYELHCVFY..KGWQAVGFTKQIKSPRDFFTGRL.........GNVEFVV
**XP_012074438|Jatropha_curcas**	SSWYTDPSFF.DYELHRVFY..RGWQAVGYTEQIKDPRDFFTGRL.........GNVEFLV
**XP_010046909|Eucalyptus_grandis**	SSWYTDPSFL.DLELDRVFY..RGWQAVGCTDQVKSPQDFFTGRL.........GNVEFVV
**ABS71853|Eucalyptus_camaldulensis_Murray_red_gum_**	SSWYTDPSFL.DLELDRVFY..RGWQAVGCTDQVKSPQDFFTGRL.........GNVEFVV
**XP_006474292|Citrus_sinensis__sweet_orange_**	SSWYTDPSFL.ALELHRVFY..RSWQVVGYTDQLKDPQDFFSGRI.........GEVEFVV
**XP_006453220|Citrus_clementina_reviewed**	SSWYTDPSFL.ALELHRVFY..RSWQVVGYTDQLKDPQDFFSGRI.........GEVEFVV
**XP_017612508|Gossypium_arboreum**	SSWYTDPSFY.AFELDRVFY..RGWQAVGYTEQIQEPRDFFSGRL.........GNVEFVV
**XP_016686114|Gossypium_hirsutum__cotton_**	SSWYTDPSFY.AFELDRVFY..RGWQAVGYTEQIQEPRDFFSGRL.........GNVEFVV
**XP_012459381|Gossypium_raimondii**	SSWYTDPSFY.AFELDRVFY..RGWQAVGYTEQIQEPRDFFSGRL.........GNVEFVV
**XP_016719040|Gossypium_hirsutum__cotton_**	SSWYTDPSFY.AFELDRVFY..RGWQAVGYTEQIQEPRDFFSGRL.........GNVEFVV
**EOY05290|Theobroma_cacao__cacao_**	SSWYSDPSFY.AFELDRVFY..RGWQAVGYSEQIKEAHDYFTGRL.........GNVEFVV
**AWWV01015497-AWWV01004954_Corchorus_capsularis_39**	TSWYTDPSFY.DFELDRVFY..KGWQAVGYMEQIKEPHDFFSGRL.........GSVEFVV
**OMO51681|Corchorus_olitorius**	SSWYTDPSFY.DFELDRVFY..KGWQAVGYTEQIKEPHDFFSGRL.........GSVEFVV
**GAV59711|Cephalotus_follicularis**	SSWYTHPSFL.SLEFHHVFY..RGWQAVGYTQQIKDPHQYFTGRL.........GDVEYVV
**XP_018807260|Juglans_regia__English_walnut_**	SSWYKHPSFL.ELELHRVFY..RGWQAVGHIQQIKDPHDFFTGRL.........GDVEFVV
**XP_019078540|Vitis_vinifera__wine_grape_**	SSWYTDPSFL.ALELDRVFY..RGWQAVGYTEQIKNPRDFFTGRL.........GNVEFVV
**Aqcoe7G261800.1|Aquilegia_coerulea**	SSWYTDPSFY.NLELNRVFY..RGWQAVGFTEQIKNPHDFFTGRL.........GSVQFVI
**XP_010276721|Nelumbo_nucifera__sacred_lotus_**	SSWYTDPSFL.ELELDRVFY..RGWQAVGVTEQIKNPHDFFTGRL.........GSIEFVI
**XP_016201893|Arachis_ipaensis**	TSWYTDPSFF.QLELHRIFY..RGWQAVGSTEQIRDPNSFFTGRL.........GDVEFVV
**XP_015973237|Arachis_duranensis**	TSWYTDPSFF.QLELHRIFY..RGWQAVGSTEQIKDPNSFFTGRL.........GDVEFVV
**Tp57577_TGAC_v2_mRNA5577|Trifolium_pratense**	SSWYTHRSFF.HHELDRVFY..RGWQAVGSTEQIKDPGDFFTGRL.........GDVEFVV
**GAU38011|Trifolium_subterraneum**	SSWYTHPSFF.HLELDRVFY..RGWQAV................................V
**XP_003610028|Medicago_truncatula__barrel_medic_**	SSWYIDPSFF.HLELDRVFY..RGWQAVGSMEQIKNPGEFFTGRL.........GDVEFVV
**XP_004507911|Cicer_arietinum__chickpea_**	SSWYTHPSFF.HLELDRVFY..KGWQAVGSTEQIKNPGDFFTGRL.........GDVEFVV
**XP_019463425|Lupinus_angustifolius__narrow-leaved**	TSWYTDPSFF.HLELDRVFY..TGWQVVGSTEQIKDALDFFTGRL.........GDVEFVV
**KYP52554|Cajanus_cajan__pigeon_pea_**	TSWYTDPSFF.CHELDRVFY..RGWQVVGSTEQIKDSRDFFTGRL.........GDVEYVV
**XP_003549280|Glycine_max__soybean_**	TSWYTHPSFF.HLELDRVFY..RGWQVVGSTEQIKDPRDYFTGRL.........GDVEYVV
**XP_007134857|Phaseolus_vulgaris_CHR1_corr_208aaf**	....
**XP_007154695|Phaseolus_vulgaris_CORR**	TSWYTDPSFF.HLELDRVFY..RGWQVVGSTEQIKDPRDYFTGRL.........GDVEYVV
**XP_017410379|Vigna_angularis__adzuki_bean_**	TSWYTDPSFF.HLELDRVFY..RGWQVVGSTEQIKDPRDYFTGRL.........GDVEYLV
**XP_014508581|Vigna_radiata_var._radiata__mung_bea**	TSWYTDPSFF.HLELDRVFY..RGWQVVGSTEQIKDPRDYFTGRL.........GDVEYLV
**AUSU01000986.1|Genlisea_aurea_corrected-f**	SSWYNDPDFY.SREIDRVFH..RGWHAVGRKDQVEKVHDYFTGRL.........GSMEYLV
**OAE24413|CORR_Marchantia_polymorpha**	SSWYTNLEIF.SLELEKVFE..RRWQAVGFAHQLQKPGDYFTGKV.........GRVTYVV
**Sphfalx0064s0059.1|Sphagnum**	SAWYTSCDFA.QLEMERVFA..RGWQAVGRVNQLEKPGDYFTGRL.........GNVRYLV
**XP_001752587|Physcomitrella_patens**	SAWYTDAEFA.DFEIDRVFG..RCWQAVGHIKQMEEPGSFFTGRV.........GKTRYVV
**XP_002508933|Micromonas_sp._RCC299**	ASWYFDPLVVPTLEREKVFA..KGWTWAGRLDQVATNGSYLVGAT.........GTVKYVV
**XP_003061830|Micromonas_pusilla_CCMP1545**	SSWYTDAAIIPAIEARAVFA..EGWQAVGRVDQVSDVGDYFTGAV.........GN..FLV
**XP_005645368|Coccomyxa**	SSWYTSQAHT.DSEANRVFS..SSWVYVGHLGSLKESGTFTAGTY.........MGMPYVL
**ADIC01002027.1|Chlorella_variabilis_corrected_pre**	SSWYAQPGVL.EREESAVFQ..RSWLAVAHANRVAAPGAYTAGSL.........LSLEWLA
**XP_013903967|Monoraphidium_neglectum_rev**	ASWYTSPLTP.AREQARVFG..RSWQLVGHVGQVAQPGQYFTGTL.........PPWRYVV
**XP_005644270|Coccomyxa_subellipsoidea_C-169**	SSWYLSPQVA.ELEKVAIFH..NNWQMIGHASQAPNAGDYFTGSV.........ADINYVV
**NC_024001.1|Bathycoccus_prasinos_corrected**	PSYYTSEKLA.QRERATVFSPEKDWVCIGHVSDAPSVNDYFTTTVFSDESENAAAAAVEIVC
**EWM23896|Nannochloropsis_gaditana**	ASWFTDPAFH.TLEMASIFS..DNPLCVGSTLQIPLPGDYFCGQV.........GSHPFIV
**XP_002738379|Saccoglossus_kowalevskii**	STWFTRPEFH.QLEVHTVFH..DAWMFAGRVDQITKPGKFFTGTI.........GKEPYIV
**XP_006820984|Saccoglossus_kowalevskii**	ASWYTHAYFH.DMELKTVFS..NNWLAVGRVDQLKEPGCYFTGSV.........GNRNFVV
**XP_002737044|Saccoglossus_kowalevskii**	SSWYTNPSIL.ELEKRSIFA..NNWVAVGRLNQVETPGQYFQGTF.........ADESFIV
**XP_002597329|Branchiostoma_floridae__Florida_lanc**	ASWYTDGRLH.DLEMRTVFR..RNWVAVGVANQVAKPGQFFTGVL.........GTDPFVV
**XP_019618440|Branchiostoma_belcheri__Belcher's_la**	ASWYTDERLH.DLEMRTVFR..RNWVAVGVTNQVARPGQFFTGLL.........GADPFVV
**XP_019644608|Branchiostoma_belcheri__Belcher's_la**	SSWYRNPGIH.ELEADTVWR..NNWVAVGRTDQVSTPGAFFTGTI.........GREPFIV
**XP_019644788|Branchiostoma_belcheri__Belcher's_la**	SSWYRNPGIH.ELEADTVWR..NNWVAVGRTDQVSTPGAFFTGTI.........GREPFIV
**XP_002610312|Branchiostoma_floridae__Florida_lanc**	.MGYKDSRIH.DLEARTVWR..NNWVAVGRTDQVSTPGAFFTGTI.........GREPFIV
**XP_002599795|Branchiostoma_floridae__Florida_lanc**	SSWFTDPAIY.GLEKRTVFK..WNWVAVGRTDQVASPGQYFTGVV.........GDEPFLV
**XP_019633636|Branchiostoma_belcheri__Belcher's_la**	SSWFTDPAIY.GLEKRTVFK..WNWVAVGRTDQVASPGQYFTGVV.........GDEPFLV
**XP_004333794|Acanthamoeba_castellanii_str._Neff**	ASWYTSPEVF.RREAPLVFH..RGWQQVGRTEQVKDAGQYFTGEL.........LGEPYVV
**GAQ84770|Klebsormidium_flaccidum**	RAWYTERDVF.ELERNSVFA..NRWQAVGPSEKVKEPGAFFSGSF.........MGLQFLV

```
                                                     150       160       170                 180       190
BAC77698|Atriplex_nummularia                         CRDGEGKVHAFHNVCTHRASILA......CGSGK..K..SC...FVCPYHGWVYGM....NG
AGC13074|Atriplex_semibaccata                        CRDGEGNVHAFHNVCTHRASILA......CGSGK..K..SC...FVCPYHGWVYGM....NG
AHH24260|Atriplex_amnicola                           CRDGEGKVHAFHNVCTHRASILA......CGSGK..K..SC...FVCPYHGWVYGM....NG
AFG28558|Atriplex_canescens__four-winged_saltbush    CRDGEGKVHAFHNVCTHRASILA......CGSGK..K..SC...FVCPYHGWVYGM....NG
Q9LKN0|Atriplex_hortensis                            CRDGEGKVHAFHNVCTHRASILA......CGSGK..K..SC...FVCPYHGWVYGM....NG
AAL92561|Atriplex_prostrata                          SRDGEGKVHAFHNVCTHRASILA......CGSGK..K..SC...FVCPYHGWVYGM....NG
O04121|Spinacia_oleracea__spinach_                   SRDGEGKVHAFHNVCTHRASILA......CGSGK..K..SC...FVCPYHGWVYGM....DG
ABG34274|Ophiopogon_japonicus                        SRDGEGKVHAFHNVCTHRASILA......CGSGK..K..SC...FVCPYHGWVYGM....DG
AEW31326|Haloxylon_persicum                          CRDGEGQVHAFHNVCTHRASILA......CGSGK..K..SC...FVCPYHGWVFGM....NG
ACX47904|Haloxylon_ammodendron                       CRDGEGQVHAFHNVCTHRASILA......CGSGK..K..SC...FVCPYHGWVFGM....NG
AJF98574|Salicornia_bigelovii                        CRDGEGKVHAFHNVCTHRASILA......CGSGK..K..SC...FVCPYHGWVFGL....DG
AAV91779|Salicornia_europaea                         CRDGEGKVHAFHNVCTHRASILA......CGSGK..K..SC...FVCPYHGWVFGL....DG
AFW04225|Suaeda_maritima                             SRDGEGKVHAFHNVCTHRASILA......CGSGK..K..SC...FVCPYHGWVFGM....DG
AAM43920|Suaeda_liaotungensis                        SRDGEGKVHAFHNVCTHRASILA......CGSGK..K..SC...FVCPYHGWVFGM....DG
XP_010682183|Beta_vulgaris_subsp._vulgaris__sugar    SRDGQGELHAFHNVCTHRASILA......CGSGK..K..SC...FVCPYHGWVYGL....DG
AHYPO_002206-RA|Amaranthus_hypochondriacus_439       CRDGQGKVHAFHDVCTHRASILA......CGTGK..K..SC...FVCPYHGWVFGL....DG
BAF93187|Amaranthus_tricolor                         CRDGQGKVHAFHNVCTHRASILA......CGTGK..K..SC...FVCPYHGWVFGL....DG
XP_012828802|Erythranthe_guttata__spotted_monkey_    CRDEEGELHAFHNVCRHHASLVA......SGTGK..S..SC...FVCPYHGWTYGL....DG
Migut.J00779.1|Mimulus_guttatus                      CRDEEGELHAFHNVCRHHASLVA......SGTGK..S..SC...FVCPYHGWTYGL....DG
EYU18199|Erythranthe_guttata__spotted_monkey_flow    CRDEEGELYAFHNVCRHRASPVA......SGTGK..S..SC...FVCPYHGWTYGL....DG
KQ992415.1|Dorcoceras_hygrometricum_corrected        CRDEKGMLNAFHNVCRHHASLLV......SGTGK..K..PC...FVCPYHGWTYGL....NG
XP_011094129|Sesamum_indicum__sesame_                CRDENGTLFAFHNVCRHHASLIA......SGTGK..T..SC...FVCPYHGWTYGL....DG
XP_015080536|Solanum_pennellii__Lycopersicon_penn    CRDDGGKVHAFHNVCRHHASLLA......SGSGK..S..SC...FVCPYHGWTYGL....DG
XP_004242785|Solanum_lycopersicum__Lycopersicon_e    CRDDGGKIHAFHNVCRHHASLLA......SGSGK..S..SC...FVCPYHGWTYGL....DG
NP_001275051|Solanum_tuberosum__potato_              CRDDAGKIHAFHNVCRHHASLLA......SGSGK..S..SC...FVCPYHGWTYGL....DG
XP_016556147|Capsicum_annuum                         CRDDGGKVHAFHNVCRHHASLLA......SGSGK..S..SC...FVCPYHGWTYGL....DG
ACR15118|Lycium_barbarum                             CRDDGGKIYAFHNVCRHHATLLA......SGSGK..S..SC...FVCPYHGWTYGL....DG
XP_009757252|Nicotiana_sylvestris__wood_tobacco_     CRDDGGKIHAFHNVCRHHASLLA......SGSGK..R..SC...FVCPYHGWTYGL....DG
XP_016460838|Nicotiana_tabacum_(common_tobacco)      CRDDGGKIHAFHNVCRHHASLLA......SGSGK..R..SC...FVCPYHGWTYGL....DG
OIT26339|Nicotiana_attenuata                         CRDDGGKIHAFHNVCRHHASLLA......SGSGK..N..TC...FVCPYHGWTYGL....DG
XP_009601434|Nicotiana_tomentosiformis               CRDDGGKIHAFHNVCRHHASLLA......SGSGK..R..SC...FVCPYHGWTYGL....DG
XP_016508540|Nicotiana_tabacum_(common_tobacco)      CRDDGGKIHAFHNVCRHHASLLA......SGSGK..R..SC...FVCPYHGWTYGL....DG
XP_015081828|Solanum_pennellii__Lycopersicon_penn    CRDDAGNVRAFHNVCRHRASPLA......SASGK..K..SC...FVCPYHGWTYGL....DG
XP_004243034|Solanum_lycopersicum__Lycopersicon_e    CRDDAGNIRAFHNVCRHRASPLA......SASGK..K..SC...FVCPYHGWTYGL....DG
CDP08949|Coffea_canephora                            CRDGNGNLHAFHNVCRHHASLLA......SGSGQ..K..SC...FVCPYHGWTYGL....DG
XP_019182381|Ipomoea_nil__Japanese_morning_glory_    CRDDSGKIQAFHNVCRHHASVLA......SGSGK..R..TC...FVCPYHGWTYGL....DG
XP_010433050|Camelina_sativa__false_flax_            CRDDNGKIHAFHNVCSHHASILA......SGNGK..K..SC...FVCPYHGWTYSL....SG
XP_010436340|Camelina_sativa_false_flax_corr         CRDDNGKIHAFHNVCSHHASILA......SGNGK..K..SC...FVCPYHGWTYSL....SG
XP_010438247|Camelina_sativa__false_flax_            CRDDNGKIHAFHNVCSHHASILA......SGNGK..K..SC...FVCPYHGWTYSL....SG
XP_010447794|Camelina_sativa__false_flax_            CRDDNGKIHAFHNVCSHHASILA......SGNGK..K..SC...FVCPYHGWTYSL....SG
XP_006285614|Capsella_rubella                        CRDDNGKIHAFHNVCSHHASILA......SGNGK..K..SC...FVCPYHGWTYSL....SG
Cagra.0268s0015.1|Capsella_grandiflora               CRDDNGKIHAFHNVCSHHASILA......SGNGK..K..SC...FVCPYHGWTYSL....SG
Bostr.7867s0836.1|Boechera_stricta                   CRDDNGKIHAFHNVCSHHASILA......SGNGK..K..SC...FVCPYHGWTYSL....SG
NP_194718|Arabidopsis_thaliana__thale_cress_         CRDENGKIHAFHNVCSHHASILA......SGNGR..K..SC...FVCLYHGWTYSL....SG
XP_002869406|Arabidopsis_lyrata_subsp._lyrata        CRDDNGKIHAFHNVCSHHASILA......SGNGK..K..SC...FVCPYHGWTYSL....SG
Araha.6052s0002.1|Arabidopsis_halleri                CREDNGKIHAFHNVCSHHASILA......SGNGR..K..SC...FVCPYHGWTYSL....SG
XP_006412796|Eutrema_salsugineum                     CRDDNGKVHAFHNVCSHHASILA......SGNGK..K..SC...FVCPYHGWTYSL....SG
KFK29548|Arabis_alpina__gray_rockcress_              CRDDNGMIHAFHNVCSHHASILA......SGNGK..K..SC...FVCPYHGWTYSL....SG
KFK40841|Arabis_alpina__gray_rockcress_              CRDDNGMIHAFHNVFSQHASILA......SGNGK..K..SC...FVCPYHGWTYSL....SG
XP_018481725|Raphanus_sativus__radish_               CRDDDGKIHAFHNVCSHHASILA......SGCGK..K..SC...FVCPYHGWTYTL....NG
XP_018435688|Raphanus_sativus__radish_               CRDDDGKIHAFHNVCSHHASILA......SGCGK..K..SC...FVCPYHGWTYAL....NG
XP_013705395|Brassica_napus__rape_                   CRDDDGTINAFHNVCSHHASILA......SGSGR..K..SC...FVCPYHGWTYSL....NG
XP_013596413|Brassica_oleracea_var._oleracea         CRDDDGTINAFHNVCSHHASILA......SGSGR..K..SC...FVCPYHGWTYSL....NG
XP_009137872|Brassica_rapa__field_mustard_           CRDDEGKIRAFHNVCSHHASILA......SGSGR..K..SC...FVCPYHGWTYSL....NG
XP_013738248|Brassica_napus__rape_                   CRDDEGKIRAFHNVCSHHASILA......SGSGR..K..SC...FFCPYHGWTYSL....NG
XP_010541096|Tarenaya_hassleriana                    CRDDNGKVHAFHNVCSHHASILA......SGSGK..K..SC...FVCPYHGWTYGL....SG
Kaladp0809s0111.1|Kalanchoe_fedtschenkoi             CRDLDGKINAFHNVCRHHASLLM......SQSGQ..T..SC...FVCPYHGWTYGL....DG
Kalax.0333s0026.1|Kalanchoe_laxiflora_corr_462aa     CRDLDGKINAFHNVCRHHASLLM......SQSGQ..T..SS...FVCPYHGWTYGL....DG
Kalax.0012s0114.1|Kalanchoe_laxiflora_corr_462aa     CRDLDGKINAFHNVCRHHASLLM......SQSGQ..T..SC...FVCPYHGWTYGL....DG
XP_010667950|Beta_vulgaris_subsp._vulgaris__sugar    CRDAHGEIHAFHNVCRHHASVLA......YGSGR..K..TC...LVCPYHGWTYSL....DG
AH021158-RA|Amaranthus_hypochondriacus_421aa-corr    CRDDDGEIHGFHNVCRHHASVLA......YGSGR..K..TC...FVCPYHGWTYGL....KG
XP_021866412|426|Spinacia_oleracea_(spinach)         CRDGVGKIHAFHNVCRHHASILA......YGSGR..K..TC...FVCPYHGWTYGL....EG
BAG74777|Hordeum_vulgare_subsp._vulgare__domestic    CRDANGKLQAFHNVCRHHASLLA......CGSGQ..K..TC...FQCPYHGWTYGL....DG
AKZ66518|Triticum_monococcum_subsp._aegilopoides     .............................................CPYHGWTYGL....DG
FAOM01578495|Triticum_aestivum_UpdateAMA02065        CRDANGKLQAFHNVCRHHASLLA......CGSGQ..K..TC...FQCPYHGWTYGL....DG
EMT07994|Aegilops_tauschii_                          CRDANGKLQAFHNVCRHHASLLA......CGSGQ..K..TC...FQCPYH.WTYGL....DG
ACZ92178|Triticum_turgidum_subsp._durum__durum_wh    ..............................................................
KD266899|Triticum_urartu_UpdateEMS46986              CRDANGKLQAFHNVCRHHASLLA......CGSGQ..K..TC...FQCPYHGWTYGL....DG
ABV64740|Leymus_chinensis                            CRDGNGKLQALHNVCRHHASLLA......CGSGQ..K..TC...FQCPYHGWTYGL....DG
Brast07G193900.1|Brachypodium_stacei                 CRDANGKLHAFHNVCRHHASLLA......CGSGQ..K..TC...FQCPYHGWTYGL....DG
XP_003563491|Brachypodium_distachyon__stiff_brome    CRDAHGKLHAFHNVCRHHASLLA......CGSGQ..K..TC...FQCPYHGWTYGL....DG
XP_006657294|Oryza_brachyantha__malo_sina_           CRDAIGELHAFHNVCRHHASLLA......CGSGQ..K..TC...FQCPYHGWTYGL....DG
XP_015643170|Oryza_sativa_Japonica_Group__Japanes    CRDANGELHAFHNVCRHHASLLA......CGSGQ..K..TC...FQCPYHGWTYGL....DG
EEC81252|Oryza_sativa_Indica_Group__long-grained_    CRDANGELHAFHNVCRHHASLLA......CGSGQ..K..TC...FQCPYHGWTYGL....DG
XP_002437525|Sorghum_bicolor__sorghum_               CRDADGKLHAFHNVCRHHASLLA......CGSGQ..K..TC...FQCPYHGWTYGL....DG
NP_001105926|Zea_mays                                CRDANGKLHAFHNVCRHHASLLA......CGSGH..K..TC...FQCPYHGWTYGL....DG
Pavir.Db00260.1|Panicum_virgatum_CORR_316f           CRDANGNLNAFHNVCRHHASLLA......CGSGQ..K..TC...FQCPYHGWTYGL....DG
LWDX02005496.1|Dichanthelium_oligosanthes_update_    CRDANGNLHAFHNVCRHHASLLA......CGSGQ..K..TC...FQCPYHGWTYGL....DG
Sevir.4G257300.1|Setaria_viridis                     CRDANGNLHAFHNVCRHHASLLA......CGSGQ..K..TC...FQCPYHGWTYGL....DG
XP_004966128|Setaria_italica__foxtail_millet_        CRDANGNLHAFHNVCRHHASLLA......CGSGQ..K..TC...FQCPYHGWTYGL....DG
Oropetium_20150105_16264A|Oropetium_thomaeum         ....EGKLHAFHNVCRHHASLLA......CGSGQ..K..TC...FQCPYHGWSYGL....DG
Pahal.D00004.1|Panicum_hallii                        CRDANGNLHAFHNVCRHHASILA......CGSGQ..K..TC...FQCPYHGWTYGL....DG
Pavir.J39646.1|Panicum_virgatum_Corr_363f            CRDANGNLNAFHNVCRHHASILA......CGSGQ..K..TC...FQCPYHGWTYGL....DG
OAY65202|Ananas_comosus__pineapple_                  CRDANGNLRAFHNVCRHHASLLT......FGSGQ..K..SC...FECPYHGWTYGL....DG
XP_009410214|Musa_acuminata_subsp._malaccensis__w    CRDTNGTLRAFHNVCRHHASLVA......SGSGQ..K..SC...FVCPYHGWTYGL....DG
XP_010927268|Elaeis_guineensis__African_oil_palm_    CRDANGGLRAFHNVCRHHASLLA......SGSGQ..K..SC...FVCPYHGWTYGL....DG
XP_008801514|Phoenix_dactylifera__date_palm_         CRDANGDLRAFHNVCRHHASLLA......SGSGQ..K..SC...FVCPYHGWTYGL....DG
KMZ72255|Zostera_marina                              CRDADGILHAFHNVCRHHGSVLA......SDCGL..K..TC...FVCPYHGWTYGL....NG
JAT51600|Anthurium_amnicola                          CRDDDGNLHAFHNLCRHHASLLA......YGSGQ..K..SC...FVCPYHGWTYAL....DG
Spipo5G0003400|Spirodela_polyrhiza                   CRDTEGRLRAFHNVCRHHASLLA......RDSGR..K..LC...FVCPYHGWTYGL....DG
XP_006845775|Amborella_trichopoda                    CRDANGDLRAFHNVCRHHASLLA......SGCGQ..K..SC...FVCPYHAWTYGL....DG
AFP19450|Camellia_sinensis                           CRDGNGNVHAFHNVCRHHASLLA......SGSGL..L..SC...FVCPYHGWTYXL....DG
XP_017254907|Daucus_carota_subsp._sativus            CRDDNGELHAFHNVCRHHASLIA......FESGK..K..AC...FTCPYHGWTYGL....DG
ABX57826|Chrysanthemum_lavandulifolium               .........AFHNVCRHHASLLA......FGSGK..G..SC...FVCPYHGWTYGF....DG
LEKV01000036.1|Cynara_cardunculus_update_KVI11819    CRDENGELRAFHNVCRHHASLLA......YGSGK..G..SC...FVCPYHGWTYGL....DG
XP_008443631|Cucumis_melo__muskmelon_                CKDNNRKVRAFHNVCRHHASLLA......TGCGK..K..SC...FVCPYHGWTYGL....DG
XP_004139149|Cucumis_sativus__cucumber_              CKDNNRKVRAFHNVCRHHASLLA......SGCGK..K..SC...FVCPYHGWTYGL....DG
XP_008351406|Malus_domestica__apple_                 CQDGDGKLQAFHNVCRHHAMLLA......YGSGR..K..SC...FVCPYHGWTYGL....DG
XP_008391562|Malus_domestica__apple_                 CQDGDGKLQAFHNVCRHHAMLLA......YGSGR..K..SC...FVCPYHGWTYGL....DG
XP_009343851|Pyrus_x_bretschneideri__Chinese_whit    CQDGDGKLQAFHNVCRHHAMLLA......YGSGR..K..SC...FVCPYHAWTYGL....DG
AER10510|Pyrus_betulifolia_corr_405aa                CQDGDGKLQAFHNVCRHHAMLLA......YGSGR..K..SC...FVCPYHGWTYGF....DG
XP_008223853|Prunus_mume__Japanese_apricot_          CRDGDGKLQAFHNVCRHHASLLA......YGSGR..K..SC...FVCPYHGWTYGL....DG
ONI27175|Prunus_persica__peach_                      CRDGDGKLQAFHNVCRHHASLLA......YGSGR..K..SC...FVCPYHGWTYGL....DG
```

**XP_004301419|Fragaria_vesca_subsp._vesca**	CRDDNGKLQAFHNVCRHHASILA......YGSGR..K..SC...FVCPYHGWTYGL....DG
**XP_015889685|Ziziphus_jujuba__common_jujube_**	CRDDNEKLHAFHNVCRHHASILA......HGSGK..K..SC...FVCPYHGWTYGL....DG
**NW_010360102.1|Morus_notabilis_corrected**	CRDDNGKVRAFHNVCRHHASLLA......YGSGK..K..SC...FVCPYHGWTYGL....DG
**Lus10032689|Linum_usitatissimum**	CRNDGGKIEAFHNVCRHHASLVA......SGSGQ..K..SC...FVCPYHGWTYGL....DG
**Lus10008571|Linum_usitatissimum_corrected**	CRDDDGKVEAFHNVCRHHASLLA......SGSGQ..K..SC...FVCPYHGWTYGL....DG
**XP_011020077|Populus_euphratica__Euphrates_poplar**	CRDDDGKIHAFHNVCRHHASLVA......SGNGQ..K..PC...FVCPYHGWTYGL....DG
**XP_002308100|Populus_trichocarpa__Populus_balsami**	CRDDDGKIHAFHNVCRHHASLVA......SGNGQ..K..SC...FVCPYHGWTYGL....DG
**SapurV1A.0198s0060.1|Salix_purpurea**	CRDDDGKIHAFHNVCRHHASLVA......SGNGQ..K..SC...FVCPYHGWTYGL....DG
**OAY46667|Manihot_esculenta__cassava_**	CRDNDGKLHAFHNVCRHHGSLLA......SGSGK..K..SC...FVCPYHAWTYGL....DG
**XP_002518256|Ricinus_communis__castor_bean_**	CRDDNGKVHAFHNVCRHHGSVLA......SGCGQ..K..SC...FVCPYHAWTYGL....DG
**XP_012074438|Jatropha_curcas**	CRNDDGKVHAFHNVCRHHGSLLA......SGSGQ..K..SC...FVCPYHAWTYGL....DG
**XP_010046909|Eucalyptus_grandis**	CRDDNGKIRAFHNVCRHHASLLA......SGSGK..K..SC...FVCPYHGWTYAL....NG
**ABS71853|Eucalyptus_camaldulensis_Murray_red_gum_**	CRDDNGKIHAFHNVCRHHASLLA......SGSGK..K..SC...FVCPYHGWTYAL....NG
**XP_006474292|Citrus_sinensis__sweet_orange_**	CRDDNGKIHAFHNVCRHHASILA......TGSGK..K..SW...FVCPYHGWTYGL....DG
**XP_006453220|Citrus_clementina_reviewed**	CRDDNGKIHAFHNVCRHHASILA......TGSGK..K..SW...FVCPYHGWTYGL....DG
**XP_017612508|Gossypium_arboreum**	CRDDNGKIAAFHNVCRHHASLLV......SGSGK..T..SC...FTCPYHGWTYGL....NG
**XP_016686114|Gossypium_hirsutum__cotton_**	CRDDNGKIAAFHNVCRHHASLLV......SGSGK..T..SC...FTCPYHGWTYGL....NG
**XP_012459381|Gossypium_raimondii**	CRDDNGKIAAFHNVCRHHASLLV......SGSGK..T..SC...FTCPYHGWTYGL....NG
**XP_016719040|Gossypium_hirsutum__cotton_**	CREDNGKIAAFHNVCRHHASLLV......SGSGK..T..SC...FTCPYHGWTYGL....NG
**EOY05290|Theobroma_cacao__cacao_**	CRDDNGNIAAFHNVCRHHASLLV......SGSGK..K..SC...FTCPYHGWTYEL....NG
**AWWV01015497-AWWV01004954_Corchorus_capsularis_39**	CRDEDGKLQAFHNVCRHHANLLV......SGSGK..T..SC...FTCPYHGWTYGL....NG
**OMO51681|Corchorus_olitorius**	CRDEDGKLQAFHNVCRHHANLLV......SGSGK..T..SC...FTCPYHGWTYGL....NG
**GAV59711|Cephalotus_follicularis**	CRDDSGKLHAFHNVCRHHASLLV......SGTGK..N..LC...FVCPYHAWTYGL....DG
**XP_018807260|Juglans_regia__English_walnut_**	CRDDSGKIHAFHNVCRHHASLLA......SGSGQ..K..SC...FVCPYHAWTYGL....DG
**XP_019078540|Vitis_vinifera__wine_grape_**	CRDNNGKLHAFHNVCRHHASLLA......YGSGQ..K..SC...FVCPYHAWTYGL....DG
**Aqcoe7G261800.1|Aquilegia_coerulea**	CRDANGKVHAFHNVCRHHASLLA......SGSGQ..K..SC...FVCPYHGWTYGL....DG
**XP_010276721|Nelumbo_nucifera__sacred_lotus_**	CRDGNGKLHAFHNVCRHHASLLV......SGSGQ..R..SC...FVCPYHGWTYAL....NG
**XP_016201893|Arachis_ipaensis**	CRDESGKLQAFHNVCRHHASLLA......SGSGH..K..SC...FVCPYHGWTYGL....SG
**XP_015973237|Arachis_duranensis**	CRDESGKLQAFHNVCRHHASLLA......SGSGH..K..SC...FVCPYHGWTYGL....SG
**Tp57577_TGAC_v2_mRNA5577|Trifolium_pratense**	CRDDSGNVRAFYNVCRHHASILA......SGSAK..K..SC...FVCPYHGWTYGL....NG
**GAU38011|Trifolium_subterraneum**	CRDDSGNVRAFHNVCRHHASIIA......SGSGQ..K..SC...FVCPYHGWTYGL....NG
**XP_003610028|Medicago_truncatula__barrel_medic_**	CRDDSGNVRAFYNVCRHHASILA......SGSGQ..K..SC...FVCPYHGWTYGL....NG
**XP_004507911|Cicer_arietinum__chickpea_**	CRDDSGNVHAFYNVCRHHASILA......SGSGQ..K..SC...FVCPYHGWTYGL....NG
**XP_019463425|Lupinus_angustifolius__narrow-leaved**	CRDESGKVRAFHNVCRHHASLLA......SGSGQ..K..SC...FVCPYHGWTYGL....NG
**KYP52554|Cajanus_cajan__pigeon_pea_**	CRDDSGMVRAFHNVCRHHASILA......SGSGN..K..SC...FVCPYHGWTYGF....NG
**XP_003549280|Glycine_max__soybean_**	CRDDSGIVRAFHNVCRHHASLLA......YGSGK..K..SC...FVCPYHGWTYGF....NG
**XP_007134857|Phaseolus_vulgaris_CHR1_corr_208aaf**	....GRTYGF....NG
**XP_007154695|Phaseolus_vulgaris_CORR**	CHDDSGMVRAFHNVCRHHASLLA......YGSGK..K..SC...FVCPYHGWTYGF....NG
**XP_017410379|Vigna_angularis__adzuki_bean_**	CRDDSGMLRAFHNVCRHHASLLA......YGSGK..K..SC...FVCPYHGWTYGF....NG
**XP_014508581|Vigna_radiata_var._radiata__mung_bea**	CRDDSGMVRAFHNVCRHHASLLA......YGSGK..K..SC...FVCPYHGWTYGF....NG
**AUSU01000986.1|Genlisea_aurea_corrected-f**	CQDEALSLRAFHNICRHRATLLT......SGAGN..NPANC...FVCPYHGWTYGL....DG
**OAE24413|CORR_Marchantia_polymorpha**	CKDEQGTLRAYHNVCRHHAAAVA......SGQGC..T..SS...FVCPYHGWIYGL....DG
**Sphfalx0064s0059.1|Sphagnum**	CKDEQEQIHAFHNVCRHHAAAVA......SGSGC..A..HS...FVCPYHGWTYGM....DG
**XP_001752587|Physcomitrella_patens**	CKDGEGQIRAFHNVCRHHAAAVA......SGSGC..T..HS...FVCPYHGWTYGL....DG
**XP_002508933|Micromonas_sp._RCC299**	VRGEDGVLRAFHNACRHHGMEIA....NCPKSTA.....KC...FACPYHGWTYDL....DG
**XP_003061830|Micromonas_pusilla_CCMP1545**	VRGDDGDVRAFHNVCRHHAMEAT.................C...FRCPYHGWTYDD....KG
**XP_005645368|Coccomyxa**	ARSADGQLRAFHNVCRHHAAAVA......LGSGR..Q..DC...FTCPYHGWTYGL....DG
**ADIC01002027.1|Chlorella_variabilis_corrected_pre**	CRDEQGGLHAFHNVCRHHAAILA......QGAGT..A..SC...FQCPYHGWTYGL....DG
**XP_013903967|Monoraphidium_neglectum_rev**	ARGDDGKLRAFHNVCRHHAAAVA......GGCGQ..G..DE...WVCPYHGWTYDT....RG
**XP_005644270|Coccomyxa_subellipsoidea_C-169**	VRGEDGQLRAFHNICRHKAAPVA......EGSGC..V..QM...FECMYHGWQYDH....EG
**NC_024001.1|Bathycoccus_prasinos_corrected**	VRTEENTFKAYENVCQHHFAKVADKKRGTCTAKA..N..SCAAALQCPYHGFTYDVSNEGNG
**EWM23896|Nannochloropsis_gaditana**	LREASGNVKAFYNVCRHKGMRLV.....AEDAGRVGQ..TV...LTCPYHGWEYRL....DG
**XP_002738379|Saccoglossus_kowalevskii**	LRDENNELKAFYNVCRHHAMQIV.....NDTEGT..V..KD...LQCPYHGWTYAL....SG
**XP_006820984|Saccoglossus_kowalevskii**	TRDNRDFLHAFHNVCRHRGMTLC.....SEDSGQ.....TCE..FKCPYHGWTYDL....TG
**XP_002737044|Saccoglossus_kowalevskii**	CRDEKNQLQAFFNVCRHHAMPVV......KGEGS..I..TH...FECPYHGWKYAL....DG
**XP_002597329|Branchiostoma_floridae__Florida_lanc**	VRDQSDQLRAFHNVCRHRANIVV.....RQGEGQ..A..QH...FTCCYHGWTYRL....DG
**XP_019618440|Branchiostoma_belcheri__Belcher’s_la**	VRDQSDQLRAFHNVCRHRANIVV....RQEEGQT.....DQ...FTCCYHGWTYRL....DG
**XP_019644608|Branchiostoma_belcheri__Belcher’s_la**	SSDRSNTLRAFYNVCCHHGNVVE.....WREAGE..A..SE...FVCCYHGWTYAL....DG
**XP_019644788|Branchiostoma_belcheri__Belcher’s_la**	SSDRSNTLRAFYNVCCHHGNVVE.....WREAGE..A..SE...FVCCYHGWTYAL....DG
**XP_002610312|Branchiostoma_floridae__Florida_lanc**	SSDKSNTLRAFYNVCCHHGNIVE.....FQDAGE..A..NQ...FVCCYHGWTYGL....DG
**XP_002599795|Branchiostoma_floridae__Florida_lanc**	VRDKSNILRGFFNVCRHHAAILA...GEDEGTAD..A.......FTCCYHGWTYGL....DG
**XP_019633636|Branchiostoma_belcheri__Belcher’s_la**	VRDKSNTLRGFFNVCRHHAAILA.....GEDEGT..A..DA...FTCCYHGWTYGL....DG
**XP_004333794|Acanthamoeba_castellanii_str._Neff**	VRDQEGQLRAFFNVCRHHASKVA......TGEGC..K..SE...LVCPYHGWTYQL....DG
**GAQ84770|Klebsormidium_flaccidum**	CRTLQGELKAFSNTCRHKGTVIA......QGEGT..C..RN...FVCPFHAWTYNL....DG

	200 210 220 230
BAC77698|Atriplex_nummularia	SLTKASKATAEQSLNPDELGLVPLKVAEWGPFILISLDRS......SRE.............
AGC13074|Atriplex_semibaccata	SLTKASKATAEQSLNPDELGLVPLKVAVWGPFILISLDRS......SRE.............
AHH24260|Atriplex_amnicola	SLTKASKATAEQSLNPDELGLVPLKVAVWGPFILISLDRS......SRE.............
AFG28558|Atriplex_canescens__four-winged_saltbush	SLTKASKATAEQSLNPDELGLVPLKVAVWGPFILISLDRS......SRE.............
Q9LKN0|Atriplex_hortensis	SLTKASKATPEQSLNPDELGLVPLKVAVWGPFILISLDRS......SRE.............
AAL92561|Atriplex_prostrata	TLTKASKATAEQSLNPDELGLVPLKVAEWGPFILISLDRS......SRE.............
O04121|Spinacia_oleracea__spinach_	SLAKASKAKPEQNLDPKELGLVPLKVAVWGPFVLISLDRS......LEE.............
ABG34274|Ophiopogon_japonicus	SLAKASKAKPEQNLDPKELGLVPLKVAVWGPFVLISLDRS......LEE.............
AEW31326|Haloxylon_persicum	DLTKATQ.AETQTFDPKELGLVALKVAVWGPFVLISVDKD......LPE.............
ACX47904|Haloxylon_ammodendron	DLTKATQ.AETQTFDPKELGLVALKVAVWGPFVLISVDKD......LPE.............
AJF98574|Salicornia_bigelovii	DLTKATQTTDAQTFDPKEYGLVPLKVAEWGPFVLISVDKN......LPE.............
AAV91779|Salicornia_europaea	DLTKATQTTDAQTFDPKEYGLVPLKVAEWGPFVLISVDKN......LPE.............
AFW04225|Suaeda_maritima	DLTKATQTTDAQTFDPKEYGLVPLKVAVWGPIVLISLDIT......RPE.............
AAM43920|Suaeda_liaotungensis	DLTKATQTTDAQTFDPKEYGLKPLKVAVWGPFVLISLDKT......LPE.............
XP_010682183|Beta_vulgaris_subsp._vulgaris__sugar	SLAKASKATETQNLDPKELGLAPLKVAEWGPFILISLDRS......LDA.............
AHYPO_002206-RA|Amaranthus_hypochondriacus_439	SLMKATK.TENQVFDPQELGLVSLKVAIWGPFVLISLDRS......GSE.............
BAF93187|Amaranthus_tricolor	SLMKATK.TENQVFDPQELGLVTLKVAIWGPFVLISLDRS......GSE.............
XP_012828802|Erythranthe_guttata__spotted_monkey_	KLVKATRITGIKNFKANEMGLVPIRVATWGPFVLVNFEDG..AT.PNEI.............
Migut.J00779.1|Mimulus_guttatus	KLVKATRITGIKNFKANEMGLVPIRVATWGPFVLVNFEDG..AT.PNEI.............
EYU18199|Erythranthe_guttata__spotted_monkey_flow	KLIKATSITGIKNFEPSETGLVSIRVAVWGPFVLVNFEDG..AN.PNEI.............
KQ992415.1|Dorcoceras_hygrometricum_corrected	GLMKATRITGIKNFKVNEMGLVPLEVAIWGPFVLINFSKG..MR.HEED.............
XP_011094129|Sesamum_indicum__sesame_	ALVKATRITGIKNFKVNEMGLVPLKVGIWGPFVLINFDGG..MM.PQKG.............
XP_015080536|Solanum_pennellii__Lycopersicon_penn	ALLKATRITGIKNFKVNEMGLIPMRVAVWGPFILLNFENG..VL.PEQE.............
XP_004242785|Solanum_lycopersicum__Lycopersicon_e	ALLKATRITGIKNFKVNEMGLIPMRVAVWGPFILLNFENG..VL.PEQE.............
NP_001275051|Solanum_tuberosum__potato_	ALLKATRITGIKNFKVNEMGLVPMRVAVWGPFILVNFENG..VL.PEQE.............
XP_016556147|Capsicum_annuum	ALLKATRITGIKNFKVNEMGLVPMRVAIWGPFILLNFDNG..VF.PEQE.............
ACR15118|Lycium_barbarum	ALLKATRITGIKNFKVNEMGLVPMRVAVWGPFILLNFENG..AL.SEQK.............
XP_009757252|Nicotiana_sylvestris__wood_tobacco_	TLLKATRITGIKNFKVNEMGLVPMKVAVWGPFILLNFENG..VL.YEQE.............
XP_016460838|Nicotiana_tabacum_(common_tobacco)	TLLKATRITGIKNFKVNEMGLVPMKVAVWGPFILLNFENG..VL.YEQE.............
OIT26339|Nicotiana_attenuata	ALLKATRITGIKNFKVNEMGLVPMKVAVWGPFILLNFENG..VL.SEQE.............
XP_009601434|Nicotiana_tomentosiformis	ALLKATRITGIKNFKVNEMGLVPMKVAVWGPFILLNFENG..VL.SEQE.............
XP_016508540|Nicotiana_tabacum_(common_tobacco)	ALLKATRITGIKNFKVNEMGLVPMKVAVWGPFILLNFENG..VL.SEQE.............
XP_015081828|Solanum_pennellii__Lycopersicon_penn	ALQKATRITGIKNFRVNEMGLVPVRVAVWGPFILLNFENG..VL.PENE.............
XP_004243034|Solanum_lycopersicum__Lycopersicon_e	ALQKATRITGIKNFRVNEMGLVPVRVAVWGPFILLNFENG..VL.PENE.............
CDP08949|Coffea_canephora	TLLKATRISGIKNFRVHEMGLVPLRVAIWGPFVLLNFETE..NL.LQQE.............
XP_019182381|Ipomoea_nil__Japanese_morning_glory_	ALLKATRLTGIQKFEVNEMGLIPLKVAIWGPFVLLNFDAA..VS.PEHE.............
XP_010433050|Camelina_sativa__false_flax_	SLVKATRMNGIENFALNEMGLKPLRVAVWGPFVLLKVTQA..RSRKGED.............
XP_010436340|Camelina_sativa_false_flax_corr	SLVKATRMNGIENFALNEMGLKPLRVAVWGPFVLLKVTQA..RSRKGED.............
XP_010438247|Camelina_sativa__false_flax_	SLVKATRMTGIENFALNEMGLKPLRVAVWGPFVLLKVTQA..TSRKGED.............
XP_010447794|Camelina_sativa__false_flax_	SLVKATRMNGIENFALNEMGLKPLRVAVWGPFVLLKVTQA..TSRKGED.............
XP_006285614|Capsella_rubella	SLVKATRMTGIENFALNEMGLKPLRVAVWGPFVLLKVTQA..TSRMGED.............
Cagra.0268s0015.1|Capsella_grandiflora	SLVKATRMTGIENFALNEMGLKPLRVAVWGPFVLLKVPQA..TSRIGED.............
Bostr.7867s0836.1|Boechera_stricta	SLVKATRITGIENFALNEMGLKPLRVAVWGPFVLLKVAQA..TSRNEEL.............
NP_194718|Arabidopsis_thaliana__thale_cress_	SLVKATRMSGIQNFSLSEMGLKPLRVAVWGPFVLLKVTAA..TSRKGEV.............
XP_002869406|Arabidopsis_lyrata_subsp._lyrata	SLVKATRMTGIENFALNEMGLKPLKVAVWGHFVLLKVTQA..TSRDGEV.............
Araha.6052s0002.1|Arabidopsis_halleri	SLVKATRMNGIENFALSEMGLKPLKVAVWGPFVLLKVTQA..TSRDGEV.............
XP_006412796|Eutrema_salsugineum	SLVKANRMTGIQSFALHEMGLKPLRVAVWGPFVLMKVAQA..TSRKGEV.............
KFK29548|Arabis_alpina__gray_rockcress_	SLVKATRMTGIENFALNEMGLKPLRVAAWGPFVLLKMTKT..PLSQEEV.............
KFK40841|Arabis_alpina__gray_rockcress_	SLVKASRMNGIENFALNEMGLKPLRVAAWGPFVLLKMTKT..PLSQEEV.............
XP_018481725|Raphanus_sativus__radish_	SLVKATRMTGIENFALNEMGLKPLRVAVWGPFVLLKVVNHATSSKKEED.............
XP_018435688|Raphanus_sativus__radish_	SLVKATRMTGIHNFPLNEMGLKPLTVAIWGPFVLLKVAQD..ESNKEED.............
XP_013705395|Brassica_napus__rape_	SLVKATRMTGIENSALNEMGLKPLRVAVWGPFVLLKVTQA..TWKKEED.............
XP_013596413|Brassica_oleracea_var._oleracea	SLVKATRTTGIENSALNEMGLKPLRVAVWGPFVLLKVTQA..TWKKEED.............
XP_009137872|Brassica_rapa__field_mustard_	SLVKATRMTGIENSALNEMGLKPLRVAVWGPFVLLKVTQD..KSKKKED.............
XP_013738248|Brassica_napus__rape_	SLVKATRMTGIENFALNEMGLKPLRVAVWGPFVLLKVTQD..KSKKEED.............
XP_010541096|Tarenaya_hassleriana	SLVKATRMTGIENFYSQEMGLKPISVAVWGPFVLLNMNQN..TSNWEEF.............
Kaladp0809s0111.1|Kalanchoe_fedtschenkoi	SLAKATRITGIKNFNVKEFGLIPIKVGAWGPFILLNLGAA..TS.TQQN.............
Kalax.0333s0026.1|Kalanchoe_laxiflora_corr_462aa	SLAKATRITGIKNFNVKEFGLIPIKVGAWGPFILLNLGAA..TS.TQQN.............
Kalax.0012s0114.1|Kalanchoe_laxiflora_corr_462aa	SLAKATRITGIKNFNVKEFGLIPIKVGAWGPFILLNLGAA..TS.TQQN.............
XP_010667950|Beta_vulgaris_subsp._vulgaris__sugar	NLLKAPRITGIQNFDPKEFGLIPISVATWGPFVVVNLSAS......REE.............
AH021158-RA|Amaranthus_hypochondriacus_421aa-corr	NLLKAPRITGIQKFDPMEFGLIPICVATWGPFVVINVGSS......EKK.............
XP_021866412|426|Spinacia_oleracea_(spinach)	NLLKAPRITGLRNFNPKEYGLVPINVATWGPFVVVNLSSS......EEE.............
BAG74777|Hordeum_vulgare_subsp._vulgare__domestic	TLLKATRISGIKNFNKNDFGLLPIKVATWGPFVLARFDD......SSQD.............
AKZ66518|Triticum_monococcum_subsp._aegilopoides	ILLKATRISGIKNFNKNDFGLLPIKVATWGPFVLARFDD......SSQD.............
FAOM01578495|Triticum_aestivum_UpdateAMA02065	TLLKATRISGIKNFNKNDFGLLPIKVATWGPFVLARFDN......SSQD.............
EMT07994|Aegilops_tauschii_	TLLKATRISGIKNFNKNDFGLLPIKVATWGPFVLARFDD......SSQD.............
ACZ92178|Triticum_turgidum_subsp._durum__durum_wh	....
KD266899|Triticum_urartu_UpdateEMS46986	TLLKATRISGIKNFNKNDFGLLPIKVATWGPFVLARFDD......SSQD.............
ABV64740|Leymus_chinensis	TLLKATRISGIKNFNKNDFGLLPIKVATWGPFVLARFDD......SSQD.............
Brast07G193900.1|Brachypodium_stacei	VLLKATRISGIKNFNKNDFGLLPIKIATWGPFVLARFDD......FAED.............
XP_003563491|Brachypodium_distachyon__stiff_brome	ALLKATRISGIKNFNKNDFGLLPIKVATWGPFVLARFDN......FAQD.............
XP_006657294|Oryza_brachyantha__malo_sina_	VLLKATRIAGIKNFNKNDFGLIPIKVATWGPFVVARFDNG.....FSKE.............
XP_015643170|Oryza_sativa_Japonica_Group__Japanes	VLLKATQISGIKNFNKNDFGLIPIKVATWGPFVLAKFDSG.....FSQE.............
EEC81252|Oryza_sativa_Indica_Group__long-grained_	VLLKATRISGIKNFNKNDFGLIPIKVATWGPFVLAKFDSG.....FSQE.............
XP_002437525|Sorghum_bicolor__sorghum_	TLLKATRISGIRNFNKNDFGLIPIKVVTWGPFVLVRFDDE.....STED.............
NP_001105926|Zea_mays	TLLKATRISGIKNFNKNDFGLIPIKVATWGPFVLIRFDDE.....STED.............
Pavir.Db00260.1|Panicum_virgatum_CORR_316f	TLLKATRISGIKNFNKNDFGLVPVKVATWGPFVLARFDDE.....STEG.............
LWDX02005496.1|Dichanthelium_oligosanthes_update_	TLLKATRISGIKNFNKNDFGLVPIKVAAWGPFVLARFDDG.....STEG.............
Sevir.4G257300.1|Setaria_viridis	TLLKATRISGIKNFNKNDFGLVPIKVATWGPFVLARFDDE.....STED.............
XP_004966128|Setaria_italica__foxtail_millet_	TLLKATRISGIKNFNKNDFGLVPIKVATWGPFVLARFDDE.....STED.............
Oropetium_20150105_16264A|Oropetium_thomaeum	TLLKATRISGIKNFNKNDFGLIPIKVATWGPFVLARFDGE.....PTED.............
Pahal.D00004.1|Panicum_hallii	TLLKATRISGIKNFNKNDFGLVPVKVATWGPFVLVRFDDE.....STEG.............
Pavir.J39646.1|Panicum_virgatum_Corr_363f	TLLKATRISGIKNFNKNDFGLVPVKVATWGPFVLARFDDE.....STEG.............
OAY65202|Ananas_comosus__pineapple_	ELLKATRISGIKNFNKNNFGLKSINVGTWGPFVLVNLDQK..IL.NQQD.............
XP_009410214|Musa_acuminata_subsp._malaccensis__w	TLLKATRITGIKNLKKDEFGLIPLAVATWGPFVLIRFDRH..PV.PQQN.............
XP_010927268|Elaeis_guineensis__African_oil_palm_	VLLKATRITGIKNFNKDEFGLIPLSVATWGPFVLLNLDTD..IV.PQQN.............
XP_008801514|Phoenix_dactylifera__date_palm_	ALLKATRITGIKNFNKDEFGLMPLSVATWGPFVLINLDKD..TV.PQQN.............
KMZ72255|Zostera_marina	QLLKATHITGIQDFNEKDYGLVPLKVATWGPFILISMDKD..LL.HHQD.............
JAT51600|Anthurium_amnicola	GLLNATRIFGIHNFNVKDFGLIPLNVATWGPFVLINMDKN......KST.............
Spipo5G0003400|Spirodela_polyrhiza	ALLKATRVSGIQNFSVDDLGLIPLNVATWGPFVLVSMDEG..GPARGAV.............
XP_006845775|Amborella_trichopoda	RLLKATRISGIQKFNVHDYGLQPINVATLGPFVLINMDSS..NTNSANE.............
AFP19450|Camellia_sinensis	ALLKATRITGIRNFNVNEFGLIPLKVAIWGPFVLLNLEDI......PPQ.............
XP_017254907|Daucus_carota_subsp._sativus	TLLKATRITGIQNFNVKEFGLVPLGVASWGPFILLNLESE..HF.PHQQ.............
ABX57826|Chrysanthemum_lavandulifolium	ALLKATRITGIRNFNVKEFGLVPLRVAIWGPFILLNIERQ..AF.VQQH.............
LEKV01000036.1|Cynara_cardunculus_update_KVI11819	RLLKATRITGIKNFNVKEFGLVPVRVAIWGPFILLNLERK..GF.DQQN.............
XP_008443631|Cucumis_melo__muskmelon_	ILLKATRINGIQNFNENDFGLVPLPVAMWGPFVLLNLDGK..LS.SKPD.............
XP_004139149|Cucumis_sativus__cucumber_	ILLKATRIDGIQNFDENDFGLVPLPVATWGPFVLLNLDGK..LS.SKPD.............
XP_008351406|Malus_domestica__apple_	ALLKATRITGIQNFNENEFGLVPLKVATWGPFILLNMEQN..V.....D.............
XP_008391562|Malus_domestica__apple_	ALLKATRITGIQNFNENEFGLVPLKVATWGPFILLNMEQN..V.....D.............
XP_009343851|Pyrus_x_bretschneideri__Chinese_whit	ALLKATRITGIQNFNEHEFGLVPLKVATWGPFILLNMEQN..V.....D.............
AER10510|Pyrus_betulifolia_corr_405aa	ALLKATRITGIQNFNEHEFGLVPLKVATWGPFILLNMEQN..V.....D.............
XP_008223853|Prunus_mume__Japanese_apricot_	SLLKATRITGIQNFNEHEFGLISVKVATWGPFVLLNMEQD..V.....D.............
ONI27175|Prunus_persica__peach_	SLLKATRITGIQNFNEHEFGLISVKAATWGPFVLLNMEQD..V.....D.............

```
XP_004301419|Fragaria_vesca_subsp._vesca           ALRKATRITGIQNFNKHEFGLTPVKVATWGPFVLANMEPE..V.....D.............
XP_015889685|Ziziphus_jujuba__common_jujube_       ALLKASRLAGIKNFVEHEFGLIPIKVATWGPFVLLNMEQD..V.....D.............
NW_010360102.1|Morus_notabilis_corrected           PLLKATRITGIQNFNVNEFGLIPIQVAIWGPFVLLNMEQK..V.....T.............
Lus10032689|Linum_usitatissimum                    SLRKATRITEIHNFDVNDFGLIPIDVAVWGPFVLLNLERN..CSRPKYD.............
Lus10008571|Linum_usitatissimum_corrected          SLRKATRITGIHNFDVNDFGLIPIDVAVWGPFVLLNLERN..CSRPKYD.............
XP_011020077|Populus_euphratica__Euphrates_poplar  ALLKATRITGIQNFDVNEFGLKPLNVATWGPFVLLNLDKE..IL.PQKE.............
XP_002308100|Populus_trichocarpa__Populus_balsami  ALLKATRITGIQNFDVNEFGLKPLNVATWGPFVLLNLDKE..IL.PQQE.............
SapurV1A.0198s0060.1|Salix_purpurea                ALLKATRMNRIQNFDVNEFGLKLLNVATWGPFVLLNLEKE..IL.PQQE.............
OAY46667|Manihot_esculenta__cassava_               GLLKATRITGMQNFKVNEFGLIPLNVATWGPFVLLNVGKE..SS.THQE.............
XP_002518256|Ricinus_communis__castor_bean_        ALLKATRITGMQNFSLDEYGLLPINVAAWGPFVLLNVDRE..SS.PQQE.............
XP_012074438|Jatropha_curcas                       ALLKATRITGMQNFNLNEFGLIPINVATWGPFILLNVEKE..SK.LQQG.............
XP_010046909|Eucalyptus_grandis                    ALLRATRVHGIRNFDVKEFGLIPLKVATWGPFVLLNFEKD..VS.PDQE.............
ABS71853|Eucalyptus_camaldulensis_Murray_red_gum_  ALLRATRVHGIRNFDVKEFGLIPLKVATWGPFVLLNFEKD..VS.PDQE.............
XP_006474292|Citrus_sinensis__sweet_orange_        TLLKATRITGIKDFNVKEFGLVPLEVATWGPFVLLNMGKE..AV.HQEE.............
XP_006453220|Citrus_clementina_reviewed            TLLKATRITGIKDFNVKEFGLVPLEVATWGPFVLLNMGKE..AV.HQEE.............
XP_017612508|Gossypium_arboreum                    ELRKATRISGIDDFSINDFGLVPIKVATWGPFVLLNMDNE..IL.QKDN.............
XP_016686114|Gossypium_hirsutum__cotton_           ELRKATRISGIDDFSINDFGLVPIKVATWGPFVLLNMDNE..IL.QKDN.............
XP_012459381|Gossypium_raimondii                   ELRKATRISGIEDFSINDFGLVPIKVATWGPFVILNMDNE..IL.QKDN.............
XP_016719040|Gossypium_hirsutum__cotton_           ELRKATRISGIEDFSINDFGLVPIKVATWGPFVLLNMDNE..IL.QKDN.............
EOY05290|Theobroma_cacao__cacao_                   ALRKATRISGIQDFIVNEFGLIPIKVATWGPFVLLNMDKE..IL.QKDD.............
AWWV01015497-AWWV01004954_Corchorus_capsularis_39  ALRKATRISGIKKFSVNEFGLIPIKVATWGPFILLNMDKD..IL.PKAD.............
OMO51681|Corchorus_olitorius                       ALRKATRISGIKKFSVNEFGLIPITVATWGPFILLNMDKD..IL.PKAD.............
GAV59711|Cephalotus_follicularis                   ALLKATRITGIQNFNVEEFGLIPLNVATWGPFILLNLDQE..VS.PGQE.............
XP_018807260|Juglans_regia__English_walnut_        HLVKATRIMGIQNFNVNEFGLIPIKVATWGPFILLNLDKW..IS.PLRE.............
XP_019078540|Vitis_vinifera__wine_grape_           ALLKATRITGIKDFSINEFGLIPLRIATWGPFVLLNINND..VS.PQHE.............
Aqcoe7G261800.1|Aquilegia_coerulea                 KLLKATRISEIQNFKLDEFGLIPVQVAIWGPFILINIDKG..IE.PQQQ.............
XP_010276721|Nelumbo_nucifera__sacred_lotus_       VLLKATRISGLKNFAINEFGLIPLKVTTWGPFVLINLDKE..IQ.PQQE.............
XP_016201893|Arachis_ipaensis                      ALLKATRISGMRNFNVKDFGLIPIKVSTWGPFILLNLEKE..SL.PLME.............
XP_015973237|Arachis_duranensis                    ALLKATRISGMRNFNVKDFGLIPIKVSTWGPFILLNLEKE..SL.PLME.............
Tp57577_TGAC_v2_mRNA5577|Trifolium_pratense        NLVKATRISGMRDFNGNDFGLIPIKVATWGPFVLLNLEKE..NL.SQKE.............
GAU38011|Trifolium_subterraneum                    NFVKATRISGMRDFNGNDFGLIPIKVATWGPFVLLNLEKE..NL.SQKE.............
XP_003610028|Medicago_truncatula__barrel_medic_    NLLKATRISGMRDFNVNDFGLIPIKVATWGPFVLLNFGKE......KEE.............
XP_004507911|Cicer_arietinum__chickpea_            TLLKATRISGMRDFNVNEFGLIPIKVATWGPFVLLNLKKE..NL.SEKE.............
XP_019463425|Lupinus_angustifolius__narrow-leaved  ALLKATRISGMRDFNLKDFGLIPIKVATWGPFVLINLEKE..SL.SLNE.............
KYP52554|Cajanus_cajan__pigeon_pea_                ALLKATRISGMRNFNVNNFGLLPIKVAIWGPFVLLNLDKE..SL.SQKE.............
XP_003549280|Glycine_max__soybean_                 ALLKATRISGMRNFNVNDFGLLPMKVATWGPFVLLNLEKE..NL.SKKE.............
XP_007134857|Phaseolus_vulgaris_CHR1_corr_208aaf   ALIKATRISGMRNFNINDFGLLPIKAATWGRFVLLNLEKE..NV.SQKK.............
XP_007154695|Phaseolus_vulgaris_CORR               ALRKATRISGMRNFNVNDFGLLPIKAATWGPFVLLNLEKE..NV.SQKE.............
XP_017410379|Vigna_angularis__adzuki_bean_         ALLKATRISGMRNFNVNDFGLLPIKVATWGPFILLNLEKE..NL.SQKE.............
XP_014508581|Vigna_radiata_var._radiata__mung_bea  ALLKATRISGMRNFNVNDFGLLPIKVATWGPFILLNLEKE..NL.SQKE.............
AUSU01000986.1|Genlisea_aurea_corrected-f          SLLKPTRTDGIENFRIHEMGLLPLDVARWGPFILINAAHH......................
OAE24413|CORR_Marchantia_polymorpha                RLQKATRIGGMRNFTARESGLSRLSVGTWGPFVLIKSPEQ......GAV.............
Sphfalx0064s0059.1|Sphagnum                        KLQKATRLAGIRNFSARENGLVPIRTATWGPFVLITLEGN.....KTAH.............
XP_001752587|Physcomitrella_patens                 RLQKASRLAGIRNFSARENGLVPIRTATWGPFVLISLAES.....KASE.............
XP_002508933|Micromonas_sp._RCC299                 SLVKATKIGGMRDFVVAEHGLKPIRVAEWGPFVMLRLGDP...ATEEEE.............
XP_003061830|Micromonas_pusilla_CCMP1545           ALTKATRLTGIEGFDVSDNGLKPLRVDTWGPFVFVNLGGD..DASAPPP.............
XP_005645368|Coccomyxa                             RLRRAPHMRGIENFRAADYGLRQLRMRTWGHFIFLNFDSNDITCCTGVG.............
ADIC01002027.1|Chlorella_variabilis_corrected_pre  RLQRATRLKGIEDFRAADHGLVPLQVEEWGGFVWVRQRHFYGGSSRPAAASASSGGGSKAIR
XP_013903967|Monoraphidium_neglectum_rev           RLRKAPRVKGIKDFRAAEWGLRPIALDTWGPFVFLWMGDG..SGRSSSSSSSSSSGG.....
XP_005644270|Coccomyxa_subellipsoidea_C-169        RFRGATKLKGIKNFKAQDYGLVPIRLDTWGQFVFILADPT..SERGSVA.............
NC_024001.1|Bathycoccus_prasinos_corrected         KLISATRMKGMEERTEKIKLKGGYVCETFGPFVFLKKDES.....SSSSSSSS.........
EWM23896|Nannochloropsis_gaditana                  RLAKATRVAGIRDFAPKDYGLLEIPLWTWGPLVFLRFGQA.....ENEA.............
XP_002738379|Saccoglossus_kowalevskii              RLKKATRLRGIKNFSARNFGLIPMMVKAWGPLIFIRPNKI...SEEKDN.............
XP_006820984|Saccoglossus_kowalevskii              RLTRAKKLRGIKNFSASKYGLKPLQVSTWGPLVFVNANDT....ANESE.............
XP_002737044|Saccoglossus_kowalevskii              RLTLARKMRGIQDFKARDYGLIPMPVTSWGPLVMVKLIPG..RDGGEDD.............
XP_002597329|Branchiostoma_floridae__Florida_lanc  RLSKALRLRGIKNFSARENGLKPVAVRTWGPVVLVQLEGD.....GRED.............
XP_019618440|Branchiostoma_belcheri__Belcher’s_la  RLSKALRLRRIKNFSARENGLKPVAVRTWGPAVLVRLEGD.....GRTD.............
XP_019644608|Branchiostoma_belcheri__Belcher’s_la  RLTKPTKLRGIKNFVAAQNGLKPIGVRTWGPLVFLRLAGY....PDEDD.............
XP_019644788|Branchiostoma_belcheri__Belcher’s_la  RLRKPTKLRGIKNFVAAQNGLKPIGVRTWGPLVFLRLAGY....PDEDD.............
XP_002610312|Branchiostoma_floridae__Florida_lanc  RLTKPTKLRGIKNFTARGNGLKPIAVRTWGPLVFLRLDGY....PDEDE.............
XP_002599795|Branchiostoma_floridae__Florida_lanc  RLTKAMRLRGIQNFSARNFGLKPMAVKAWGPLVLVRPDGQ....ENGTD.............
XP_019633636|Branchiostoma_belcheri__Belcher’s_la  RLTKAMRLRGIQNFSARNFGLKPMAVKAWGPLVLVRPDGQ....EDGTD.............
XP_004333794|Acanthamoeba_castellanii_str._Neff    RLRVAPRVGGIKEFKPKENGLVPIQVIEWGRWIFINIDSS.....SDTD.............
GAQ84770|Klebsormidium_flaccidum                   SLIGSKHHKHMKDFNPEEHSLLPLDVAIAGPWILVRMPNWGNK...APASPVYVNDEMAQNW
```

```
                                                                                240        250        260       270       280
                                                                                 .          .          .         .         .
BAC77698|Atriplex_nummularia                                  ...VGDV....GSE.....WLG.SCAEDVKA.HAF.DP..NLQFIHRSEFPIESNWKIFSDN
AGC13074|Atriplex_semibaccata                                 ...VGDV....GSE.....WLG.SCAEDVKA.HAF.DP..NLQFIHRSEFPIESNWKIFSDN
AHH24260|Atriplex_amnicola                                    ...VGDV....GSE.....WLG.SCAEDVKA.HAF.DP..NLQFIHRSEFPIGSNWKIFSDN
AFG28558|Atriplex_canescens__four-winged_saltbush             ...VGDV....GSE.....WLG.SCAEDVKA.HAF.DP..NLQFIHRSEFPIESNWKIFSDN
Q9LKN0|Atriplex_hortensis                                     ...VGDV....GSE.....WLG.SCAEDVKA.HAF.DP..NLQFINRSEFPIESNWKIFSDN
AAL92561|Atriplex_prostrata                                   ...VGDV....GSE.....WLG.SCAEDVKA.HAF.DP..NLQFINRSEFPIESNWKIFSDN
O04121|Spinacia_oleracea__spinach_                            ...GGDV....GTE.....WLG.TSAEDVKA.HAF.DP..SLQFIHRSEFPMESNWKIFSDN
ABG34274|Ophiopogon_japonicus                                 ...GGDV....GTE.....WLG.TSAEDVKA.HAF.DP..SLQFIHRSEFPMESNWKIFSDN
AEW31326|Haloxylon_persicum                                   ....TDV....GTE.....WLG.KTAEDVKA.HAF.DP..ALQFIHRSEFPMECNWKVFCDN
ACX47904|Haloxylon_ammodendron                                ....TDV....GTE.....WLG.KTAEDVKA.HAF.DP..ALQFIHRSEFPMECNWKVFCDN
AJF98574|Salicornia_bigelovii                                 ....TDP....GTE.....WLG.SSAEDVKA.HAF.DP..NLQFIHRSEFPMECNWKVFCDN
AAV91779|Salicornia_europaea                                  ....TDP....GTE.....WLG.SSAEDVKA.HAF.DP..NLQFIHRSEFPMECNWKVFCDN
AFW04225|Suaeda_maritima                                      ....SDV....GTE.....WLG.SSAEDVKA.HAF.DP..SLKFIHRSEFPMECNWKVFSDN
AAM43920|Suaeda_liaotungensis                                 ....SDV....GTE.....WLG.SSAEDVKA.HAF.DP..SLKFIHRSEFPMECNWKVFCDN
XP_010682183|Beta_vulgaris_subsp._vulgaris__sugar             ...NADV....GTE.....WIG.KSAEDVKA.HAF.DP..NLKFTHRSEFPMECNWKVFCDN
AHYPO_002206-RA|Amaranthus_hypochondriacus_439                ..GTEDV....GKE.....WIG.SCAEEVKK.HAF.DP..SLQFINRSEFPMESNWKIFCDN
BAF93187|Amaranthus_tricolor                                  ..GTEDV....GKE.....WIG.SCAEEVKK.HAF.DP..SLQFIHRSEFPMESNWKVFCDN
XP_012828802|Erythranthe_guttata__spotted_monkey_             ..LDNKS..V.SHE.....WLG.SAAEILSA.NGI.DS..SLDYVCRRVYTLECNWKVFCDN
Migut.J00779.1|Mimulus_guttatus                               ..LDNKS..V.SHE.....WLG.SAAEILSA.NGI.DS..SLDYVCRRVYTLECNWKVFCDN
EYU18199|Erythranthe_guttata__spotted_monkey_flow             ..LDNKD..V.SSE.....WLG.NAAELLTA.NGI.DT..SLDYVRRRVYTLDCNWKVFCDN
KQ992415.1|Dorcoceras_hygrometricum_corrected                 ..VDNEA..V.GNE.....WLG.STADILSN.NGV.DT..SLDYICRRTYTIECNWKVFCDN
XP_011094129|Sesamum_indicum__sesame_                         ..LDTNG..V.AHD.....WLG.STAEILSS.NGI.DA..SLVYLCRRVYTLECNWKVFCDN
XP_015080536|Solanum_pennellii__Lycopersicon_penn             ..SDFDL..V.GNE.....WLG.SSSQILAD.GGV.DA..SLSFLCRREYAIECNWKVFCDN
XP_004242785|Solanum_lycopersicum__Lycopersicon_e             ..SDFDL..V.GNE.....WLG.SSSQILAD.GGV.DA..SLSFLCRREYAIDCNWKVFCDN
NP_001275051|Solanum_tuberosum__potato_                       ..SDFDF..V.GNE.....WLG.SSSQILAD.GGV.DS..SLSFLCRREYTIECNWKVFCDN
XP_016556147|Capsicum_annuum                                  ..SAFDL..A.GNE.....WLG.SSSQILAD.GGV.DS..SLSFVSRREYTIECNWKVFCDN
ACR15118|Lycium_barbarum                                      ..SDFDL..V.GNE.....WLG.SSSQILAD.GGV.DS..SLSFLCGREYTVECNWKVFCDN
XP_009757252|Nicotiana_sylvestris__wood_tobacco_              ..SDFDL..V.GNE.....WLG.TSSQILAD.GGV.DS..SLSFLCRREYTIECNWKVFCDN
XP_016460838|Nicotiana_tabacum_(common_tobacco)               ..SDFDL..V.GNE.....WLG.TSSQILAD.GGV.DS..SLSFLCRREYTIECNWKVFCDN
OIT26339|Nicotiana_attenuata                                  ..SDFDL..V.GNE.....WLG.TSSQILAD.GGV.DS..SLSFLCRREYTIECNWKVFCDN
XP_009601434|Nicotiana_tomentosiformis                        ..SDFDL..V.GNE.....WLG.TSSQILVDGGGV.DS..SLSFLCRREYTIECNWKVFCDN
XP_016508540|Nicotiana_tabacum_(common_tobacco)               ..SDFDL..V.GNE.....WLG.TSSQILVDGGGV.DS..SLSFLCRREYTIECNWKVFCDN
XP_015081828|Solanum_pennellii__Lycopersicon_penn             ..ADIDL..V.GNE.....WLG.NSSQILTD.GGV.DS..SLSFLCRREYTVECNWKVYCDN
XP_004243034|Solanum_lycopersicum__Lycopersicon_e             ..ADFDL..V.GNE.....WLG.NSSQILID.GGV.DS..SLSFLCRREYTVECNWKVYCDN
CDP08949|Coffea_canephora                                     ..SQSNI..V.GDE.....WLG.SSSELLST.RIN.DS..SLKFLCRHEYTINCNWKVFCDN
XP_019182381|Ipomoea_nil__Japanese_morning_glory_             ..SETVP..V.GNE.....WLG.SSSEILIA.NGI.DS..SLSYVCRREYTIECNWKVFCDN
XP_010433050|Camelina_sativa__false_flax_                     ..ESHDA.LV.ASE.....WLG.TSVGKLSE.GGV.DS..PLSFICRREYTMDCNWKVFCDN
XP_010436340|Camelina_sativa_false_flax_corr                  ..ESHDA.LV.ASE.....WLG.TSVGKLSE.GGV.DS..PLSFICRREYTMDCNWKVFCDN
XP_010438247|Camelina_sativa__false_flax_                     ..ESDDA.LV.ASE.....WLG.TSVGRLSE.GGV.DS..PLSFICRREYTMDCNWKVFCDN
XP_010447794|Camelina_sativa__false_flax_                     ..ESDDA.LV.ASE.....WLG.TSVGRLSE.GGV.DS..PLSFICRREYTMECNWKVFCDN
XP_006285614|Capsella_rubella                                 ..ES.DA.LV.ASE.....WLG.TSVGRLSE.GGV.VS..PLSFICRREYTMDCNWKVFCDN
Cagra.0268s0015.1|Capsella_grandiflora                        ..EC.DA.LV.GSE.....WLG.TSMGRLSE.GGV.DS..PLSFICRREYTMDCNWKVFCDN
Bostr.7867s0836.1|Boechera_stricta                            ..ET.DG.LV.ASE.....WLG.TSVGRLSH.GGV.DS..PLSFICRREYTMDCNWKVFCDN
NP_194718|Arabidopsis_thaliana__thale_cress_                  ..ET.DE.LV.ASE.....WLG.TSVGRLSQ.GGV.DS..PLSYICRREYTIDCNWKVFCDN
XP_002869406|Arabidopsis_lyrata_subsp._lyrata                 ..ET.DG.LV.ASE.....WLG.TSVGRLSQ.GGV.DS..PLSYICRREYTIDCNWKVFCDN
Araha.6052s0002.1|Arabidopsis_halleri                         ..ET.NG.LV.ASE.....WLG.TSVGRLSQ.GGV.DS..PLSYICRREYTIHCNWKVFCDN
XP_006412796|Eutrema_salsugineum                              ..ES.DG.LV.ASE.....WLG.TCVGKLSQ.GGV.DS..SLTFICRREYTIDCNWKVFCDN
KFK29548|Arabis_alpina__gray_rockcress_                       ..ET.DG.VV.ETE.....WLG.TSAGRLSQ.GGV.DS..QLSFICRREYTIDCNWKVFCDN
KFK40841|Arabis_alpina__gray_rockcress_                       ..ET.DG.LV.ETE.....WLG.TSAGRLSQ.GGV.DS..QLSFICRREYTIDCNWKVFCDN
XP_018481725|Raphanus_sativus__radish_                        ..VESDGSVVVASE.....WLG.SSVGRLSE.GGVDDS..TLGFICRREYTIDCNWKVFCDN
XP_018435688|Raphanus_sativus__radish_                        ..VESDGSVVEASE.....WLG.SSVGRLSE.SGV.DDAFTLSFICRREYTIDCNWKVFCDN
XP_013705395|Brassica_napus__rape_                            ..VESDG.LV.ASE.....WLG.SSSGRLSE.GGV.DS..HLSFICRREYTIDCNWKVFCDN
XP_013596413|Brassica_oleracea_var._oleracea                  ..VESDG.LV.ASE.....WLG.SSAGRLSE.GGV.DS..HLSFICRREYTIDCNWKVFCDN
XP_009137872|Brassica_rapa__field_mustard_                    ..VESDG.LV.ASE.....WLG.SSVGRLSE.GGV.DS..TFSFICRREYTIDCNWKVFCDN
XP_013738248|Brassica_napus__rape_                            ..IESDGSVV.ASE.....WLG.SSVGRLSE.GGV.DS..TFSFICRREYTIDCNWKVFCDN
XP_010541096|Tarenaya_hassleriana                             ..RNDDA..V.AKD.....WLG.SSANILSQ.GGV.DD..SLSYICRREYTIDCNWKVFCDN
Kaladp0809s0111.1|Kalanchoe_fedtschenkoi                      ..TDEST..V.EDE.....WLG.DCSEIQTI.KGV.DS..SLRFICRRSYTIECNWKVFVDN
Kalax.0333s0026.1|Kalanchoe_laxiflora_corr_462aa              ..TDEST..V.EDE.....WLG.DCSEIQTI.KGE.DS..SLRFICRRSYTIECNWKVFVDN
Kalax.0012s0114.1|Kalanchoe_laxiflora_corr_462aa              ..TDEST..V.EDE.....WLG.DCSEIQTI.KEV.ES..SLRFICRRSYTIECNWKVFVDN
XP_010667950|Beta_vulgaris_subsp._vulgaris__sugar             ..VDCGS..L.END.....WLG.SSAGLLSA.NGV.DN..SLSYICRHEYTLECNWKVFCDN
AH021158-RA|Amaranthus_hypochondriacus_421aa-corr             ..VDYAT..L.ENQ.....WLG.SSAGFLSA.NGV.DT..SLSYICRREYTIECNWKVFCDN
XP_021866412|426|Spinacia_oleracea_(spinach)                  ..VDYGN..M.END.....WLG.GSADLLSI.NGV.DT..SLSYICRREYTLECNWKVFCDN
BAG74777|Hordeum_vulgare_subsp._vulgare__domestic             ..TVHDV..V.GDE.....WLG.SASDLLSR.SGI.NT..SLPHICRREYIIECNWKVFCDN
AKZ66518|Triticum_monococcum_subsp._aegilopoides              ..TVDDV..V.GDE.....WLG.SASDLLSR.SGI.NT..SLPHIE................
FAOM01578495|Triticum_aestivum_UpdateAMA02065                 ..TVGDV..V.GDE.....WLG.SASDLLSR.SGI.NT..SLPHICRREYTIECNWKVFCDN
EMT07994|Aegilops_tauschii_                                   ..TVDDV..V.GDE.....WLG.SASDLLSR.SGI.DT..SLPHICRREYIIECNWKVFCDN
ACZ92178|Triticum_turgidum_subsp._durum__durum_wh             ..............................................................
KD266899|Triticum_urartu_UpdateEMS46986                       ..TVDDV..VGGDE.....WLG.SASDLLSR.SGI.DT..SLPHICRREYIIECNWKVFCDN
ABV64740|Leymus_chinensis                                     ..TVDDV..V.GDE.....WLG.SASDLLSR.SGI.NT..SLPHICRREYIIECNWKVFCDN
Brast07G193900.1|Brachypodium_stacei                          ..SV.DV..V.GDE.....WLG.SASDLLSR.NGI.DT..SLPHICRREYIIECNWKVFCDN
XP_003563491|Brachypodium_distachyon__stiff_brome             ..TVDDV..V.GDE.....WLG.SASDLLSR.NGI.DT..SLPHICRREYIIECNWKVFCDN
XP_006657294|Oryza_brachyantha__malo_sina_                    ..TADDT..V.GDE.....WLG.SASDLLSR.NGI.DT..SLPHVCRREYIIECNWKVFCDN
XP_015643170|Oryza_sativa_Japonica_Group__Japanes             ..TADNT..V.GDE.....WLG.SASDLLSR.NGI.DT..SLPHICRREYIIECNWKVFCDN
EEC81252|Oryza_sativa_Indica_Group__long-grained_             ..TADNT..V.GDE.....WLG.SASDLLSR.NGI.DT..SLPHICRREYIIECNWKVFCDN
XP_002437525|Sorghum_bicolor__sorghum_                        ..NVYDA..V.GNE.....WLG.SASDLLGT.NGI.DT..SLPHICRREYIINCNWKVFCDN
NP_001105926|Zea_mays                                         ..NVYDT..V.GNE.....WLG.SASDLLGT.NGI.DT..SLPHICRREYIINCNWKVFCDN
Pavir.Db00260.1|Panicum_virgatum_CORR_316f                    ..DVGDV..V.GDE.....WLG.SASKLLST.NGI.DT..SLPHICRREYIINCNWKVFCDN
LWDX02005496.1|Dichanthelium_oligosanthes_update_             ..DVGDV..V.GDE.....WLG.SASSLLST.NGI.DN..SLPHICRREYIINCNWKVFCDN
Sevir.4G257300.1|Setaria_viridis                              ..DSGDV..V.GNE.....WLG.SASELLST.NGI.DT..SLPHICRREYIINCNWKVFCDN
XP_004966128|Setaria_italica__foxtail_millet_                 ..DAGDV..V.GDE.....WLG.SASELLST.NGI.DT..SLPHICRREYIINCNWKVFCDN
Oropetium_20150105_16264A|Oropetium_thomaeum                  ..DVDDI..V.GDE.....WLG.SASDLLST.NGI.DT..SLPHICRREYIINCNWKVFCDN
Pahal.D00004.1|Panicum_hallii                                 ..DVGDV..V.GDE.....WLG.SASKLLST.NGI.DT..SLPHICRREYIINCNWKVFCDN
Pavir.J39646.1|Panicum_virgatum_Corr_363f                     ..DAGDV..V.GDE.....WLG.SASKLLST.NGI.GT..SLPHICRREYIINCNWKVFCDN
OAY65202|Ananas_comosus__pineapple_                           ..ITEDI..V.GKE.....WLG.SASNLLST.NGI.DT..SLKHVSRREYTIECNWKVFCDN
XP_009410214|Musa_acuminata_subsp._malaccensis__w             ..CCTKT..V.ENE.....WLG.SASNILST.NGI.DS..SLKHVCRREYTVQCNWKVFCDN
XP_010927268|Elaeis_guineensis__African_oil_palm_             ..FANDL..V.PNE.....WLG.SASDTLSN.DGI.DS..SLKHICRREYTIRCNWKVFCDN
XP_008801514|Phoenix_dactylifera__date_palm_                  ..FANDL..V.HNE.....WLG.SASDIISN.DGI.DS..SLKHVCRREYTIRCNWKVFCDN
KMZ72255|Zostera_marina                                       ..KSIDI..V.GNQ.....WLG.DCSEMLSM.NKI.DT..NLKHICRREYTIKCNWKVFCDN
JAT51600|Anthurium_amnicola                                   ..QQNVSNEIVANE.....WLG.SCSEILR..DTI.DS..SLKHLCRREYTLECNWKVFCDN
Spipo5G0003400|Spirodela_polyrhiza                            ..SGETV....EAE.....WLG.SSSEMLR..SGV.DS..SLKHVCRREYAIECNWKVFCDN
XP_006845775|Amborella_trichopoda                             ..NCKRV....AEE.....WLG.SCSEILST.NGV.DL..SLPHVARREYSINCNWKVFCDN
AFP19450|Camellia_sinensis                                    ..QAADVNNI.GKE.....WLG.SSSEILST.NGV.DS..SLSYICRREYTIECNWKVFCDN
XP_017254907|Daucus_carota_subsp._sativus                     ..SLDNV....GTE.....WLG.SASEILSV.NGV.DS..SLSYLCRQEYIIECNWKVFCDN
ABX57826|Chrysanthemum_lavandulifolium                        ..DDDDV....GME.....WLG.STSDILST.NGV.DT..SLSYLCRREYTIECNWKVFCDN
LEKV01000036.1|Cynara_cardunculus_update_KVI11819             ..CDDNV....GME.....WLG.STSEILST.NGV.DT..SLSYLRRREYTIECNWKVFCDN
XP_008443631|Cucumis_melo__muskmelon_                         ..VDEDK..V.ARE.....WLG.TCADVLRL.NGV.DA..SLSYVCRREYTIDCNWKVFCDN
XP_004139149|Cucumis_sativus__cucumber_                       ..VDEDK..V.ARE.....WLG.TCADVLRL.NGV.DA..SLSYVCRREYTIECNWKVFCDN
XP_008351406|Malus_domestica__apple_                          ..SDPGS..V.QNE.....WLG.SSAEVLSN.NGI.DT..SLSFVCRRDYVIECNWKVFCDN
XP_008391562|Malus_domestica__apple_                          ..SDPGS..V.QNE.....WLG.SSAEVLSN.NGI.DT..SLSFVCRRDYVIECNWKVFCDN
XP_009343851|Pyrus_x_bretschneideri__Chinese_whit             ..SDPGS..V.QNE.....WLG.SSAEVLSN.NGI.DT..SLSFVCRRDYVIECNWKVFCDN
AER10510|Pyrus_betulifolia_corr_405aa                         ..SDPGS..V.QNE.....WLG.SSAEVLSN.NGI.DT..SLSFVCRRDYVIECNWKVFCDN
XP_008223853|Prunus_mume__Japanese_apricot_                   ..SDTDI..V.EKE.....WLG.SSSELLSN.NGV.DS..SLSFVCRRDYMIECNWKVFCDN
ONI27175|Prunus_persica__peach_                               ..SDTDI..V.EKE.....WLG.SSSELLSN.NGV.DS..SLSFVCRRDYMIECNWKVFCDN
```

```
XP_004301419|Fragaria_vesca_subsp._vesca          ..SDVEI..V.ESK.....WLG.TSSDILSK.NGV.DS..SLTFVCRREYMIECNWKVFCDN
XP_015889685|Ziziphus_jujuba__common_jujube_      ..SDEDI..V.AEE.....WLG.SSSELLSS.NGV.DS..SLSYVCRREYTIECNWKVFCDN
NW_010360102.1|Morus_notabilis_corrected          ..SDVDD..V.ANE.....WLG.SASEVLNT.NGV.DS..SLSYICRREYTIECNWKVFCDN
Lus10032689|Linum_usitatissimum                   ..VDDDN.LV.ANE.....WLG.STSEILKS.GGV.DA..SLSYLFRREYTIECNWKVFCDN
Lus10008571|Linum_usitatissimum_corrected         ..VDDDN.LV.ANE.....WLG.STSEILKS.GGV.DA..SLSYLFRREYTIECNWKVFCDN
XP_011020077|Populus_euphratica__Euphrates_poplar ..AD.NT..V.GSA.....WLG.SCSEFLGA.NGV.DS..SLSYLCRRVYDIECNWKVFCDN
XP_002308100|Populus_trichocarpa__Populus_balsami ..AD.NT..V.GSE.....WLG.SCSEYLAA.NGV.DS..SLSYLCRRVYDIECNWKVFCDN
SapurV1A.0198s0060.1|Salix_purpurea               ..GD.YT..V.GNE.....WLG.SCSEFLGA.NGV.DS..SLSYLCRRVYDIECNWKVFCDN
OAY46667|Manihot_esculenta__cassava_              ..VDGNM..V.ESE.....WLG.SCSDALKT.NGV.DS..SLSYVCRRVYDIECNWKVFCDN
XP_002518256|Ricinus_communis__castor_bean_       ..VDGNM..V.ENE.....WLG.SCSGLLKT.NGV.DS..SLSYVCRRTYNIECNWKVFCDN
XP_012074438|Jatropha_curcas                      ..VEGSM..V.ENE.....WLG.SCSEILKN.GGV.DS..SLNHVCRRVYNMECNWKVFCDN
XP_010046909|Eucalyptus_grandis                   ..VESDI..A.AKE.....WTG.SCVDILGN.NCI.DP..SLTYLCRREYILECNWKVFCDN
ABS71853|Eucalyptus_camaldulensis_Murray_red_gum_ ..VESDI..A.AKE.....WTG.SCVDILGN.NCI.DP..SLTYLCRREYILECNWKVFCDN
XP_006474292|Citrus_sinensis__sweet_orange_       ..VDSNV..V.ANE.....WLG.GSSEILSI.NGI.DS..SLSYLCRREYTIECNWKVFCDN
XP_006453220|Citrus_clementina_reviewed           ..VDSNV..V.ANE.....WLG.GSSEILSI.NGI.DS..SLSYLCRREYTIECNWKVSCDN
XP_017612508|Gossypium_arboreum                   ..IDTDN..V.ASE.....WLG.SSSELFSL.DGV.DT..TLTYVCRREYIIECNWKVFCDN
XP_016686114|Gossypium_hirsutum__cotton_          ..IDTDN..V.ASE.....WLG.SSSELFSL.NGV.DT..TLTYVCRREYIIECNWKVFCDN
XP_012459381|Gossypium_raimondii                  ..IDTDN..V.ASE.....WLG.SSSELFSL.NGV.DT..TLTYVCRREYIIECNWKVFCDN
XP_016719040|Gossypium_hirsutum__cotton_          ..IDTDN..V.ASE.....WLG.SSSELFSL.NGV.DT..TLTYVCRREYIIECNWKVFCDN
EOY05290|Theobroma_cacao__cacao_                  ..IDTDI..L.ASE.....WLG.SCSEILSL.NGV.DS..PLTYVCRREYIIECNWKVFCDN
AWWV01015497-AWWV01004954_Corchorus_capsularis_39 ..IDTDS..I.ASE.....WLG.SCSKILSL.NGF.DS..QLTYVCRREYIIECNWKVFCDN
OMO51681|Corchorus_olitorius                      ..IDTDS..I.ASE.....WLG.SCSKILSL.NGF.DS..QLTYVCRREYIIECNWKVFCDN
GAV59711|Cephalotus_follicularis                  ..VDSNI..V.ANE.....WLG.TCSEILSL.NGV.DS..SLSYVCRREYTLECNWKVFCDN
XP_018807260|Juglans_regia__English_walnut_       ..ADSDT..V.ADE.....WLG.SCSEILST.NGV.DS..LLSYVCRREYTIECNWKVFCDN
XP_019078540|Vitis_vinifera__wine_grape_          ..ADSKI..V.GKE.....WLG.SSSDILSN.GGI.DT..SLSYVCRREYTIECNWKVFCDN
Aqcoe7G261800.1|Aquilegia_coerulea                ..VHNKI..V.ENE.....WLG.TCSELLTT.NGI.DT..SLSFVCRREYTIDCNWKVFCDN
XP_010276721|Nelumbo_nucifera__sacred_lotus_      ..ADSKM..V.ENE.....WLG.SCSDILST.NGV.DS..SLSYVCRREYIIECNWKVFCDN
XP_016201893|Arachis_ipaensis                     ..IDSTY..V.AKE.....WLG.SCSEILST.NGI.DS..SLNYVCRREYTIDCNWKVFCDN
XP_015973237|Arachis_duranensis                   ..IDSTY..V.AKE.....WLG.SCSEILST.NGI.DS..SLNYVCRREYTIDCNWKVFCDN
Tp57577_TGAC_v2_mRNA5577|Trifolium_pratense       ..VDSHN..V.AKE.....WLG.SCSEILST.NGV.DS..LLSYVCRREYTLQCNWKVFCDN
GAU38011|Trifolium_subterraneum                   ..VDSHN..L.AKE.....WLG.SCSEILST.NGV.DS..SLSYVCRREYTIQCNWKVFCDN
XP_003610028|Medicago_truncatula__barrel_medic_   ..VDNHN..V.AEE.....WLG.SCTELLST.NGV.DS..SLSYVCRREYTIECNWKVFCDN
XP_004507911|Cicer_arietinum__chickpea_           ..VDSHN..V.AKE.....WLG.SCSELLST.NGV.DS..SLSYLCRREYTIECNWKVFCDN
XP_019463425|Lupinus_angustifolius__narrow-leaved ..VDSHN..V.ASE.....WLG.SCSEILST.NGV.DS..SLSYVCRREYTIECNWKVFCDN
KYP52554|Cajanus_cajan__pigeon_pea_               ..VDIHG..V.SKE.....WLG.SCSEFLST.NGV.DS..SLSYVCRREYTVECNWKVFCDN
XP_003549280|Glycine_max__soybean_                ..VDSHN..V.SKE.....WLG.SSSEILST.NGV.DS..SLSYVCRREYTIECNWKVFCDN
XP_007134857|Phaseolus_vulgaris_CHR1_corr_208aaf  ..VDSHN..V.SKE.....WLD.SCSEVLSS.TGI.DS..SLSYVCRREYTIECNWKV....
XP_007154695|Phaseolus_vulgaris_CORR              ..ADSHN..V.SKE.....WLG.SCSEVLSS.SGI.DS..SLSYVCRREYTIECNWKVFCDN
XP_017410379|Vigna_angularis__adzuki_bean_        ..VDSHN..V.SKA.....WLG.SCSEVLSS.SGI.DS..SLSYVCRREYTIECNWKVFCDN
XP_014508581|Vigna_radiata_var._radiata__mung_bea ..VDSHN..M.SKE.....WLG.SCSEVLSS.SGI.DS..SLSYVCRREYTIECNWKVFCDN
AUSU01000986.1|Genlisea_aurea_corrected-f         ..RNVHH..P.GHE.....WLG.PTAHRVLASTGITDA..SLHFICRRVYTLECNWKVFCDN
OAE24413|CORR_Marchantia_polymorpha               ..SEPDSVAV.EKQ.....WLG.SAAKTLKG.SNI.DS..SLLHVGKREYTINCNWKVFCDN
Sphfalx0064s0059.1|Sphagnum                       ..VSTTV....GEE.....WLG.SAVSVLST.AGV.DT..SLHHVATREYLINCNWKVYCDN
XP_001752587|Physcomitrella_patens                ..ASSDV....EEG.....WLG.SAGPTLSS.AGI.DS..TMQHVATREYVIKCNWKVYCDN
XP_002508933|Micromonas_sp._RCC299                ..EEEEEEEAPIEA.....WMGPDVAAKISA.SWS.DDPKSMRFVARREYRLRCNWKVFADN
XP_003061830|Micromonas_pusilla_CCMP1545          ..........VNE.....WLG.EAGERMRS.AGVAE....MTHVARREYELNCNWKVFCDN
XP_005645368|Coccomyxa                            ..........SFEA.....QLG.PEGLQAMEAAGIADS..HLVHVASQSYTLQCNWKVFCDN
ADIC01002027.1|Chlorella_variabilis_corrected_pre QQEQQQQQQAGQEEGVAAWLG.PGGSAAALAAGIADS...LVLVASREYEIACNWKVFIDN
XP_013903967|Monoraphidium_neglectum_rev          ...VSSSAPPPLEE.....WLG.SAGERMSA.LGL.AE..PMVHVAQRTYELRCNWKVFCDN
XP_005644270|Coccomyxa_subellipsoidea_C-169       .............D.....WLG.EGGRKMEA.CDMKAP..DLVHVRTVPYDLDCNWKVFNDN
NC_024001.1|Bathycoccus_prasinos_corrected        ..........LRT.....FLGADFASRVEEADINVSDRESFRHVARETFEVESNWKVFIDN
EWM23896|Nannochloropsis_gaditana                 .PHVVGKDGSHPLA.....YLE.PLLERLDAEHGF.SS..GLRHVRRRRYRVQCNHKVVSDN
XP_002738379|Saccoglossus_kowalevskii             ..........FFE.....DLE.SLKNRLDG.IGFSS...GMKFMKRITYTLNCNWKVFVDN
XP_006820984|Saccoglossus_kowalevskii             ..........FHD.....EVA.AIENCMKGFEFNE....SWCFVKRVTYPLKCNWKVVCDN
XP_002737044|Saccoglossus_kowalevskii             ..........FKQ......LA.SYEAILQQ.FGYDINKNEIKFFRRKSFPIKSNWKAIIDN
XP_002597329|Branchiostoma_floridae__Florida_lanc ..........FEE.....KTS.PLLQHLDT.QGFTH...GMRHICRRIYDAKCNWKLLVEN
XP_019618440|Branchiostoma_belcheri__Belcher's_la ..........FQE.....KTS.PLLHHLDS.QGFTQ...GMTHICRRYYDTRCNWKLLVEN
XP_019644608|Branchiostoma_belcheri__Belcher's_la ..........FDQ.....KVG.PLAQELEK.MGFTS...GMRHICRRQYQLRCNWKLIVDN
XP_019644788|Branchiostoma_belcheri__Belcher's_la ..........FDQ.....KVG.PLAQELEE.MGFTR...GMRHICRRQYQLRCNWKLIVDN
XP_002610312|Branchiostoma_floridae__Florida_lanc ..........YDQ.....KVL.PLMLELEE.MGFSS...GMRHVCRRQYQLNCNWKLIVDN
XP_002599795|Branchiostoma_floridae__Florida_lanc ..........FET.....QVM.SLKAQLDE.LGFTS...GLRFVERRRYHVNCNWKVVLDN
XP_019633636|Branchiostoma_belcheri__Belcher's_la ..........FET.....QVT.SLKDQLDD.LGFTS...GLRFVERRRYRVNCNWKVVLDN
XP_004333794|Acanthamoeba_castellanii_str._Neff   ..........LHE.....ELK.PLEDVLES.TQYR....KLEYMCTRNYKVKSNWKVYVDN
GAQ84770|Klebsormidium_flaccidum                  PTSNGTADSRAVQE.....WVG.EAAGLLTH.HRVISG..EYKYMKRLEYDIGCNWKAYIDN
```

```
                                                     290        300            310          320
BAC77698|Atriplex_nummularia                   YLDSSYHVPYAHKYYAT.ELDFD.......TYQTDM..VGNVTIQRV......AGTS.....
AGC13074|Atriplex_semibaccata                  YLDSSYHVPYAHKYYAT.ELDFD.......TYQADM..VGNVTIQRV......AGTS.....
AHH24260|Atriplex_amnicola                     YLDSSYHVPYAHKYYAT.ELDFD.......TYQTDM..VGNVTIQRV......AGTS.....
AFG28558|Atriplex_canescens__four-winged_saltbush YLDSSYHVPYAHKYYAT.ELDFD.......TYQTDM..VGNVTIQRV......AGTS.....
Q9LKN0|Atriplex_hortensis                      YLDSSYHVPYAHKYYAT.ELDFD.......TYQTDM..VGNVTIQRV......AGTS.....
AAL92561|Atriplex_prostrata                    YLDSSYHVPYAHKYYAT.ELDFD.......TYQTDM..VGNVTIQRV......AGTS.....
O04121|Spinacia_oleracea__spinach_             YLDSSYHVPYAHKYYAT.ELNFD.......TYDTQM..IENVTIQRV......EGSS...NK
ABG34274|Ophiopogon_japonicus                  YLDSSYHVPYAHKYYAT.ELNFD.......TYDTQM..IENVTIQRV......EGSS...NK
AEW31326|Haloxylon_persicum                    YLDSSYHVPYAHKYYAT.ELDFD.......TYDTQM..IEKVVIQRV......GSSS...NK
ACX47904|Haloxylon_ammodendron                 YLDSSYHVPYAHKYYAT.ELDFD.......TYDTQM..IEKVVIQRV......GSSS...NK
AJF98574|Salicornia_bigelovii                  YVDSSYHVPYAHKYYAT.ELDFD.......TYDTQT..IGKVVIQRV......AGNS...NK
AAV91779|Salicornia_europaea                   YVDSSYHVPYAHKYYAT.ELDFD.......TYDTQT..IGKVVIQRV......AGNS...NK
AFW04225|Suaeda_maritima                       YLDSSYHVPYAHKYYAT.ELDFD.......TYDTQT..IGKVVIQRV......GSNT...NR
AAM43920|Suaeda_liaotungensis                  YLDSSYHVPYAHKYYAT.ELDFD.......TYDTQT..IGKVVIQRV......GSNT...NR
XP_010682183|Beta_vulgaris_subsp._vulgaris__sugar YLDSSYHVPYAHKYYAA.ELDFD.......TYNTEM..IEKCVIQRV......GSSS...NK
AHYPO_002206-RA|Amaranthus_hypochondriacus_439 YLDSAYHVPYAHKYYAA.ELDFD.......TYKTDL..LEKVVIQRV......ASSS...NN
BAF93187|Amaranthus_tricolor                   YLDSAYHVPYAHKYYAA.ELDFD.......TYKTDL..LEKVVIQRV......ASSS...NK
XP_012828802|Erythranthe_guttata__spotted_monkey_ YLDGGYHVPYAHKGLAS.SLKLG.......SYSTEM..YEKVSIQSC......GGNK...TG
Migut.J00779.1|Mimulus_guttatus                YLDGGYHVPYAHKGLAS.SLKLG.......SYSTEM..YEKVSIQSC......GGNK...TG
EYU18199|Erythranthe_guttata__spotted_monkey_flow FLDGGYHVPYVHRGFAS.SLKLD.......SYSTEV..YEKVSVQRC......RVNN...TN
KQ992415.1|Dorcoceras_hygrometricum_corrected  YLDGGYH......................M..YEKVSIQSC......EGCA...AD
XP_011094129|Sesamum_indicum__sesame_          YLDGGYHVPYAHRGLAS.GLQLD.......SYSTEM..YEKVSIQTC......GGST...KA
XP_015080536|Solanum_pennellii__Lycopersicon_penn YLDGGYHVPYAHKGLAS.GLTLD.......SYSTTI..FEKVSIQRC......ETGS...AG
XP_004242785|Solanum_lycopersicum__Lycopersicon_e YLDGGYHVPYAHKGLAS.GLTLE.......SYSTTI..FEKVSIQRC......ETGS...AG
NP_001275051|Solanum_tuberosum__potato_        YLDGGYHVPYAHKGLAS.GLTLD.......SYSTTI..FEKVSIQRC......ETGS...AE
XP_016556147|Capsicum_annuum                   YLDGGYHVPYAHKGLAS.GLTLD.......SYATTI..LEKVSIQRC......ETGS...AE
ACR15118|Lycium_barbarum                       YLDGGYHVPYAHKDLAS.GLTLD.......SYSTTI..LEKVSIQRC......ETGS..AER
XP_009757252|Nicotiana_sylvestris__wood_tobacco_ YLDGGYHVPYAHKSLAS.GLTLD.......SYSTTI..LEKVSIQRC......ETGS...AE
XP_016460838|Nicotiana_tabacum_(common_tobacco) YLDGGYHVPYAHKSLAS.GLTLD.......SYSTTI..LEKVSIQRC......ETGS...AE
OIT26339|Nicotiana_attenuata                   YLDGGYHVPYAHKGLAS.GLTLD.......SYSTTI..LEKVSIQRC......ETGS...AE
XP_009601434|Nicotiana_tomentosiformis         YLDGGYHVPYAHKGLAS.GLTLD.......SYSTTI..LEKVSIQRC......ETGS...AE
XP_016508540|Nicotiana_tabacum_(common_tobacco) YLDGGYHVPYAHKGLAS.GLTLD.......SYSTTI..LEKVSIQRC......ETGS...AE
XP_015081828|Solanum_pennellii__Lycopersicon_penn YLDGGYHVPYVHKSYAS.VLKLD.......SYSTTI..LEKVSIQRC......D......AE
XP_004243034|Solanum_lycopersicum__Lycopersicon_e YLDGGYHVPYVHKSYAS.VLKLD.......SYSTTI..LEKVSIQRC......D......AE
CDP08949|Coffea_canephora                      YLDGGYHVPYAHKGLAS.GLKLE.......SYSSIV..YEKVSIQKC......DGDA...TE
XP_019182381|Ipomoea_nil__Japanese_morning_glory_ YLDGGYHVPFAHKSLAS.DLKLE.......TYSTEI..FERVSIQQC......LGGT...TE
XP_010433050|Camelina_sativa__false_flax_      YLDGGYHVPYAHKGLMS.GLNLE.......TYSTTI..FEKVSIQEC......GGGSK..AG
XP_010436340|Camelina_sativa_false_flax_corr   YLDGGYHVPYAHKGLMS.GLNLE.......TYSTTI..FEKVSIQEC......GGGSK..AG
XP_010438247|Camelina_sativa__false_flax_      YLDGGYHVPYAHKGLMS.GLNLE.......TYSTTI..FEKVSIQEC......GGGSK..AG
XP_010447794|Camelina_sativa__false_flax_      YLDGGYHVPYAHKGLMS.GLNLE.......TYSTTI..FEKVSIQEC......GGGSK..AG
XP_006285614|Capsella_rubella                  FLDGGYHVPYAHKGLMS.GLNLE.......TYSTTI..FERVSIQEC......GGGSK..AG
Cagra.0268s0015.1|Capsella_grandiflora         YLDGGYHVPYAHKGLMS.GLNLE.......TYSTTI..FERVSIQEC......GGGSK..AG
Bostr.7867s0836.1|Boechera_stricta             YLDGGYHVPYAHKGLMS.GLNLE.......TYSTTI..FERVSIQEC......GGGSK..AG
NP_194718|Arabidopsis_thaliana__thale_cress_   YLDGGYHVPYAHKGLMS.GLDLE.......TYSTTI..FEKVSIQEC......GGGSK..VG
XP_002869406|Arabidopsis_lyrata_subsp._lyrata  YLDGGYHVPYAHKGLMS.GLDLE.......TYSTTI..FEKVSIQEC......GGGSK..VG
Araha.6052s0002.1|Arabidopsis_halleri          YLDGGYHVPYAHKGLMS.GLDLE.......TYSTTI..FEKVSIQEC......GGGSK..IG
XP_006412796|Eutrema_salsugineum               YLDGGYHVPYAHKGLMS.GLDLE.......TYSTTI..FERVSIQEC......GGGSK.AGE
KFK29548|Arabis_alpina__gray_rockcress_        YLDGGYHVPYAHKGLMS.GLNLE.......SYSTTI..FERVSIQEC......GGNSK.AGE
KFK40841|Arabis_alpina__gray_rockcress_        YLDGGYHVPYAHKGLIS.GLNLE.......SYST............................
XP_018481725|Raphanus_sativus__radish_         YLDGGYHVPYAHKGLMS.GLDLD.......TYSTTL..FERVSIQAC...GGGGGGSK..AG
XP_018435688|Raphanus_sativus__radish_         YLDGGYHVPYAHKGLMS.GLDLD.......SYSTTL..FERVSIQACGGGGGGGGGSK...A
XP_013705395|Brassica_napus__rape_             YLDGGYHVPYAHKGLMS.GLDLE.......TYSTTL..FERVSIQEC.....GGGGSK.AGE
XP_013596413|Brassica_oleracea_var._oleracea   YLDGGYHVPYAHKGLMS.GLDLE.......TYSTTL..FERVSIQEC.....GGGGSK.AGE
XP_009137872|Brassica_rapa__field_mustard_     YLDGGYHVPYAHKGLMS.GLDLE.......TYSTTL..FERVSIQEC.....GGGGSKAGEE
XP_013738248|Brassica_napus__rape_             YLDGGYHVPYAHKGLMS.GLDLE.......TYSTTL..FERVSIQEC.....GGGGSKAGEE
XP_010541096|Tarenaya_hassleriana              YLDGGYHVPYAHKGLAS.GLDLN.......SYSTTM..LERVSIQEC......GGGSK..AG
Kaladp0809s0111.1|Kalanchoe_fedtschenkoi       YLDGGYHVPYAHKGLAS.GLDLD.......SYSTTI..YEKLSIQKC......GGGPT...AN
Kalax.0333s0026.1|Kalanchoe_laxiflora_corr_462aa YLDGGYHVPYAHKGLAS.GLDLD.......SYSTTI..YEKLSIQKC......GGGPT...AN
Kalax.0012s0114.1|Kalanchoe_laxiflora_corr_462aa YLDGGYHVPYAHKGLAS.GLDLD.......SYSTTI..YEKLSIQKC......GGGPT...AN
XP_010667950|Beta_vulgaris_subsp._vulgaris__sugar YLDGGYHVPYAHKNLAS.GLNLD.......SYSTEM..YEKVSIQRC......ASSST...EN
AH021158-RA|Amaranthus_hypochondriacus_421aa-corr YLDGGYHVPCAHKELAS.GLNLD.......TYSTEI..FEKVSIQRC......TSSST...ET
XP_021866412|426|Spinacia_oleracea_(spinach)   YLDGGYHVPYAHKNLAS.GLNLD.......SYSTEM..FEKVSIQRC......ASSST...ET
BAG74777|Hordeum_vulgare_subsp._vulgare__domestic YLDGGYHVPYAHGALAS.GLQLQ.......SYETLT..YEKVSVQRC......ESAPA...EQ
AKZ66518|Triticum_monococcum_subsp._aegilopoides ..............................................................
FAOM01578495|Triticum_aestivum_UpdateAMA02065  YLDGGYHVPYAHGALAS.GLQLQ.......SYETLT..YERVSVQRC......ESAPA...EQ
EMT07994|Aegilops_tauschii_                    YLDGGYHVPYAHGALAS.GLQLQ.......SYETLT..YEKVSVQRC......ESAPE...EQ
ACZ92178|Triticum_turgidum_subsp._durum__durum_wh ..............................T..YERVSVQRC......ESAPA...EQ
KD266899|Triticum_urartu_UpdateEMS46986        YLDGGYHVPYAHGALAS.GLQLQ.......SYETLT..YERVSVQRC......ESALA...EQ
ABV64740|Leymus_chinensis                      YLDGGYHVPYAHGALAS.GLQLQ.......SYETLT..YERVSVQRC......ESAPA...EQ
Brast07G193900.1|Brachypodium_stacei           YLDGGYHVPYAHGALAS.GLQLQ.......SYKTLT..YERVSVQRC......ESAPA...EQ
XP_003563491|Brachypodium_distachyon__stiff_brome YLDGGYHVPYAHGALAS.GLQLQ.......SYETLT..YERVSVQRC......ESAPA...EQ
XP_006657294|Oryza_brachyantha__malo_sina_     YLDGGYHVPYAHGALAS.GLQLQ.......SYETLT..YERVSVQRC......ESGQV...EQ
XP_015643170|Oryza_sativa_Japonica_Group__Japanes YLDGGYHVPYAHGTLAS.GLQLQ.......SYETHT..YERVSVQRC......ESVQA...EQ
EEC81252|Oryza_sativa_Indica_Group__long-grained_ YLDGGYHVPYAHGTLAS.GLQLQ.......SYETHT..YERVSVQRC......ESVQA...EQ
XP_002437525|Sorghum_bicolor__sorghum_         YLDGGYHVPYAHGALAS.GLQLQ.......SYETLT..YERVSVQRC......ESAPA...EP
NP_001105926|Zea_mays                          YLDGGYHVPYAHGDLAS.GLQLQ.......SYETLT..YERVSVQRC......ESAPA...EP
Pavir.Db00260.1|Panicum_virgatum_CORR_316f     YLDGGYHIPYAHGALAS.GLQLQ.......SYETLT..YERVSVQRC......ESAPS...ES
LWDX02005496.1|Dichanthelium_oligosanthes_update_ YLDGGYHVPYAHGALAS.GLQLQ.......SYETLQT.YERVSVQRC......ESAPS...ES
Sevir.4G257300.1|Setaria_viridis               YLDGGYHVPYAHGALAS.GLQLQ.......SYETLQA.YERVSVQRC......ESAPS...EP
XP_004966128|Setaria_italica__foxtail_millet_  YLDGGYHVPYAHGALAS.GLQLQ.......SYETLA..YERVSVQRC......ESAPS...EP
Oropetium_20150105_16264A|Oropetium_thomaeum   YLDGGYHVPYAHGALAS.GLQLQ.......SYETL...........................
Pahal.D00004.1|Panicum_hallii                  YLDGGYHVPYAHGALAS.GLQLQ.......SYETLT..YERVSVQRC......ESAPS...ES
Pavir.J39646.1|Panicum_virgatum_Corr_363f      YLDGGYHVPYAHGALAS.GLQLQ.......SYETLT..YERVSVQRC......ESAPS...ES
OAY65202|Ananas_comosus__pineapple_            YLDGGYHVPYAHKGLAS.GLELN.......SYETLM..FEKVSVQRC......ESASV...EQ
XP_009410214|Musa_acuminata_subsp._malaccensis__w YLDGGYHVPYAHGDLAS.GLNLD.......SYSTLI..FEKVSIQRC......EGGPT...DK
XP_010927268|Elaeis_guineensis__African_oil_palm_ YLDGGYHVPYAHGGLAS.GLKLD.......SYSTLI..FEKVSIQRC......EGASV....
XP_008801514|Phoenix_dactylifera__date_palm_   YLDGGYHVPYAHGGLAS.GLKLD.......SYSTLI..FEKVSIQRC......EGASV...DE
KMZ72255|Zostera_marina                        YLDGGYHVPYAHRDLAS.GLTMD.......SYSTTI..FEKVSIQRC......ESAVI..ENG
JAT51600|Anthurium_amnicola                    YLDGGYHVPYAHGGLAC.GLKLN.......SYLTDV..FEKVSIQRC......ESSSI...RD
Spipo5G0003400|Spirodela_polyrhiza             YLDGGYHVPYAHGGLAS.GLDLD.......SYTTSV..FERVSIQEC......RSAAA....A
XP_006845775|Amborella_trichopoda              YLDGGYHVPYAHAALAS.GLNLQ.......TYSIDI..FEKVSIQTC......EGIQE..GSK
AFP19450|Camellia_sinensis                     YLDGGYHVPYAHKGLSS.GLNLE.......SYSTTT..FEKVSIQQC......DGGS..AET
XP_017254907|Daucus_carota_subsp._sativus      YLDGGYHVPYAHKGLAS.GLNLE.......SYSTSV..FEKVSIQRC......YSGQV...DS
ABX57826|Chrysanthemum_lavandulifolium         YLDGGYHVPFAHKDLAS.GLKLD.......SYSTTV..FEKVSIQRC......DGGEV...QQ
LEKV01000036.1|Cynara_cardunculus_update_KVI11819 YLDGGYHVPFAHKDLAS.GLKLD.......SYSTTI..YEKVSIQRC..............DG
XP_008443631|Cucumis_melo__muskmelon_          YLDGGYHVPYAHKGLAS.NLNLE.......SYSTEL..FETVSIQSC......KGGGE...SK
XP_004139149|Cucumis_sativus__cucumber_        YLDGGYHVPYAHKGLAS.NLKLE.......SYSTEL..FETVSIQSC......KGGGE...SK
XP_008351406|Malus_domestica__apple_           YLDGGYHVPYAHKGLAS.GLNLD.......GYSTTM..YEKVSIQKC......ENGST...ER
XP_008391562|Malus_domestica__apple_           YLDGGYHVPYAHKGLAS.GLNLD.......GYSTTM..YEKVSIQKC......EXGSQ...KE
XP_009343851|Pyrus_x_bretschneideri__Chinese_whit YLDGGYHVPYAHKGLAS.GLNLD.......GYSTTM..YEKVSIQKC......ESGST...ER
AER10510|Pyrus_betulifolia_corr_405aa          YLDGGYHVPYAHKGLAS.GLNLD.......GYSTTM..YEKVSIQKC......ESGST...ER
XP_008223853|Prunus_mume__Japanese_apricot_    YLDGGYHVPYAHKGLAS.GLNLD.......HYSTTI..YEKVSIQKC......EGGST...ER
ONI27175|Prunus_persica__peach_                YLDGGYHVPYAHKGLAS.GLNLD.......HYSTTI..YEKVSIQKC......EGGST...ER
```

**XP_004301419|Fragaria_vesca_subsp._vesca**	YLDGGYHVPYAHKDLAS.GLNLN.......GYSTTV..YEKVSIQMC.....EGGST...EK
**XP_015889685|Ziziphus_jujuba__common_jujube_**	YLDGGYHVPYAHKGLAS.GLNLD.......SYSTTI..FERVSIQKC.....EGGST.....
**NW_010360102.1|Morus_notabilis_corrected**	YLDGGYHVPYAHKGLAN.GLKLD.......SYSTTI..FEKVSIQIC.....EGGSM...EG
**Lus10032689|Linum_usitatissimum**	YLDGGYHVPYAHKGLAS.GLELD.......SYSTTT..YEKVSIQSC.....EGAS......
**Lus10008571|Linum_usitatissimum_corrected**	YLDGGYHVPYAHKGLAS.GLELD.......SYSTTT..YGKVSIQSC.....KGGS......
**XP_011020077|Populus_euphratica__Euphrates_poplar**	YLDGGYHVPYAHKGLAS.GLKLN.......SYSTKT..YEKVSIQSC.....DGGST...ES
**XP_002308100|Populus_trichocarpa__Populus_balsami**	YLDGGYHVPYAHKGLAS.GLKLN.......SYSTKT..YEKVSIQSC.....DGGST...ES
**SapurV1A.0198s0060.1|Salix_purpurea**	YLDGGYHVPYAHKGLAS.GLNLN.......SYSTTT..YEKVSIQSC.....DGGST...ES
**OAY46667|Manihot_esculenta__cassava_**	YLDGGYHVPHAHKALAS.GLKLD.......SYSTIM..YEKVSIQRC.....EGGST...GS
**XP_002518256|Ricinus_communis__castor_bean_**	YLDGGYHVPFAHKSLAS.GLKLD.......SYSTTI..FERASIQKC.....EGGSM...GS
**XP_012074438|Jatropha_curcas**	YLDGGYHVPYAHKALAS.GLKLD.......SYSTSV..YEKVSIQRC.....EGSST...QS
**XP_010046909|Eucalyptus_grandis**	YLDGGYHVPYAHKGLAS.GLKLD.......SYSTTI..HEKVSLQIC.....EGGST...NN
**ABS71853|Eucalyptus_camaldulensis_Murray_red_gum_**	YLDGGYHVPYAHKGLAS.GLKLD.......SYSTTI..HEKVSLQIC.....EGGST...NN
**XP_006474292|Citrus_sinensis__sweet_orange_**	YLDGGYHVPYAHKGLAS.GLQLD.......SYSTTL..YEKVSVQRC.....ESGST...EG
**XP_006453220|Citrus_clementina_reviewed**	YLDGGYHVPYAHKGLAS.GLQLDLTQQRVESNYAVL..YEKVSVQRC.....ESGST...EG
**XP_017612508|Gossypium_arboreum**	YLDGGYHVPFAHKGLAS.GLSLD.......SYTTSI..FEKVSIQSA.....EGGSK....Q
**XP_016686114|Gossypium_hirsutum__cotton_**	YLDGGYHVPFAHKGLAS.GLSLD.......SYTTSI..FEKVSIQSA.....EGGSK....Q
**XP_012459381|Gossypium_raimondii**	YLDGGYHVPFAHKGLAS.GLSLD.......SYTTSI..FEKVSIQSA.....EGGSK....E
**XP_016719040|Gossypium_hirsutum__cotton_**	YLDGGYHVPFAHKGLAS.GLSLD.......SYTTSI..FEKVSIQSA.....EGGSK....E
**EOY05290|Theobroma_cacao__cacao_**	YLDGGYHVPYAHKGLAS.GLTLD.......SYTTSI..FEKVSIQSV.....EGGSK....E
**AWWV01015497-AWWV01004954_Corchorus_capsularis_39**	YLDGGYHVPYAHKGLAS.GLSLD.......SYTTTI..FEKVSIQSV.....EGGS....QE
**OMO51681|Corchorus_olitorius**	YLDGGYHVPYAHKGLAS.GLSLD.......SYTTTI..FEKVSIQSV.....EGGS....QE
**GAV59711|Cephalotus_follicularis**	YLDGGYHVPYAHKGLAS.GLKLD.......SYSTSV..FEKVSIQRC.....EGGSM...QT
**XP_018807260|Juglans_regia__English_walnut_**	YLDGGYHVPYAHKGLAF.GLQLQ.......SYSTTI..FENVSIQSC.....EGGTM...ER
**XP_019078540|Vitis_vinifera__wine_grape_**	YLDGGYHVPYAHKGLAS.GLKLE.......SYSTTT..FERVSIQSC.....EGGPG...ES
**Aqcoe7G261800.1|Aquilegia_coerulea**	YLDGGYHVPYAHTGLAS.GLKLE.......SYSTTM..FEKVSVQIC.....ESGSR...EN
**XP_010276721|Nelumbo_nucifera__sacred_lotus_**	YLDGGYHVPYAHKDLAS.GLKLE.......SYSTTI..LEKASIQQC.....ESCV....EG
**XP_016201893|Arachis_ipaensis**	YLDGGYHVPYAHKGLAS.GLKFD.......SYSTTI..FERVSIQSC.....EGSSG...ES
**XP_015973237|Arachis_duranensis**	YLDGGYHVPYAHKGLAS.GLKFD.......SYSTTI..FERVSIQSC.....EGSSG...ES
**Tp57577_TGAC_v2_mRNA5577|Trifolium_pratense**	YLDGGYHVPYAHKGLAS.GLNLD.......SYSTTL..FERVSIQSC.....EGSSG...KS
**GAU38011|Trifolium_subterraneum**	YLDGGYHVPYAHKGLAS.GLNLD.......SYSTTL..FERVSIQSC.....EGSSR...KS
**XP_003610028|Medicago_truncatula__barrel_medic_**	YLDGGYHVPYAHKDLAS.GLNLD.......SYSTKL..FERVSIQSC.....EGGSE...KS
**XP_004507911|Cicer_arietinum__chickpea_**	YLDGGYHVPYAHKGLAS.GLNLD.......SYSITL..FEKVSIQSC.....EGSSS..EKS
**XP_019463425|Lupinus_angustifolius__narrow-leaved**	YLDGGYHVPYAHKGLAS.GLKLD.......SYSISM..FERVSIQSC.....ESNSG...KS
**KYP52554|Cajanus_cajan__pigeon_pea_**	YLDGGYHVPYAHKGLAS.GLKMD.......SYAITM..FERVSIQSC.....EGSSE...KS
**XP_003549280|Glycine_max__soybean_**	YLDGGYHVPYAHKGLAS.GLKLD.......SYSITM..FERVSIQSC.....EGSSE...KN
**XP_007134857|Phaseolus_vulgaris_CHR1_corr_208aaf**	NLDGGYHVPYAHKGLVS.DLKMD.......SYSITM..FEKVSIQSC.....EGSSE...KS
**XP_007154695|Phaseolus_vulgaris_CORR**	YLDGGYHVPYAHKGLVS.GLKMD.......SYSITM..FEKVSIQSC.....EGSSE...KN
**XP_017410379|Vigna_angularis__adzuki_bean_**	YLDGGYHVPYAHKGLAS.GLKMD.......SYSITM..FEKVSIQSC.....EGSSE...KS
**XP_014508581|Vigna_radiata_var._radiata__mung_bea**	YLDGGYHVPYAHKGLAS.GLKMD.......SYSITM..FEKVSIQSC.....EGSSE...KS
**AUSU01000986.1|Genlisea_aurea_corrected-f**	YLDGGYHVPYAHKGLSS.SLQLD.......SYSTEV..YERVSIQSC.............TG
**OAE24413|CORR_Marchantia_polymorpha**	YLDGGYHIPHAHASLAA.SLNLK.......QYNTTL..SEKVSIQTC.....MVAAS...GG
**Sphfalx0064s0059.1|Sphagnum**	YLDGGYHVPHAHSALAS.SLDLS.......SYSTTV..FEKVSIQSC.......HPV.....
**XP_001752587|Physcomitrella_patens**	YLDGGYHVPHAHSSLAA.CLDLP.......SYSTSL..LERVSIQSC.......GAA.....
**XP_002508933|Micromonas_sp._RCC299**	YLDGGYHVPFAHPALVTDGVDMR.......KYETEV.YGEYVSVQTH......GAPA.....
**XP_003061830|Micromonas_pusilla_CCMP1545**	YLDGGYHVPFAHPELAS.GVSMK.......SYETTI..RGLHSLQTV.....TAAAAA..AT
**XP_005645368|Coccomyxa**	YLDGGYHVSIAHPDLAA.GLDLT.......TYSSTI..YESCSIQSC........QP.....
**ADIC01002027.1|Chlorella_variabilis_corrected_pre**	YLDGGYHVSVAHPELAS.GLDLA.......TYRSTL..CERLSIQSC.....QPAAAA.AAA
**XP_013903967|Monoraphidium_neglectum_rev**	YLDGGYHVPIAHPGLAA.QLDLG.......GYRSEL..HPRLSLQTC.......DPG.....
**XP_005644270|Coccomyxa_subellipsoidea_C-169**	YLDTCYHCPFAHPGLCE.ALDMD.......EYHSVC..YEHNSFQFS.....PLAPP.....
**NC_024001.1|Bathycoccus_prasinos_corrected**	YLDGGFHVPFAHKALVKEGCDMS.......KYNITL.FDNMNSIQSV........DV..RKE
**EWM23896|Nannochloropsis_gaditana**	YLDNGYHVPYAHKALGN.ALDLS.......SYGASV..HDRYSVQAC.....RGAPST.SSP
**XP_002738379|Saccoglossus_kowalevskii**	YLDGGYHVSTAHKDLSV.ALDAS.......SYRTAV..HEWHSIQSV........SA.....
**XP_006820984|Saccoglossus_kowalevskii**	VLDNGYHVTMTHPNYSK.LLELN.......SLKTKL..LSRSSICTV........ES.....
**XP_002737044|Saccoglossus_kowalevskii**	FIDGGYHIDYAHKIFTGSYYDFD.......TYRMLE.VNKDYSTQIV........DT.....
**XP_002597329|Branchiostoma_floridae__Florida_lanc**	AQDHNSHVEYVHKDLAK.IVDLP.......STKVQA..TELYSKMTG......QGAT...EN
**XP_019618440|Branchiostoma_belcheri__Belcher's_la**	AQDHNSHVEYVHKDLAK.IVDLP.......STKVEA..TDLYSKMTG.........LG....
**XP_019644608|Branchiostoma_belcheri__Belcher's_la**	AADSGYHLEYVHRDLSS.NLDLS.......TFRTEV..GDIYNMQVC.........IG...K
**XP_019644788|Branchiostoma_belcheri__Belcher's_la**	AADSGYHLEYVHRDLSS.NLDLS.......TFRTEV..GDIYNMQVC.........IG...K
**XP_002610312|Branchiostoma_floridae__Florida_lanc**	ASDGGYHLEYVHRDLSS.NLDLS.......TFRTEV..GDIYNMQVC.........IG...K
**XP_002599795|Branchiostoma_floridae__Florida_lanc**	YLDGGYHVEYLHRNLAS.NLSID.......TYRTEV..QDLFSVQTC...............
**XP_019633636|Branchiostoma_belcheri__Belcher's_la**	YLDGGYHVEYLHRNLAS.NLSID.......TYRTDV..QELYSVQTC......SGSA.....
**XP_004333794|Acanthamoeba_castellanii_str._Neff**	YLDGGYHVSVLHKTLNA.QLDAS.......QYSTQV..HGRWSLQSC...............
**GAQ84770|Klebsormidium_flaccidum**	YLDGGYHIPYNHKELAA.SVDID.......TYTTTC..FSKGTFQTV........DV.....

```
                                                                                   330       340
BAC77698|Atriplex_nummularia                          NNG........................FN...RL......GT.Q.AFYAFAYPNFAVERYGP
AGC13074|Atriplex_semibaccata                         NNG........................FN...RL......GT.Q.AFYAFAYPNFAVERYGP
AHH24260|Atriplex_amnicola                            NNG........................FN...RL......GT.Q.AFYAFAYPNFAVERYGP
AFG28558|Atriplex_canescens__four-winged_saltbush     NNG........................FN...RL......GS.Q.AFYAFAYPNFAVERYGP
Q9LKN0|Atriplex_hortensis                             NNG........................FN...RL......GT.Q.AFYAFAYPNFAVERYGP
AAL92561|Atriplex_prostrata                           NNG........................FS...RI......GT.Q.AFYAFAYPNFAVERYGP
O04121|Spinacia_oleracea__spinach_                    PDG........................FD...RV......GI.Q.AFYAFAYPNFAVERYGP
ABG34274|Ophiopogon_japonicus                         PDG........................FD...RV......GI.Q.AFYAFAYPNFAVERYGP
AEW31326|Haloxylon_persicum                           PDG........................FD...RL......GS.Q.AFYAFAYPNFAVERYGP
ACX47904|Haloxylon_ammodendron                        PDG........................FD...RL......GS.Q.AFYAFAYPNFAVERYGP
AJF98574|Salicornia_bigelovii                         PDG........................FD...RL......GN.Q.AFYAFAYPNVAVERYGP
AAV91779|Salicornia_europaea                          PDG........................FD...RL......GN.Q.AFYAFAYPNFAVERYGP
AFW04225|Suaeda_maritima                              PDG........................FD...RL......GE.K.AFYAFTYPNFAVERYGP
AAM43920|Suaeda_liaotungensis                         PDG........................FD...RL......GE.K.AFYAFTYPNFAVERYGP
XP_010682183|Beta_vulgaris_subsp._vulgaris__sugar     PDG........................FD...RL......GT.E.AFYAFIYPNFAVERYGT
AHYPO_002206-RA|Amaranthus_hypochondriacus_439        PNG........................FN...RL......GS.E.AFYAFIYPNFAVERYGP
BAF93187|Amaranthus_tricolor                          PNG........................FD...RL......GS.K.AFYAFIYPNFAVERYGP
XP_012828802|Erythranthe_guttata__spotted_monkey_     DSE......................EEFD...RL......GS.K.AFYAFVYPNFMINRYGP
Migut.J00779.1|Mimulus_guttatus                       DSE......................EEFD...RL......GS.K.AFYAFVYPNFMINRYGP
EYU18199|Erythranthe_guttata__spotted_monkey_flow     GRE......................EEFD...RL......GS.K.AFYAFVYPNFMISRYGP
KQ992415.1|Dorcoceras_hygrometricum_corrected         NEE........................FV...RL......GS.K.ALYAFVYPNFMINRYGP
XP_011094129|Sesamum_indicum__sesame_                 FEQ.......................EFD...RL......GS.K.ALYAFVYPNFMINRYGP
XP_015080536|Solanum_pennellii__Lycopersicon_penn     KDQ.......................DFD...RL......GS.K.ALYAFVYPNFMINRYGP
XP_004242785|Solanum_lycopersicum__Lycopersicon_e     KDQ.......................DFH...RL......GS.K.ALYAFVYPNFMINRYGP
NP_001275051|Solanum_tuberosum__potato_               KDQ.......................NFD...RL......GS.K.ALYAFVYPNFMINRYGP
XP_016556147|Capsicum_annuum                          KGQ.......................EFD...RL......GS.K.ALYAFVYPNFMINRYGP
ACR15118|Lycium_barbarum                              DDQ.......................EFD...RL......GS.K.ALYAFVYPNFMINRYGP
XP_009757252|Nicotiana_sylvestris__wood_tobacco_      KDQ.......................EFD...RL......GS.K.ALYAFVYPNFMINRYGP
XP_016460838|Nicotiana_tabacum_(common_tobacco)       KDQ.......................EFD...RL......GS.K.ALYAFVYPNFMINRYGP
OIT26339|Nicotiana_attenuata                          KGQ.......................EFD...RL......GS.E.ALYAFVYPNFMINRYGP
XP_009601434|Nicotiana_tomentosiformis                KDQ.......................EFD...RL......GS.K.ALYAFVYPNFMINRYGP
XP_016508540|Nicotiana_tabacum_(common_tobacco)       KDQ.......................EFD...RL......GS.K.ALYAFVYPNFMINRYGP
XP_015081828|Solanum_pennellii__Lycopersicon_penn     NDL.......................DFD...RI......GS.KPALYAFVYPNFIISRYGP
XP_004243034|Solanum_lycopersicum__Lycopersicon_e     NDL.......................DFD...RI......GS.KPALYAFVYPNFIISRYGP
CDP08949|Coffea_canephora                             SAP.......................DFD...RL......GS.K.ALYAFIYPNFMVNRYGP
XP_019182381|Ipomoea_nil__Japanese_morning_glory_     DE.........................FD...RL......GS.K.ATYAFVFPNFMINRYGP
XP_010433050|Camelina_sativa__false_flax_             EDG........................FD...RL......GS.Q.ALYAFVYPNFMINRYGP
XP_010436340|Camelina_sativa_false_flax_corr          EDG........................FD...RL......GS.Q.ALYAFVYPNFMINRYGP
XP_010438247|Camelina_sativa__false_flax_             EDG........................FD...RL......GS.Q.ALYAFVYPNFMINRYGP
XP_010447794|Camelina_sativa__false_flax_             EDG........................FD...RL......GS.Q.ALYAFVYPNFMINRYGP
XP_006285614|Capsella_rubella                         EDG........................FN...RL......GS.Q.ALYAFVYPNFMINRYGP
Cagra.0268s0015.1|Capsella_grandiflora                EDG........................FD...RL......GS.Q.ALYAFVYPNFMINRYGP
Bostr.7867s0836.1|Boechera_stricta                    EDG........................FD...RL......GS.Q.ALYAFVYPNFMINRYGP
NP_194718|Arabidopsis_thaliana__thale_cress_          EDG........................FD...RL......GS.E.ALYAFVYPNFMINRYGP
XP_002869406|Arabidopsis_lyrata_subsp._lyrata         EDG........................FD...RL......GS.Q.ALYAFVYPNFMINRYGP
Araha.6052s0002.1|Arabidopsis_halleri                 EDG........................FN...RL......GC.Q.ALYAFVYPNFMINRYGP
XP_006412796|Eutrema_salsugineum                      KDG........................FD...RL......GS.Q.ALYAFVYPNFMINRYGP
KFK29548|Arabis_alpina__gray_rockcress_               EGG........................FD...RL......GS.R.ALYAFVYPNFMINRYGP
KFK40841|Arabis_alpina__gray_rockcress_               ..............................................................
XP_018481725|Raphanus_sativus__radish_                EDG........................FD...RL......GS.Q.ALYAFVYPNFMINRYGP
XP_018435688|Raphanus_sativus__radish_                EDG........................FD...RL......GS.Q.ALYAFVYPNFMINRYGP
XP_013705395|Brassica_napus__rape_                    EDG.......................GFD...RL......GS.Q.ALYAFVYPNFMINRYGP
XP_013596413|Brassica_oleracea_var._oleracea          EDG.......................GFD...RL......GS.Q.ALYAFVYPNFMINRYGP
XP_009137872|Brassica_rapa__field_mustard_            EDG........................FD...RL......GS.Q.ALYAFVYPNFMINRYGP
XP_013738248|Brassica_napus__rape_                    EDG........................FD...RL......GS.Q.ALYAFVYPNFMINRYGP
XP_010541096|Tarenaya_hassleriana                     EDG........................YD...RL......GS.K.ALYAFIYPNFMINRYGP
Kaladp0809s0111.1|Kalanchoe_fedtschenkoi              SDE........................FD...RL......GS.E.ALYAYIYPNFMINRYGP
Kalax.0333s0026.1|Kalanchoe_laxiflora_corr_462aa      SDE........................FD...RL......GS.E.ALYAYIYPNFMINRYGP
Kalax.0012s0114.1|Kalanchoe_laxiflora_corr_462aa      SDE........................FD...RL......GS.E.TLYAYIYPNFMINRYGP
XP_010667950|Beta_vulgaris_subsp._vulgaris__sugar     GDE........................FD...RL......GS.K.ALYAFIYPNFMINRYGP
AH021158-RA|Amaranthus_hypochondriacus_421aa-corr     GN.........................ND...RL......GS.T.ALYAFIYPNFMINRYGP
XP_021866412|426|Spinacia_oleracea_(spinach)          GED........................FD...RL......GS.K.ALYAFVYPNFMINRYGP
BAG74777|Hordeum_vulgare_subsp._vulgare__domestic     ED.........................ID...RL......GT.K.ATYAFVYPNFMINRYGP
AKZ66518|Triticum_monococcum_subsp._aegilopoides      ..............................................................
FAOM01578495|Triticum_aestivum_UpdateAMA02065         ED.........................ID...RL......GT.K.ATYAFVYPNFMINRYGP
EMT07994|Aegilops_tauschii_                           D..........................ID...RL......GT.K.ATYAFVYPNFMINRYGP
ACZ92178|Triticum_turgidum_subsp._durum__durum_wh     ED.........................ID...PL......RD.K.ATYAFVYPNFMINRYGP
KD266899|Triticum_urartu_UpdateEMS46986               ED.........................ID...RL......GT.K.ATYAFVYPNFMINRYGP
ABV64740|Leymus_chinensis                             ED.........................ID...RL......GT.K.ATYAFVYPNFMINRYGP
Brast07G193900.1|Brachypodium_stacei                  ED.........................ID...RL......GT.K.AIYAFVYPNFMINRYGP
XP_003563491|Brachypodium_distachyon__stiff_brome     ED.........................ID...RL......GT.K.AIYAFVYPNFMINRYGP
XP_006657294|Oryza_brachyantha__malo_sina_            ND.........................FD...RL......GT.K.ATYAFVYPNFMINRYGP
XP_015643170|Oryza_sativa_Japonica_Group__Japanes     ND.........................FD...RL......GT.K.AIYAFVYPNFMINRYGP
EEC81252|Oryza_sativa_Indica_Group__long-grained_     ND.........................FD...RL......GT.K.AIYAFVYPNFMINRYGP
XP_002437525|Sorghum_bicolor__sorghum_                DD.........................FD...RL......GT.K.ALYAFVYPNFMINRYGP
NP_001105926|Zea_mays                                 DD.........................FD...RL......GT.K.ALYAFVYPNFMINRYGP
Pavir.Db00260.1|Panicum_virgatum_CORR_316f            DD.........................LE...RL......GT.K.ALYAFVYPNFMINR...
LWDX02005496.1|Dichanthelium_oligosanthes_update_     DD.........................FE...RL......GT.K.ALYAFVYPNFMINRYGP
Sevir.4G257300.1|Setaria_viridis                      DD.........................FE...RL......GT.K.ALYAFVYPNFMINRYGP
XP_004966128|Setaria_italica__foxtail_millet_         DD.........................FE...RL......GT.K.ALYAFVYPNFMINRYGP
Oropetium_20150105_16264A|Oropetium_thomaeum          ..............................................................
Pahal.D00004.1|Panicum_hallii                         DD.........................LE...RL......GT.K.ALYAFVYPNFMINRYGP
Pavir.J39646.1|Panicum_virgatum_Corr_363f             DD.........................LE...RL......GT.K.ALYAFVYPNFMINRYGP
OAY65202|Ananas_comosus__pineapple_                   DS.........................FD...RL......GS.K.AIYAFVYPNFMINRYGP
XP_009410214|Musa_acuminata_subsp._malaccensis__w     DG.........................FD...RL......GS.K.ALYAFVYPNFMINRYGP
XP_010927268|Elaeis_guineensis__African_oil_palm_     DEVG.......................FD...RL......GS.K.AVYAFIYPNFMVNRYGP
XP_008801514|Phoenix_dactylifera__date_palm_          DD.........................FD...RL......GS.K.AVYAFIYPNFMINRYGP
KMZ72255|Zostera_marina                               DD.........................FD...RL......GA.K.AFYAFVYPNFMINRYGH
JAT51600|Anthurium_amnicola                           DD.........................FD...RL......GS.K.ALYAFVYPNFMINRYGP
Spipo5G0003400|Spirodela_polyrhiza                    GNG........................PG...RL......GS.R.ALYAFVYPNFMINRYGP
XP_006845775|Amborella_trichopoda                     KD.........................FD...RL......GS.K.AMYAFIYPNFMINRYGP
AFP19450|Camellia_sinensis                            ENE........................YE...RL......GS.K.ALYAFIYPNFMINRYGP
XP_017254907|Daucus_carota_subsp._sativus             HED........................TD...RL......GS.K.ALYAFIYPNFMINRYGP
ABX57826|Chrysanthemum_lavandulifolium                QED........................FD...RL......GA.K.SLYAFIYPNFMVNRYGP
LEKV01000036.1|Cynara_cardunculus_update_KVI11819     DED........................FD...RL......GS.K.SLYAFIYPNFMVNRYGP
XP_008443631|Cucumis_melo__muskmelon_                 GDD........................YG...RL......GP.E.ALYAFIYPNFMINRYGP
XP_004139149|Cucumis_sativus__cucumber_               GDD........................IG...RL......GS.E.ALYAFVYPNFMINRYGP
XP_008351406|Malus_domestica__apple_                  KND........................FD...RL......GS.K.ALYAFVYPNFMINRYGP
XP_008391562|Malus_domestica__apple_                  KND........................FD...RL......GS.K.ALYAFVYPNFMINRYGP
XP_009343851|Pyrus_x_bretschneideri__Chinese_whit     KND........................FD...RL......GS.K.ALYAFVYPNFMINRYGP
AER10510|Pyrus_betulifolia_corr_405aa                 KND........................FD...RL......GS.K.ALYAFVYPNFMINRYGP
XP_008223853|Prunus_mume__Japanese_apricot_           TND........................YD...RL......GS.K.ALYAFVYPNFMINRYGP
ONI27175|Prunus_persica__peach_                       TND........................YD...RL......GS.K.ALYAFVYPNFMINRYGP
```

**XP_004301419|Fragaria_vesca_subsp._vesca**	KDD........................HD...RL......GS.K.ALYAFIYPNFMINRYGP
**XP_015889685|Ziziphus_jujuba__common_jujube_**	END........................YD...RL......GS.K.ALYAFVFPNFMINRYGP
**NW_010360102.1|Morus_notabilis_corrected**	KDD........................YD...RL......GS.K.AFYAFIYPNFMINRYGP
**Lus10032689|Linum_usitatissimum**	...........................TE...RL......GS.K.ALYAFIYPNFMINRYGP
**Lus10008571|Linum_usitatissimum_corrected**	...........................TE...RL......GS.K.ALYAFIYPNFMINRYGP
**XP_011020077|Populus_euphratica__Euphrates_poplar**	EDD........................ID...RL......GS.K.ALYAFIYPNFMINRYGP
**XP_002308100|Populus_trichocarpa__Populus_balsami**	EDD........................ID...RL......GS.K.ALYAFIYPNFMINRYGP
**SapurV1A.0198s0060.1|Salix_purpurea**	EDD........................ID...RL......GS.K.ALYAFIYPNFMINRYGP
**OAY46667|Manihot_esculenta__cassava_**	EED........................FG...RL......GS.K.ALYAFIYPNFMINRYGP
**XP_002518256|Ricinus_communis__castor_bean_**	VDE........................FD...RL......GS.K.AIYAFIFPNFMINRYGP
**XP_012074438|Jatropha_curcas**	ADD........................FG...RL......GS.K.ALYAFIYPNFMINRYGP
**XP_010046909|Eucalyptus_grandis**	AND........................FD...RL......GA.E.ALYAFIYPNFMINRYGP
**ABS71853|Eucalyptus_camaldulensis_Murray_red_gum_**	AND........................FD...RL......GA.E.ALYAFIYPNFMINRYGP
**XP_006474292|Citrus_sinensis__sweet_orange_**	TDD........................TH...RL......GS.K.AFYAFIYPNFMINRYGP
**XP_006453220|Citrus_clementina_reviewed**	TDD........................TH...RL......GS.K.AFYAFIYPNFMINRYGP
**XP_017612508|Gossypium_arboreum**	KE.........................DD...RL......GS.K.AFYAFIYPNFMINRYGP
**XP_016686114|Gossypium_hirsutum__cotton_**	KE.........................DD...RL......GS.K.AFYAFIYPNFMINRYGP
**XP_012459381|Gossypium_raimondii**	KE.........................DD...RL......GS.K.AFYAFIYPNFMINRYGP
**XP_016719040|Gossypium_hirsutum__cotton_**	KE.........................DD...RL......GS.K.AFYAFIYPNFMINRYGP
**EOY05290|Theobroma_cacao__cacao_**	TK.........................DD...RL......GS.N.ALYAFIYPNFMINRYGP
**AWWV01015497-AWWV01004954_Corchorus_capsularis_39**	T..........................ED..YRL......GS.K.AFYAFVYPNFMINRYGP
**OMO51681|Corchorus_olitorius**	T..........................ED..YRL......GS.K.AFYAFVYPNFMINRYGP
**GAV59711|Cephalotus_follicularis**	EDD........................ID...RL......GS.K.ALYTFIYPNFMINRYGP
**XP_018807260|Juglans_regia__English_walnut_**	KDE........................YD...RL......GS.K.ALYAFVYPNFMINRYGP
**XP_019078540|Vitis_vinifera__wine_grape_**	EDD........................FD...RL......GT.K.ALYAFIYPNFMINRYGP
**Aqcoe7G261800.1|Aquilegia_coerulea**	GGD........................YD...RL......GS.K.AIYAFIYPNFMINRYGP
**XP_010276721|Nelumbo_nucifera__sacred_lotus_**	GDD........................FD...RL......GS.K.ALYAFIYPNFMINRYGP
**XP_016201893|Arachis_ipaensis**	KDN........................YD...RL......GR.K.AIYAFIYPNFMINRYGP
**XP_015973237|Arachis_duranensis**	KDN........................YD...RL......GR.K.AIYAFIYPNFMINRYGP
**Tp57577_TGAC_v2_mRNA5577|Trifolium_pratense**	AEN........................ND...RL......GR.K.AIYAFIYPNFMINRYGP
**GAU38011|Trifolium_subterraneum**	TEN........................ND...RL......GR.K.AIYAFIYPNFMINRYGP
**XP_003610028|Medicago_truncatula__barrel_medic_**	KEN........................ND...RL......GR.K.AIYAFIYPNFMINRYGP
**XP_004507911|Cicer_arietinum__chickpea_**	KEN........................YD...RL......GR.K.AIYAFVYPNFMINRYGP
**XP_019463425|Lupinus_angustifolius__narrow-leaved**	KEN........................YD...RL......GR.K.PIYAFIYPNFMINRYGP
**KYP52554|Cajanus_cajan__pigeon_pea_**	KEN........................YD...RV......GK.K.AIYAFIYPNFMINRYGP
**XP_003549280|Glycine_max__soybean_**	KGN........................YD...RL......GR.K.AIYAFVYPNFMINRYGP
**XP_007134857|Phaseolus_vulgaris_CHR1_corr_208aaf**	EEN........................YD...RL......GR.K.AIYAFIYPNFMINRYGP
**XP_007154695|Phaseolus_vulgaris_CORR**	EEN........................YD...RL......GR.K.AIYAFIYPNFMINRYGP
**XP_017410379|Vigna_angularis__adzuki_bean_**	EEN........................YD...RL......GR.K.AIYAFIYPNFMINRYGP
**XP_014508581|Vigna_radiata_var._radiata__mung_bea**	EEN........................YD...RL......GR.K.AIYAFIYPNFMINRYGP
**AUSU01000986.1|Genlisea_aurea_corrected-f**	KKQ........................AS...RL......GS.R.AIYAFIYPNFMINRYGP
**OAE24413|CORR_Marchantia_polymorpha**	DHRY.........VSNGETQISSTNDRKK...RR......AD.N.AVFAFVYPNFMINRYGP
**Sphfalx0064s0059.1|Sphagnum**	RAT.....................GADSD..TRVA.....GP...ATYAFVYPNFMINRYGS
**XP_001752587|Physcomitrella_patens**	KES.............................GLV.....RVGNVATYAFVYPNFMINRYGP
**XP_002508933|Micromonas_sp._RCC299**	AEL.........................T...RL......GD.S.ALYAFVYPNLMINRYGP
**XP_003061830|Micromonas_pusilla_CCMP1545**	DRV.....................RDRDD...RL......GD.A.ALYAFIHPNFMVNRYGK
**XP_005645368|Coccomyxa**	KNA.........................A...KVR....LGERE.AAYAFVYPNLMINRYGP
**ADIC01002027.1|Chlorella_variabilis_corrected_pre**	EAG...............EAHAGTSGGSAGEQGMWRRQRIAGGRPPAYVFIYPNLMLNRYGP
**XP_013903967|Monoraphidium_neglectum_rev**	ASA.........................D..GRV......GGGRPAGYFFLYPNIMVNRYGP
**XP_005644270|Coccomyxa_subellipsoidea_C-169**	EER.............PQEGGEWDTITAN...RIE.....GDGRGPSYTFVYPNIMVNRYSL
**NC_024001.1|Bathycoccus_prasinos_corrected**	ENT........................NS...RLG....LGS...ATYAFAFPNVCLNRYGR
**EWM23896|Nannochloropsis_gaditana**	SFPPSTSAFSSSSPFPPRISPNHGGGSAT...RL......GK.A.ALYAHIYPGLMLNRYGP
**XP_002738379|Saccoglossus_kowalevskii**	VGS........................EE...RVC.....GD...AIYAHIFPNLMINRYGP
**XP_006820984|Saccoglossus_kowalevskii**	SNN.........................D..PRVS.....GN...VMLAHIYPNLILNRYGP
**XP_002737044|Saccoglossus_kowalevskii**	LKS....................DDPKIEA..RI......G..KSSTYTFIYPNFMVSRYGP
**XP_002597329|Branchiostoma_floridae__Florida_lanc**	EQS......................TEGQ...RY......GQ.AGYIVANVFPNLAINRYGP
**XP_019618440|Branchiostoma_belcheri__Belcher's_la**	...................AKENDESTEG...QRY.....GE..GYTVANLFPNLAVNRYGP
**XP_019644608|Branchiostoma_belcheri__Belcher's_la**	DDN.........................D...NTLKRQRVGK.K.AVYSCIYPNLLVNRYGP
**XP_019644788|Branchiostoma_belcheri__Belcher's_la**	DDN.........................D...NTLKRQRIGS.K.AVYSCIYPNLLVNRYGP
**XP_002610312|Branchiostoma_floridae__Florida_lanc**	ADG.........................G...DSAKRQRIGT.K.AVYSCIYPNLLVNRYGP
**XP_002599795|Branchiostoma_floridae__Florida_lanc**	.SG...............SGEDDQSMTRQ...RI......GK.T.SIYGCVYPNLMINRYGP
**XP_019633636|Branchiostoma_belcheri__Belcher's_la**	EDD.........................QSMTRQR....IGK.T.SIYGCVYPNLMINRYGP
**XP_004333794|Acanthamoeba_castellanii_str._Neff**	.QG..............TNNQMEEQLLQE...RV......GK.QGAHYAFLYPNFMINRYGN
**GAQ84770|Klebsormidium_flaccidum**	RKV.........................D...EIR....ADAPQ.ELYTLVYPNLLINRYGN

```
                                                  350          360        370
                                                    .            .          .
BAC77698|Atriplex_nummularia                      WMTTMHIVPL...GPRKCKLVVDYYI.EKSKLDD............................
AGC13074|Atriplex_semibaccata                     WMTTMHIVPL...GPRKCKLVVDYYI.EKSKLDD............................
AHH24260|Atriplex_amnicola                        WMTTMHIVPL...GPRKCKLVVDYYI.EKSKLDD............................
AFG28558|Atriplex_canescens__four-winged_saltbush WMTTMHIVPL...APRKCKLVVDYYI.EKSKLDD............................
Q9LKN0|Atriplex_hortensis                         WMTTMHIVPL...GPRKCKLVVDYYI.EKSKLDD............................
AAL92561|Atriplex_prostrata                       WMTTMHIVPL...GPRKCKLVVDYYI.EKSKLDD............................
O04121|Spinacia_oleracea__spinach_                WMTTMHIHPL...GPRKCKLVVDYYI.ENSMLDD............................
ABG34274|Ophiopogon_japonicus                     WMTTMHIHPL...GPRKCKLVVDYYI.ENSMWDD............................
AEW31326|Haloxylon_persicum                       WMTTMHVQPL...GLRKCKLVVDYYL.EESKLGD............................
ACX47904|Haloxylon_ammodendron                    WMTTMHVQPL...GLRKCKLVVDYYI.EESKLGD............................
AJF98574|Salicornia_bigelovii                     WMTTMHVHPI...AQRKCKLVVDYYI.EKSMLDD............................
AAV91779|Salicornia_europaea                      WMTTMHVHPI...AQRKCKLVVDYYI.EKSMLDD............................
AFW04225|Suaeda_maritima                          WMTTMHVQPI...AQRKCKLVVDYYI.EDSLLDN............................
AAM43920|Suaeda_liaotungensis                     WMTTMHVQPI...AQRKCKLVVDYYI.EDSLLDN............................
XP_010682183|Beta_vulgaris_subsp._vulgaris__sugar WMTTMHVVPM...GQRKCKLVVDYYL.EKAMLDD............................
AHYPO_002206-RA|Amaranthus_hypochondriacus_439    WMTTMHIVPL...GPRKCKLVVDYYL.DKSMMDD............................
BAF93187|Amaranthus_tricolor                      WMTTMHIVPL...GPRKCKLVVDYYL.DKSMMND............................
XP_012828802|Erythranthe_guttata__spotted_monkey_ WMDTNLVLPL...GPKKCQVIFDYFL.DASLTDN............................
Migut.J00779.1|Mimulus_guttatus                   WMDTNLVLPL...GPKKCQVIFDYFL.DASLTDN............................
EYU18199|Erythranthe_guttata__spotted_monkey_flow WMETNLVLPL...GPKKCHVVFDYFL.ETSLTVN............................
KQ992415.1|Dorcoceras_hygrometricum_corrected     WMDTNLVLPL...GPRRCKVVFDYFL.DASLKDD............................
XP_011094129|Sesamum_indicum__sesame_             WMDTNLVLPL...GPRKCQVIFDYFL.DAYLKDD............................
XP_015080536|Solanum_pennellii__Lycopersicon_penn WMDTNLVLPQ...GPRKCLVIFDYFL.DSSVKGD............................
XP_004242785|Solanum_lycopersicum__Lycopersicon_e WMDTNLVLPQ...GPRKCLVIFDYFL.DSSVKGD............................
NP_001275051|Solanum_tuberosum__potato_           WMDTNLVLPQ...GPRKCLVIFDYFL.DSSLKGD............................
XP_016556147|Capsicum_annuum                      WMDTNLVLPQ...GPRKCLVIFDYFL.DSSLKGD............................
ACR15118|Lycium_barbarum                          WMDTNLVLPQ...GPRKCLVIFDYFL.DASLKGD............................
XP_009757252|Nicotiana_sylvestris__wood_tobacco_  WMDTNLVLPQ...GPRKCLVIFDYFL.DASLKGD............................
XP_016460838|Nicotiana_tabacum_(common_tobacco)   WMDTNLVLPQ...GPRKCLVIFDYFL.DASLKGD............................
OIT26339|Nicotiana_attenuata                      WMDTNLVLPQ...GPRKCLVIFDYFL.DASLKGD............................
XP_009601434|Nicotiana_tomentosiformis            WMDTNLVLPQ...GPRKCLVIFDYFL.DASLKGD............................
XP_016508540|Nicotiana_tabacum_(common_tobacco)   WMDTNLVLPQ...GPRKCLVIFDYFL.DASLKGD............................
XP_015081828|Solanum_pennellii__Lycopersicon_penn WMDTNLVLPQ...GPRKCLVIFDYFL.DSSHKSD............................
XP_004243034|Solanum_lycopersicum__Lycopersicon_e WMDTNLVLPQ...GPRKCLVIFDYFL.DSSHKSD............................
CDP08949|Coffea_canephora                         WMDTNLVVPL...GPRKCQVIFDYFL.DTSLKDD............................
XP_019182381|Ipomoea_nil__Japanese_morning_glory_ WMDTNLVVPL...GPRKCQVIFDYFL.DTSLKGD............................
XP_010433050|Camelina_sativa__false_flax_         WMDTNLVVPL...GPRRCKVVFDYFL.DPSLKDD............................
XP_010436340|Camelina_sativa_false_flax_corr      WMDTNLVVPL...GPRRCKVVFDYFL.DPSLKDD............................
XP_010438247|Camelina_sativa__false_flax_         WMDTNLVVPL...GPRRCKVIFDYFL.DPSLKDD............................
XP_010447794|Camelina_sativa__false_flax_         WMDTNLVVPL...GPRRCKVIFDYFL.DPSLKDD............................
XP_006285614|Capsella_rubella                     WMDTNLVVPL...GPRRCKVIFDYFL.DPSLKDD............................
Cagra.0268s0015.1|Capsella_grandiflora            WMDTNLVVPL...GPRRCKVIFDYFL.DPSLKDD............................
Bostr.7867s0836.1|Boechera_stricta                WMDTNLVVPL...GPRRCKVIFDYFL.DPSLKDD............................
NP_194718|Arabidopsis_thaliana__thale_cress_      WMDTNLVLPL...GPRKCKVVFDYFL.DPSLKDD............................
XP_002869406|Arabidopsis_lyrata_subsp._lyrata     WMDTNLVRPL...GPRKCKVVFDYFL.DPSLKDD............................
Araha.6052s0002.1|Arabidopsis_halleri             WMDTNLVLPL...GPRKCKVVFDYFL.DPTLKDD............................
XP_006412796|Eutrema_salsugineum                  WMDTNLVIPL...GPRKCKVVFDYFL.DPSLKDD............................
KFK29548|Arabis_alpina__gray_rockcress_           WMDTNLVIPL...GSRKCKVVFDYFL.DPCLKDD............................
KFK40841|Arabis_alpina__gray_rockcress_           ...TDMVEP......................................................
XP_018481725|Raphanus_sativus__radish_            WMDTNLVIPL...GPRKCKVVFDYFL.DPSLKDD............................
XP_018435688|Raphanus_sativus__radish_            WMDTNLVIPL...GPRKCKVVFDYFL.DPSLKDD............................
XP_013705395|Brassica_napus__rape_                WMDTNLVIPL...GPRKCKVVFDYFL.DPSLKDD............................
XP_013596413|Brassica_oleracea_var._oleracea      WVDTNLVIPL...GPRKCKVVFDYFL.DPSLKDD............................
XP_009137872|Brassica_rapa__field_mustard_        WMDTNLVIPL...GPRKCKVVFDYFL.DPSLKDD............................
XP_013738248|Brassica_napus__rape_                WMDTNLVIPL...GPRKCKVVFDYFL.DPSLKDD............................
XP_010541096|Tarenaya_hassleriana                 WMDTNLVIPL...GPRKCKVVFDYFL.EPFLKED............................
Kaladp0809s0111.1|Kalanchoe_fedtschenkoi          WMDTNLVLPL...GHRKCRVVFDYFL.EASLMDD............................
Kalax.0333s0026.1|Kalanchoe_laxiflora_corr_462aa  WMDTNLVLPL...GHRKCRVVFDYFL.EASLMDD............................
Kalax.0012s0114.1|Kalanchoe_laxiflora_corr_462aa  WMDTNLVLPL...GHRKCRVVFDYFL.EASLMDD............................
XP_010667950|Beta_vulgaris_subsp._vulgaris__sugar WMDTNLVVPL...GPRKCQVVFDYFL.DMSLKDD............................
AH021158-RA|Amaranthus_hypochondriacus_421aa-corr WMDTNLIVPL...GPRKCKVVFDYFL.DSSLKDD............................
XP_021866412|426|Spinacia_oleracea_(spinach)      WMDTNLVIPL...GPRKCQVVFDYFL.DASLKDD............................
BAG74777|Hordeum_vulgare_subsp._vulgare__domestic WMDTNLAVPL...DATRCKVVFDYFL.DKSLLDD............................
AKZ66518|Triticum_monococcum_subsp._aegilopoides  ...............................................................
FAOM01578495|Triticum_aestivum_UpdateAMA02065     WMDTNLAVPL...DATRCKVVFDYFL.DESLLDD............................
EMT07994|Aegilops_tauschii_                       WMDTNLAVPL...DATRCKVVFDYFL.NESLLDD............................
ACZ92178|Triticum_turgidum_subsp._durum__durum_wh WMDTNLAVPL...DATRCKVVFDYFL.DESLLDD............................
KD266899|Triticum_urartu_UpdateEMS46986           WMDTNLAVPL...DATRCKVVFDYFL.DESLLDD............................
ABV64740|Leymus_chinensis                         WMDTNLAVPL...DATRCKVIFDYFL.DESLLDD............................
Brast07G193900.1|Brachypodium_stacei              WMDTNLAVPL...DATRCKVIFDYFL.DESLLDD............................
XP_003563491|Brachypodium_distachyon__stiff_brome WMDTNLAVPL...DATRCKVVFDYFL.DESLLDD............................
XP_006657294|Oryza_brachyantha__malo_sina_        WMDTNLVVPL...DATRCKVIFDYFL.DQSLLDD............................
XP_015643170|Oryza_sativa_Japonica_Group__Japanes WMDTNLVVPL...DATRCKVIFDYFL.DKSLMDD............................
EEC81252|Oryza_sativa_Indica_Group__long-grained_ WMDTNLVVPL...DATRCKVIFDYFL.DKSLMDD............................
XP_002437525|Sorghum_bicolor__sorghum_            WMDTNLAVPL...DSTSCKVVFDYFL.DKSLLDD............................
NP_001105926|Zea_mays                             WMDTNLAVPL...DSTRCKVVFDYFL.DKSLLDD............................
Pavir.Db00260.1|Panicum_virgatum_CORR_316f        WFH......L...AHYFCSTTF.FY........DD............................
LWDX02005496.1|Dichanthelium_oligosanthes_update_ WMDTNLAVPL...GSTKCKVVFDYFL.DKSLLDD............................
Sevir.4G257300.1|Setaria_viridis                  WMDTNLAVPL...GSTKCKVVFDYFL.DKSLLDD............................
XP_004966128|Setaria_italica__foxtail_millet_     WMDTNLAVPL...GSTKCKVVFDYFL.DKSLLDD............................
Oropetium_20150105_16264A|Oropetium_thomaeum      .......VLS...GE................................................
Pahal.D00004.1|Panicum_hallii                     WMDTNLAVPL...GSTKCKVVFDYFL.DKSLLDD............................
Pavir.J39646.1|Panicum_virgatum_Corr_363f         WMDTNLAVPL...GSTKCKVIFDYYL.DMSLLDD............................
OAY65202|Ananas_comosus__pineapple_               WMDTNLAIPL...GPTKCQVIFDYFL.DPSLADD............................
XP_009410214|Musa_acuminata_subsp._malaccensis__w WMDTNLVVPV...TTTKCQVIFDYFL.DVSLVDD............................
XP_010927268|Elaeis_guineensis__African_oil_palm_ WMDTNLVVPL...GLTKCQVIFDYFL.DYSLLDD............................
XP_008801514|Phoenix_dactylifera__date_palm_      WLDTNLVIPL...GSTNCQVIFDYFL.DHSLLDD............................
KMZ72255|Zostera_marina                           WMDTNLVVPL...GPQNCQVIIDYFL.DPYIKVD............................
JAT51600|Anthurium_amnicola                       WMDTNLVLPL...GAHKCLVVFDYFL.DSSLQDE............................
Spipo5G0003400|Spirodela_polyrhiza                WMDTNLVVPE...GARRCRVIFDYFL.DAALQED............................
XP_006845775|Amborella_trichopoda                 WMDTNLVLPL...GFDKCQVIFDYFL.DCSLKDD............................
AFP19450|Camellia_sinensis                        WMDTNLVLPI...GSRKCQVIFDYFL.DTSLKED............................
XP_017254907|Daucus_carota_subsp._sativus         WMDTNLVLPL...GPRKCQVIFDYFL.DPNLKDD............................
ABX57826|Chrysanthemum_lavandulifolium            WM.............................................................
LEKV01000036.1|Cynara_cardunculus_update_KVI11819 WMDTNLVLPL...GPRRCKVIFDYFL.DASLKDD............................
XP_008443631|Cucumis_melo__muskmelon_             WMDTNLVLPL...GPRKCLVVFDYFL.EASFKND............................
XP_004139149|Cucumis_sativus__cucumber_           WMDTNLVLPL...GPRKCLVVFDYFL.EASFKND............................
XP_008351406|Malus_domestica__apple_              WMDTNLVLPL...GPRKCQVRFDYFI.EASLKDD............................
XP_008391562|Malus_domestica__apple_              WMDTNLVLPL...GPRKCQVRFDYFI.EASLKDD............................
XP_009343851|Pyrus_x_bretschneideri__Chinese_whit WMDTNLVLPL...GQRKCQVRFDYFI.EASLKDD............................
AER10510|Pyrus_betulifolia_corr_405aa             WMDTNLVLPL...GQRKCQVRFDYFI.EASLKDD............................
XP_008223853|Prunus_mume__Japanese_apricot_       WMDTNLVLPL...GPRKCQVIFDYYI.EPSLMDN............................
ONI27175|Prunus_persica__peach_                   WMDTNLVLPL...GPRKCQVIFDYFI.EPSLMDN............................
```

```
XP_004301419|Fragaria_vesca_subsp._vesca         WMDTNLVIPL...GPRKCQVIFDYFL.EASVKDD............................
XP_015889685|Ziziphus_jujuba__common_jujube_     WMDTNLVLPL...GPRKCQVVFDYFL.EPSLKDD............................
NW_010360102.1|Morus_notabilis_corrected         WMDTNLVLPL...GPKKCQVVFDYFL.EPSLKDD............................
Lus10032689|Linum_usitatissimum                  WMDTNLVVPL...GPRKCRVIFDYFI.EHHLKDD............................
Lus10008571|Linum_usitatissimum_corrected        WMDTNLVVPL...GPRKCQVIFDYFI.EPHLKDD............................
XP_011020077|Populus_euphratica__Euphrates_poplar WMDTNLVLPL...GPRKCQVIFDYFI.EAHLKDD............................
XP_002308100|Populus_trichocarpa__Populus_balsami WMDTNLVLPL...GPRKCQVIFDYFI.EAHLKDD............................
SapurV1A.0198s0060.1|Salix_purpurea              WMDTNLVLPL...GPRKCHVIFDYFI.ETHLKDD............................
OAY46667|Manihot_esculenta__cassava_             WMDTNLVVPL...GPTKCQVIFDYFI.EGSHKDD............................
XP_002518256|Ricinus_communis__castor_bean_      WMDTNLVLPL...GPSKCQVIFDYFV.EADYKND............................
XP_012074438|Jatropha_curcas                     WMDTNLVLPL...GTSKCQVIFDYFI.EAYHKDD............................
XP_010046909|Eucalyptus_grandis                  WMDTNLVLPL...GPRKCRVIFDYFL.KDSLKDD............................
ABS71853|Eucalyptus_camaldulensis_Murray_red_gum_ WMDTNLVLPL...GPKKGCVIFDYFL.KDSLKDD............................
XP_006474292|Citrus_sinensis__sweet_orange_      WMDTNLVIPL...GPTRCEVVFDYFL.DGSLMDD............................
XP_006453220|Citrus_clementina_reviewed          WMDTNLVIPL...GPTRCEVVFDYFL.DGSLMDD............................
XP_017612508|Gossypium_arboreum                  WMDTNLAIPF...GPRKCLVVFDYFL.EASFKDD............................
XP_016686114|Gossypium_hirsutum__cotton_         WMDTNLAIPL...GPRKCLVVFDYFL.EASFKDD............................
XP_012459381|Gossypium_raimondii                 WMDTNLAIPL...GPRKCLVVFDYFL.EASFKDD............................
XP_016719040|Gossypium_hirsutum__cotton_         WMDTNLAIPL...GPRKCLVVFDYFL.EASFKDD............................
EOY05290|Theobroma_cacao__cacao_                 WMDTNLVIPL...GPRRCLVVFDYFL.EASLKDD............................
AWWV01015497-AWWV01004954_Corchorus_capsularis_39 WMDTNLAIPL...GPRRCLVVFDYFL.EASHKDD............................
OMO51681|Corchorus_olitorius                     WMDTNLAIPL...GPKRCLVVFDYFL.EASHKDD............................
GAV59711|Cephalotus_follicularis                 WMDTNLVIPL...GPRKCRVVFDYFL.EGSLKDD............................
XP_018807260|Juglans_regia__English_walnut_      WMDTNLVLPL...GPRKCKVVFDYFI.EASRKDD............................
XP_019078540|Vitis_vinifera__wine_grape_         WMDTNLVLPL...GPRTCKVVFDYFL.EASLKDD............................
Aqcoe7G261800.1|Aquilegia_coerulea               WMDTNLVLPL...GPNKCQVIFDYFL.EPSLKDD............................
XP_010276721|Nelumbo_nucifera__sacred_lotus_     WMDTNLVLPL...GYRKCQVIFDYFL.ESSLKDD............................
XP_016201893|Arachis_ipaensis                    WMDTNLVLPL...GPNKCQVVFDYYL.EHSLKHD............................
XP_015973237|Arachis_duranensis                  WMDTNLVLPL...GPNKCQVVFDYYL.EHSLKHD............................
Tp57577_TGAC_v2_mRNA5577|Trifolium_pratense      WMDTNLVLPL...GPNKCQVIFDYYL.EPSLQVD............................
GAU38011|Trifolium_subterraneum                  WMDTNLVLPL...GPNKCQVIFDYYL.EPSLQVD............................
XP_003610028|Medicago_truncatula__barrel_medic_  WMDTNLVLPL...GPSKCQVVFDYYL.EPSLQGD............................
XP_004507911|Cicer_arietinum__chickpea_          WMDTNLVLPL...GPNKCQVVFDYYL.EPSLQDD............................
XP_019463425|Lupinus_angustifolius__narrow-leaved WMDTNLVLPL...GPNKCQVVFDYYL.DHSLKDD............................
KYP52554|Cajanus_cajan__pigeon_pea_              WMDTNLVVPL...GPNKCQVIFDYYL.EHSLKDD............................
XP_003549280|Glycine_max__soybean_               WMDTNLVVPL...GPNKCQVIFDYYL.EHSLKDD............................
XP_007134857|Phaseolus_vulgaris_CHR1_corr_208aaf WMDTNLVVPL...GPNKCQVIFDYYL.ERSLKSI............................
XP_007154695|Phaseolus_vulgaris_CORR             WMDTNLVVPL...GPNKCKVIFDYYL.ERSLKND............................
XP_017410379|Vigna_angularis__adzuki_bean_       WMDTNLVVPL...GPNKCQVIFDYYL.ERSLKND............................
XP_014508581|Vigna_radiata_var._radiata__mung_bea WMDTNLVVPL...GPNKCQVIFDYYL.ERSLKND............................
AUSU01000986.1|Genlisea_aurea_corrected-f        WMDTNLVLPM...GPKRCQVVFDYFL.EPSFKDD............................
OAE24413|CORR_Marchantia_polymorpha              WMDTNLVLPV...SATQCRVVINWFL.EPDFPND............................
Sphfalx0064s0059.1|Sphagnum                      WMDTNLVLPI...TESQCRVIFDWFL.DPSRVDD............................
XP_001752587|Physcomitrella_patens               WMDTNLVIPI...SMSECRVIFDWFL.EPSLVHD............................
XP_002508933|Micromonas_sp._RCC299               WMDVNVVLPT...GPNECMVLFDYFI.RADATGAT...........................
XP_003061830|Micromonas_pusilla_CCMP1545         WLDTNWVIPT...GAETCKVVFEYYL.EKGVEGAT...........................
XP_005645368|Coccomyxa                           WMDTNLVLPD...GPRRCTVHFEYWL.EQSLVHD............................
ADIC01002027.1|Chlorella_variabilis_corrected_pre WLDANYVVPL...AANRCKVLFDYFL.DPSLAGD............................
XP_013903967|Monoraphidium_neglectum_rev         WMDTNVVVPADSRSPDRCRVRFDWWL.QADALERLLAGGGGVQQQGREGEGGRSGDDGGGAV
XP_005644270|Coccomyxa_subellipsoidea_C-169      WMGTMRVLPL...GEKKTKVLMDYYV.HTSKVDD............................
NC_024001.1|Bathycoccus_prasinos_corrected       WLDTNVAFPNPA.DPNKCVVEFNWYI.DNTNHDEDDD.........................
EWM23896|Nannochloropsis_gaditana                WMDLNILFPV...SSSSCDLIFDYFL.EETFIAEKLEKDGE.....................
XP_002738379|Saccoglossus_kowalevskii            WIDTNVALPL...THNTCMIVFDYFL.EEDYIQQKSE.........................
XP_006820984|Saccoglossus_kowalevskii            WLDTNIVMPV...SANECVIIYDYYL.LQSFVDTT...........................
XP_002737044|Saccoglossus_kowalevskii            WFETNMVVPH...GHNHAEFVYDFYL.DKSFVEKQTE.........................
XP_002597329|Branchiostoma_floridae__Florida_lanc WLDTNLLLPL...ATDRTRIVMDYFL.EEDFVKNIGD.........................
XP_019618440|Branchiostoma_belcheri__Belcher's_la WLDTNLLLPL...AADRTRIVMDYFL.EEDFVKNTSE.........................
XP_019644608|Branchiostoma_belcheri__Belcher's_la WLDINILLPL...GPDRSLMIYDFYL.KESFAEEMSE.........................
XP_019644788|Branchiostoma_belcheri__Belcher's_la WLDINILLPL...GPDRSLMIYDFYL.KESFAEEMSE.........................
XP_002610312|Branchiostoma_floridae__Florida_lanc WLDINIVLPL...GPDRSLMIYDFYL.KETFANEMSE.........................
XP_002599795|Branchiostoma_floridae__Florida_lanc WLDTNIALPL...THNTTEVIYDYWL.AEDFAQTMGE.........................
XP_019633636|Branchiostoma_belcheri__Belcher's_la WLDTNIALPL...THDTTEVIYDYWL.AEEFAQTLRGE........................
XP_004333794|Acanthamoeba_castellanii_str._Neff  LMDTNRAIPI...SVDETLVQFDYFMLKDGDTDQENQ.........................
GAQ84770|Klebsormidium_flaccidum                 WVDSFWVIPT...GPTSCHVTVDYWL.APQDAAE............................
```

```
                                                            380       390       400       410
BAC77698|Atriplex_nummularia                          ..................KDYIEKGIAINDNVQKEDVVLCESVQKGLETPAYRS........
AGC13074|Atriplex_semibaccata                         ..................KDYIEKGIAINDNVQKEDVVLCESVQKGLETPAYRS........
AHH24260|Atriplex_amnicola                            ..................KDYIEKGIAINDNVQKEDVVLCESVQKGLETPAYRS........
AFG28558|Atriplex_canescens__four-winged_saltbush     ..................KDYIEKGIAINDNVQKEDVVLCESVQKGLETPAYRS........
Q9LKN0|Atriplex_hortensis                             ..................KDYIEKGIAINDNVQKEDVVLCESVQKGLETPAYRS........
AAL92561|Atriplex_prostrata                           ..................KDYIEKGIAINDNVQKEDVVLCESVQKGLETPAYRS........
O04121|Spinacia_oleracea__spinach_                    ..................KDYIEKGIAINDNVQREDVVLCESVQRGLETPAYRS........
ABG34274|Ophiopogon_japonicus                         ..................KDYIEKGIAINDNVQREDVVLCESVQRGLETPAYRS........
AEW31326|Haloxylon_persicum                           ..................KEYIEKGIAINDNVQAEDKVLCESVQRGLETPAYSS........
ACX47904|Haloxylon_ammodendron                        ..................KEYIEKGIAINDNVQAEDKVLCESVQRGLETPAYSS........
AJF98574|Salicornia_bigelovii                         ..................KEYIDRGIAINDNVQREDKVLCERVQNGLETPAYRS........
AAV91779|Salicornia_europaea                          ..................KEYIDRGIAINDNVQREDKVLCESVQNGLETPAYRS........
AFW04225|Suaeda_maritima                              ..................KDYIEKGIAINDNVQKEDKVLCESVQKGLETPAYRS........
AAM43920|Suaeda_liaotungensis                         ..................KDYIEKGIAINDNVQKEDKVLCESVQKGLETPAYRS........
XP_010682183|Beta_vulgaris_subsp._vulgaris__sugar     ..................KAYIDKGIAINDNVQKEDKVLCESVQRGLETPAYRS........
AHYPO_002206-RA|Amaranthus_hypochondriacus_439        ..................KPYIEKSIEINDNVQKEDVVLCESVQKGLETPAYRS........
BAF93187|Amaranthus_tricolor                          ..................KPYIEKSIMINDNVQKEDVVLCESVQRGLETPAYRS........
XP_012828802|Erythranthe_guttata__spotted_monkey_     ..................VAFIGKCLEDSERVQVEDAFLCKSVQEGLESPAYNM........
Migut.J00779.1|Mimulus_guttatus                       ..................VAFIGKCLEDSERVQVEDAFLCKSVQEGLESPAYNM........
EYU18199|Erythranthe_guttata__spotted_monkey_flow     ..................IDFIEKSSETSHGVQEEDMSISNSVQKGLESPAYHV........
KQ992415.1|Dorcoceras_hygrometricum_corrected         ..................VAFIEKSLEDSERVQLEDITLSEAVQRGLESPAYNV........
XP_011094129|Sesamum_indicum__sesame_                 ..................SAFIEKSLEDSEKVQDEDIILCKAVQQGLESPAYDV........
XP_015080536|Solanum_pennellii__Lycopersicon_penn     ..................ESFIAQSLEDSETVQMEDIKLCEGVQRGLESPAYCS........
XP_004242785|Solanum_lycopersicum__Lycopersicon_e     ..................ESFIAQGLKDSETVQMEDIKLCEGVQRGLESPAYCS........
NP_001275051|Solanum_tuberosum__potato_               ..................ESFIAQSLEDSETVQMEDIKLCEAVQRGLESPAYCS........
XP_016556147|Capsicum_annuum                          ..................ESFIEQSLKDSETVQMEDIKLCEAVQRGLESPAYCS........
ACR15118|Lycium_barbarum                              ..................ESFIAQSLQDSETVQIEDIKLCEAVQRGLESPAYRT........
XP_009757252|Nicotiana_sylvestris__wood_tobacco_      ..................ESFIAQSLQDSETVQMEDIKLCEAVQRGLESPAYNS........
XP_016460838|Nicotiana_tabacum_(common_tobacco)       ..................ESFIAQSLQDSETVQMEDIKLCEAVQRGLESPAYNS........
OIT26339|Nicotiana_attenuata                          ..................KSFIAQSLQDSETVQMEDIKLCEAVQRGLESPAYSS........
XP_009601434|Nicotiana_tomentosiformis                ..................ESFIAQSLQDSETVQIEDIKLCEAVQRGLESPAYSS........
XP_016508540|Nicotiana_tabacum_(common_tobacco)       ..................ESFIAQSLQDSETVQIEDIKLCEAVQRGLESPAYSS........
XP_015081828|Solanum_pennellii__Lycopersicon_penn     ..................ESFIAQSLEDSETVQIEDMKLCEAVQRGLESPAYCS........
XP_004243034|Solanum_lycopersicum__Lycopersicon_e     ..................ESFIAQSLEDSETVQIEDMKLCEAVQRGLESPAYCS........
CDP08949|Coffea_canephora                             ..................HDFVERSLKESEKVQVEDIRLCESVQRGIESPAYCC........
XP_019182381|Ipomoea_nil__Japanese_morning_glory_     ..................NEFIERSLEDSERVQMEDMSLCEGVQRGLESPAYVC........
XP_010433050|Camelina_sativa__false_flax_             ..................EAFIKRSLEESDKVQMEDVVLCESVQRGLESQAYDK........
XP_010436340|Camelina_sativa_false_flax_corr          ..................EAFIKRSLEESDKVQMEDVVLCESVQRGLESQAYDK........
XP_010438247|Camelina_sativa__false_flax_             ..................EAFIKRSLEESDKVQMEDVVLCESVQRGLESQAYDK........
XP_010447794|Camelina_sativa__false_flax_             ..................EAFIKRSLEESDKVQMEDVVLCENVQRGLESQAYDK........
XP_006285614|Capsella_rubella                         ..................EAFIKRSLEESDKVQMEDGVLCESVQRGLESQAYEK........
Cagra.0268s0015.1|Capsella_grandiflora                ..................EAFIKRSLEESDKVQMEDGVLCESVQRGLESQAYEK........
Bostr.7867s0836.1|Boechera_stricta                    ..................EAFIKRSLEESDRVQMEDVVLCESVQRGLESQAYDK........
NP_194718|Arabidopsis_thaliana__thale_cress_          ..................EAFIKRSLEESDRVQMEDVMLCESVQRGLESQAYDK........
XP_002869406|Arabidopsis_lyrata_subsp._lyrata         ..................EAFIKRSLEESDRVQMEDVVLCESVQRGLESQAYDK........
Araha.6052s0002.1|Arabidopsis_halleri                 ..................EAFIKRSLEESDRVQVEDVVLCESVQRGIESQAYDK........
XP_006412796|Eutrema_salsugineum                      ..................EAFIERSLEESERVQMEDVALCESVQRGLESPAYDK........
KFK29548|Arabis_alpina__gray_rockcress_               ..................EAFIKRSLEESERVQMEDVVLCESVQRGLESVAYDK........
KFK40841|Arabis_alpina__gray_rockcress_               ..............................................................
XP_018481725|Raphanus_sativus__radish_                ..................EAFIKRSLEESERVQMEDVVLCENVQRGLESPAYDK........
XP_018435688|Raphanus_sativus__radish_                ..................EAFIKRSLEESERVQMEDVVLCENVQRGLESPAYDK........
XP_013705395|Brassica_napus__rape_                    ..................EAFIRRSLEESERVQMEDAVLCENVQRGLESPAYDK........
XP_013596413|Brassica_oleracea_var._oleracea          ..................EAFIRRSLEESERVQMEDAVLCENVQRGLESPAYDK........
XP_009137872|Brassica_rapa__field_mustard_            ..................EAFIRRSLEESKRVQMEDVVLCENVQRGLESPAYDK........
XP_013738248|Brassica_napus__rape_                    ..................EAFIRRSLEESKRVQMEDVVLCENVQRGLESPAYDK........
XP_010541096|Tarenaya_hassleriana                     ..................ESFIERSLEESERVQMEDIVLCENVQRGLESPAYVK........
Kaladp0809s0111.1|Kalanchoe_fedtschenkoi              ..................TAFIERSLDESEIVQNEDIKLSEGVQRGLESPAFSR........
Kalax.0333s0026.1|Kalanchoe_laxiflora_corr_462aa      ..................TAFIERSLDESEIVQNEDIKLSEGVQRGLESPAFSK........
Kalax.0012s0114.1|Kalanchoe_laxiflora_corr_462aa      ..................TAFIERSLDESEIVQNEDIKLSEGVQRGLESPAFSK........
XP_010667950|Beta_vulgaris_subsp._vulgaris__sugar     ..................EAFIKRSLKDSEEVQIEDIMLCEGVQRGLESPAYRT........
AH021158-RA|Amaranthus_hypochondriacus_421aa-corr     ..................KAFIERSLKDSEAVQIEDIMLCEGVQRGLESPAYET........
XP_021866412|426|Spinacia_oleracea_(spinach)          ..................KAFIERSLKDSEEVQIEDIMLCEGVQRGLESPAYNT........
BAG74777|Hordeum_vulgare_subsp._vulgare__domestic     ..................QDFINRSLKDSEQVQIEDIALCEGVQRGLASPAYGV........
AKZ66518|Triticum_monococcum_subsp._aegilopoides      ..............................................................
FAOM01578495|Triticum_aestivum_UpdateAMA02065         ..................QDFINRSLKDSEQVQIEDIALCEGVQRGLESPAYGV........
EMT07994|Aegilops_tauschii_                           ..................QDFIDRSLKDSEQVQIEDIALCEGVQRGLESPAYGV........
ACZ92178|Triticum_turgidum_subsp._durum__durum_wh     ..................QDFIDRSLKDSEQVQIEDIALCEGVQRGLESPAYGV........
KD266899|Triticum_urartu_UpdateEMS46986               ..................QDFIDRSLKDSEQVQIEDIALCEGVQRGLESPAYGV........
ABV64740|Leymus_chinensis                             ..................EGFIDRSLKDSEQVQIEDIALCEGVQRGLESPAYGV........
Brast07G193900.1|Brachypodium_stacei                  ..................QDFIDQSLKDSEQVQIEDIALCEGVQRGLESPAYGV........
XP_003563491|Brachypodium_distachyon__stiff_brome     ..................QDFIDQSLKDSEQVQIEDIALCEGVQKGLESPAYGV........
XP_006657294|Oryza_brachyantha__malo_sina_            ..................QKFIDRSLKDSEQVQMEDIALCEGVQRGLESPAYSV........
XP_015643170|Oryza_sativa_Japonica_Group__Japanes     ..................QNFIESSLKDSEQVQMEDIALCEGVQRGLESPAYSV........
EEC81252|Oryza_sativa_Indica_Group__long-grained_     ..................QNFIESSLKDSEQVQMEDIALCEGVQRGLESPAYSV........
XP_002437525|Sorghum_bicolor__sorghum_                ..................ESFIKKSLKDSEQVQIEDIALCEGVQRGLESPAYSV........
NP_001105926|Zea_mays                                 ..................QCFIKKSLEDSEQVQIEDIALCEGVQRGLESPAYSV........
Pavir.Db00260.1|Panicum_virgatum_CORR_316f            ..................QNFLERSLRDSEQVQMEDIALCEGVQRGLESPAYSI........
LWDX02005496.1|Dichanthelium_oligosanthes_update_     ..................QNFIERSLKDSEQVQMEDIALCEGVQRGLESPAYSV........
Sevir.4G257300.1|Setaria_viridis                      ..................QNFIERSLKDSEQVQMEDIALCEGVQRGLESPAYGV........
XP_004966128|Setaria_italica__foxtail_millet_         ..................QNFIERSLKDSEQVQMEDIALCEGVQRGLESPAYGV........
Oropetium_20150105_16264A|Oropetium_thomaeum          ..................QNFTEKSLKDSEQVQMEDIALCEGVQRGLESLAYSV........
Pahal.D00004.1|Panicum_hallii                         ..................QNFIERSLRDSEQVQMEDIALCEGVQLGLKSPVYSI........
Pavir.J39646.1|Panicum_virgatum_Corr_363f             ..................QNFIERSLRDSEQVQ.............................
OAY65202|Ananas_comosus__pineapple_                   ..................KAFIERSLEDSERVQIEDIALCEGVQRGLESPAYDS........
XP_009410214|Musa_acuminata_subsp._malaccensis__w     ..................KVFIERSLEESERVQLQDIVLCEGVQRGLESPAYCS........
XP_010927268|Elaeis_guineensis__African_oil_palm_     ..................KVFIERSLKESERVQMEDIALCEGVQRGLESPAYCS........
XP_008801514|Phoenix_dactylifera__date_palm_          ..................KVFIERSLKESERVQVEDIALCEGVQRGLESPAYCS........
KMZ72255|Zostera_marina                               ..................ANFITRSLEESEKIQMEDIGLCEGVQKGLESPLYST........
JAT51600|Anthurium_amnicola                           ..................NAFIKRSLEESEKVQMEDIQLCEGVQKGLESPAFCS........
Spipo5G0003400|Spirodela_polyrhiza                    ..................GGFIERSLEESERVQMEDVELCEGVQKGLESPAYCS........
XP_006845775|Amborella_trichopoda                     ..................KAFIERSLEESERVQMEDIILCEGVQKGLESPAYCS........
AFP19450|Camellia_sinensis                            ..................IAFIESSLEDSERVQMEDIILCEGVQRGLESPAYCS........
XP_017254907|Daucus_carota_subsp._sativus             ..................KEFIESSLKDSENVQDEDTFLCEAVQKGLESPAYSS........
ABX57826|Chrysanthemum_lavandulifolium                ..............................................................
LEKV01000036.1|Cynara_cardunculus_update_KVI11819     ..................EAFVAKSLEDSEQVQMEDIMLCESVQRGLESPAYDN........
XP_008443631|Cucumis_melo__muskmelon_                 ..................DSFIQQSLEDSESVQNEDIILCEGVQKGLESPAYKF........
XP_004139149|Cucumis_sativus__cucumber_               ..................DSFIQLSLEDSESVQNEDIILCEGVQKGLESPAYKF........
XP_008351406|Malus_domestica__apple_                  ..................RDFIERSLKG.............GCKK.................
XP_008391562|Malus_domestica__apple_                  ..................RDFIERSLKDSERVQTEDVMLCEGVQRGLESPAYNI........
XP_009343851|Pyrus_x_bretschneideri__Chinese_whit     ..................TDFIERSLKDSERVQMEDVMLCEGVQRGLESPTYNI........
AER10510|Pyrus_betulifolia_corr_405aa                 ..................TDFIERSLKDSERVQMEDVMLCEGVQRGLESPAYNI........
XP_008223853|Prunus_mume__Japanese_apricot_           ..................RDFIERSLKDSERVQLEDVMLCEGVQRGLESPAYDI........
ONI27175|Prunus_persica__peach_                       ..................KDFIERSLKDSERVQVEDVVLCEGVQRGLESPAYDI........
```

```
XP_004301419|Fragaria_vesca_subsp._vesca            ..................SDYIERSLNESEKVQMEDIVLCEGVQRGLESPAYNI........
XP_015889685|Ziziphus_jujuba__common_jujube_        ..................RAFIEKSLKDSERVQMEDIMLCEGVQRGLESSAYSV........
NW_010360102.1|Morus_notabilis_corrected            ..................KDFINRSLQDSERVQMEDVALCEGVQRGLESPAYCF........
Lus10032689|Linum_usitatissimum                     ..................KSYIEQSIIDSEKVQMEDSILCEGVQRGLESPAYCT........
Lus10008571|Linum_usitatissimum_corrected           ..................KSYIEQSVINSEKVQMEDIILCEGVQRGLESPAYCT........
XP_011020077|Populus_euphratica__Euphrates_poplar   ..................KDFIERSLVDSERVQIEDIVLCEGVQRGLETPAYCS........
XP_002308100|Populus_trichocarpa__Populus_balsami   ..................KDFIERSLVDSERVQIEDIVLCEGVQRGLETPAYCS........
SapurV1A.0198s0060.1|Salix_purpurea                 ..................KDFIERSLVDSERVQIEDTKLCEGVQRGLETPAYSS........
OAY46667|Manihot_esculenta__cassava_                ..................KAFIEKSLEDSERVQMEDVMLCEGVQRGLETPAYCS........
XP_002518256|Ricinus_communis__castor_bean_         ..................KTFIDRSLIDSERVQMEDIMLCEGVQRGLESPAYCR........
XP_012074438|Jatropha_curcas                        ..................KAFIEQSLVDSERVQMEDILLCEGVQRGLHSPAYSV........
XP_010046909|Eucalyptus_grandis                     ..................KNFVERSLQDSERVQMEDIVLCEGIQKGLESPAYCS........
ABS71853|Eucalyptus_camaldulensis_Murray_red_gum_   ..................KNFVERSLQDSERVQMEDIVLCEGIQKGLESPAYCS........
XP_006474292|Citrus_sinensis__sweet_orange_         ..................KAFIEQSLKDSEQVQIEDIILCEGVQRGLESPAYCS........
XP_006453220|Citrus_clementina_reviewed             ..................KAFIEQSLKDSEQVQIEDIILCEGVQRGLESPAYCS........
XP_017612508|Gossypium_arboreum                     ..................KAFIEGSLADSEKVQMEDIRLCEGVQKGIESPAYST........
XP_016686114|Gossypium_hirsutum__cotton_            ..................KAFIERSLADSEKVQMEDIRLCEGVQKGIESPAYST........
XP_012459381|Gossypium_raimondii                    ..................KAFIERSLADSEKVQMEDIRLCEGVQKGIESPAYST........
XP_016719040|Gossypium_hirsutum__cotton_            ..................KAFIERSLADSEKVQMEDIRLCEGVQKGIESPAYST........
EOY05290|Theobroma_cacao__cacao_                    ..................KAFIERSLVDSERVQMEDITLCEGVQRGIESPAYCS........
AWWV01015497-AWWV01004954_Corchorus_capsularis_39   ..................KAFIERSLVDSERVQMEDITLCEGVQRGLESPAYCT........
OMO51681|Corchorus_olitorius                        ..................KAFIERSLVDSERVQMEDITLCEGVQRGLESPAYCS........
GAV59711|Cephalotus_follicularis                    ..................QAFVERCLEDSERVQVEDIELCEGVQRGLESPAYDR........
XP_018807260|Juglans_regia__English_walnut_         ..................KAFIEKSLEDSERVQIEDIVLCEGVQKGLESPAYST........
XP_019078540|Vitis_vinifera__wine_grape_            ..................KAFIERSLEDSERVQMEDIILCEGVQRGLESPAYCS........
Aqcoe7G261800.1|Aquilegia_coerulea                  ..................KAFIEKSLEESEKVQIEDIALCEGVQKGLESPAYCT........
XP_010276721|Nelumbo_nucifera__sacred_lotus_        ..................EAFIERSLEESERVQMEDIALCEGVQKGLESPAYYR........
XP_016201893|Arachis_ipaensis                       ..................KDFIELSLQDSEKVQIEDIVLCEGVQKGLQSPAYCV........
XP_015973237|Arachis_duranensis                     ..................KDFIELSLQDSEKVQIEDIVLCEGVQKGLQSPAYYV........
Tp57577_TGAC_v2_mRNA5577|Trifolium_pratense         ..................KDFIEKSLQDSEKVQIEDIVLCEGVQKGLQSPAYSV........
GAU38011|Trifolium_subterraneum                     ..................KDFIEKSLQDSEKVQIEDIVLCEGVQKGLQSPAYSV........
XP_003610028|Medicago_truncatula__barrel_medic_     ..................RDFIEKSLQDSEKVQIEDIVLSEGVQKGLQSPAYSV........
XP_004507911|Cicer_arietinum__chickpea_             ..................TGFIEKSLQDSEKVQIEDILLCEGVQKGLQSPAYSV........
XP_019463425|Lupinus_angustifolius__narrow-leaved   ..................KDYIENSLQDSEIVQIEDIVLCEGVQKGLQSPAYRV........
KYP52554|Cajanus_cajan__pigeon_pea_                 ..................TDFIEKSLQDSEEVQIEDIVLCQGVQKGLQSPAYSV........
XP_003549280|Glycine_max__soybean_                  ..................KDFIEKSLQDSEKVQIEDIVLCEGVQKGLQSPAYRV........
XP_007134857|Phaseolus_vulgaris_CHR1_corr_208aaf    ..................VFFLYQLFTS..................................
XP_007154695|Phaseolus_vulgaris_CORR                ..................RDFQEKSLRDSEKVQIEDIVLCEGVQKGLQSPAYSV........
XP_017410379|Vigna_angularis__adzuki_bean_          ..................TDFVEKSLQDSEKVQLEDVVLCEGVQKGLQSPAYSV........
XP_014508581|Vigna_radiata_var._radiata__mung_bea   ..................TDFVEKSLQDSEKVQIEDVVLCEGVQKGLQSPAYSV........
AUSU01000986.1|Genlisea_aurea_corrected-f           ..................KQFIDGSLKESEIVQEEDIALCTAVQRGLDSPAYNV........
OAE24413|CORR_Marchantia_polymorpha                 ..................EALIKSSIETSDVVQQEDIVLCESVQEGLQSPAYDV........
Sphfalx0064s0059.1|Sphagnum                         ..................KEFIETSIEDSERVQIEDITLCEDVQDGLKSPAYDV........
XP_001752587|Physcomitrella_patens                  ..................KEFIDSSIADSEVVQKEDIILCEDVQNGLSSPAYDV........
XP_002508933|Micromonas_sp._RCC299                  ..................DAFVNESLRASDAVQSEDKWLCEKVQGGLESPGYGT........
XP_003061830|Micromonas_pusilla_CCMP1545            ..................EAFIEKSLAASDRIQREDTTLCEKVQEGVRSPGYGV........
XP_005645368|Coccomyxa                              ..................SSLIDSSLADSDQVQQEDIALCEAVQRGLHSPAYDT........
ADIC01002027.1|Chlorella_variabilis_corrected_pre   ..................AGFIEESLAASHRVQLEDVALCESVQRGLASAAYDT........
XP_013903967|Monoraphidium_neglectum_rev            EVEEAVAGIEFTHSGAAVLPFVRESLASSHQVQVEDIGLCEAVQLGLEEPAYGV........
XP_005644270|Coccomyxa_subellipsoidea_C-169         ..................KEWIDQVVMGEDIVQREDMHMCDLVQKGLASPAYDV........
NC_024001.1|Bathycoccus_prasinos_corrected          .................EEVFIKQSLLASRVVQDEDENLCASVQKGLRSKVASSSSSSSSSS
EWM23896|Nannochloropsis_gaditana                   ...............AGLRTYVEESLIASDEVQREDTYLCENVQAGLESGGYDR........
XP_002738379|Saccoglossus_kowalevskii               ...............EDLQNILDTSFTASDKVQQEDIFLCEGVQRGLESSAYDL........
XP_006820984|Saccoglossus_kowalevskii               .............SPRYLPKYIDDSILDSDKLQREDNMVCEGTQKGLQSSGYEV........
XP_002737044|Saccoglossus_kowalevskii               ...............EEVQRYVDESLVAANQVQGEDTEICEGVQRGMSSIAFES........
XP_002597329|Branchiostoma_floridae__Florida_lanc   ...............KELAEFVETSLKDSDQVQKEDNDICESVQRGLTSSAYDV........
XP_019618440|Branchiostoma_belcheri__Belcher's_la   ...............KELADFIETSLKDSDQVQKEDNDICENVQRGLLSSAYDV........
XP_019644608|Branchiostoma_belcheri__Belcher's_la   ...............TELAEFIQQSLKDSDQVQQEDTRVCEGVQRGMESTAFDV........
XP_019644788|Branchiostoma_belcheri__Belcher's_la   ...............TELAEFIEQSLKDSDQVQQEDTRVCEGVQRGMESTAFDV........
XP_002610312|Branchiostoma_floridae__Florida_lanc   ...............TELANFIDQSLQDSDQVQQEDTRVCEGVQRGMESTAFDV........
XP_002599795|Branchiostoma_floridae__Florida_lanc   ...............TELQEFVQTSLRESDQVQQEDTFICERVQKGLKSSAYDV........
XP_019633636|Branchiostoma_belcheri__Belcher's_la   ...............AELQEFVQTSLTESDKVQQEDTFICERVQKGLRSSAYDV........
XP_004333794|Acanthamoeba_castellanii_str._Neff     .........TKKEMVKALPESLEASIRISDGIQDEDAWVSEMVQQGLNSRAYDV........
GAQ84770|Klebsormidium_flaccidum                    ................ISDETLEENWVRTDVLQDEDVFLCEAVQKGLASGMFES........
```

```
                                                                    420        430
                                                                     .          .
BAC77698|Atriplex_nummularia                               .............GRY..VMPIEKGIHHFHCWLHQVLK........................
AGC13074|Atriplex_semibaccata                              .............GRY..VMPIEKGIHHFHCWLHQVLK........................
AHH24260|Atriplex_amnicola                                 .............GRY..VMPIEKGIHHFHCWLHQVLK........................
AFG28558|Atriplex_canescens__four-winged_saltbush          .............GRY..VMPIEKGIHHFHCWLHQVLK........................
Q9LKN0|Atriplex_hortensis                                  .............GRY..VMPIEKGIHHFHCWLHQVLK........................
AAL92561|Atriplex_prostrata                                .............GRY..VMPIEKGIHHFHCWLHQVLK........................
O04121|Spinacia_oleracea__spinach_                         .............GRY..VMPIEKGIHHFHCWLQQTLK........................
ABG34274|Ophiopogon_japonicus                              .............GRY..VMPIEKGIHHFHCWLQQTLK........................
AEW31326|Haloxylon_persicum                                .............GRY..VMPIEKGIHHFHCWLHQILK........................
ACX47904|Haloxylon_ammodendron                             .............GRY..VMPIEKGIHHFHCWLHQILK........................
AJF98574|Salicornia_bigelovii                              .............GRY..VMPIEKGIHHFHCWLHQILK........................
AAV91779|Salicornia_europaea                               .............GRY..VMPIEKGIHHFHCWLHQILQ........................
AFW04225|Suaeda_maritima                                   .............GRY..VMPIEKGIHHFHCWLHQILK........................
AAM43920|Suaeda_liaotungensis                              .............GRY..VMPIEKGIHHFHCWLHQILK........................
XP_010682183|Beta_vulgaris_subsp._vulgaris__sugar          .............GRY..VMPIEKGIHHFHCWLHETLQ........................
AHYPO_002206-RA|Amaranthus_hypochondriacus_439             .............GRY..VMPIEKGIHHFHCWLHQTL.N.......................
BAF93187|Amaranthus_tricolor                               .............GRY..VMPIEKGIHHFHCWLHQTL.N.......................
XP_012828802|Erythranthe_guttata__spotted_monkey_          .............GRY..SPNVEMAMHHFHCLLHRNLTI.......................
Migut.J00779.1|Mimulus_guttatus                            .............GRY..SPNVEMAMHHFHCLLHRNLTI.......................
EYU18199|Erythranthe_guttata__spotted_monkey_flow          .............GRY..CPNVEMAVHHFHCQLHRNLSR.......................
KQ992415.1|Dorcoceras_hygrometricum_corrected              .............GRY..SPTVEMAMHHFHCLLQENLRN.......................
XP_011094129|Sesamum_indicum__sesame_                      .............GRY..SPTVEMAMHHFHCLLQENLSQSFRRSR.................
XP_015080536|Solanum_pennellii__Lycopersicon_penn          .............GRY..APQVEKAMHHFHSLLYENLSD.......................
XP_004242785|Solanum_lycopersicum__Lycopersicon_e          .............GRY..APQVEKAMHHFHSLLYENLSD.......................
NP_001275051|Solanum_tuberosum__potato_                    .............GRY..APQVEKAMHHFHSLVYQNLSD.......................
XP_016556147|Capsicum_annuum                               .............GRY..APQVEKAMHHFHCLLYENLSC.......................
ACR15118|Lycium_barbarum                                   .............GRY..APQVEKAMHHFHSLLYENLHN.......................
XP_009757252|Nicotiana_sylvestris__wood_tobacco_           .............GRY..APQVEKAMHHFHCLLYENLCN.......................
XP_016460838|Nicotiana_tabacum_(common_tobacco)            .............GRY..APQVEKAMHHFHCLLYENLCN.......................
OIT26339|Nicotiana_attenuata                               .............GRY..APQVEKAMHHFHCLLYENLCN.......................
XP_009601434|Nicotiana_tomentosiformis                     .............GRY..APQVEKAMHHFHCLLYENLCN.......................
XP_016508540|Nicotiana_tabacum_(common_tobacco)            .............GRY..APQVEKAMHHFHCLLYENLCN.......................
XP_015081828|Solanum_pennellii__Lycopersicon_penn          .............GRY..VPQVEKAVHHFHSLLYESLSN.......................
XP_004243034|Solanum_lycopersicum__Lycopersicon_e          .............GRY..VPQVEKAVHHFHSLLYESLSN.......................
CDP08949|Coffea_canephora                                  .............GRY..APTVEKPMHHFHRLLYENLCN.......................
XP_019182381|Ipomoea_nil__Japanese_morning_glory_          .............GRY..APSVEKPMHHFHCLLHQNLCL.......................
XP_010433050|Camelina_sativa__false_flax_                  .............GRY...ALVEKPMHHFHCLLHQNLKL.......................
XP_010436340|Camelina_sativa_false_flax_corr               .............GRY...ALVEKPMHHFHCLLHQNLKL.......................
XP_010438247|Camelina_sativa__false_flax_                  .............GRY...TLVEKPMHHFHCLLHQNLKL.......................
XP_010447794|Camelina_sativa__false_flax_                  .............GRY...ALVEKPMHHFHCLLHQNLKL.......................
XP_006285614|Capsella_rubella                              .............GRY...ALVEKPMHHFHCLLHQNLKL.......................
Cagra.0268s0015.1|Capsella_grandiflora                     .............GRY...ALVEKPMHHFHCLLHQNLKL.......................
Bostr.7867s0836.1|Boechera_stricta                         .............GRY...ALVEKPMHQFHCWLHQNLKL.......................
NP_194718|Arabidopsis_thaliana__thale_cress_               .............GRY...ALVEKPMHHFHCLLHHNLKL.......................
XP_002869406|Arabidopsis_lyrata_subsp._lyrata              .............GRY...ALVEKPMHHFHCLLHHNLKL.......................
Araha.6052s0002.1|Arabidopsis_halleri                      .............GRY...ALVEEPMHHFHCLLHHNLKL.......................
XP_006412796|Eutrema_salsugineum                           .............GRY...ALVEKPMHHFHCLLHQNLKAMKQ....................
KFK29548|Arabis_alpina__gray_rockcress_                    .............GRY...ALVEKAMHHFHCLLHQNLKL.......................
KFK40841|Arabis_alpina__gray_rockcress_                    ..............................................................
XP_018481725|Raphanus_sativus__radish_                     .............GRY...ALVEKAMHHFHCLLHQNLKL.......................
XP_018435688|Raphanus_sativus__radish_                     .............GRY...ALVEKAMHHFHCLLHQNLKL.......................
XP_013705395|Brassica_napus__rape_                         .............GRY...ALAEKAMHHFHCLLHRNLKI.......................
XP_013596413|Brassica_oleracea_var._oleracea               .............GRY...ALVEKAMHHFHCLLHRNLKI.......................
XP_009137872|Brassica_rapa__field_mustard_                 .............GRY...ALVEKAMHHFHCLLHQNLKI.......................
XP_013738248|Brassica_napus__rape_                         .............GRY...ALVEKPMHHFHCLLHQNLKI.......................
XP_010541096|Tarenaya_hassleriana                          .............GRY..AP.VEEAMHHFHCLLHHNLSHS......................
Kaladp0809s0111.1|Kalanchoe_fedtschenkoi                   .............GRY..APTVEKPMHHFHCLLYHDLLRS......................
Kalax.0333s0026.1|Kalanchoe_laxiflora_corr_462aa           .............GRY..APTVEKPMHHFHCLLYHDLHRS......................
Kalax.0012s0114.1|Kalanchoe_laxiflora_corr_462aa           .............GRY..APTVEKPMHHFHCLLYHDLLRS......................
XP_010667950|Beta_vulgaris_subsp._vulgaris__sugar          .............GRY..APALEKAMHHFHCLLYRNLTG.......................
AH021158-RA|Amaranthus_hypochondriacus_421aa-corr          .............GRY..APTLEKPMHQFHCLLHSSLIERPILA..................
XP_021866412|426|Spinacia_oleracea_(spinach)               .............GRY..APTLEKPMHHFHCLLYRNLTEQTLQF..................
BAG74777|Hordeum_vulgare_subsp._vulgare__domestic          .............GRY..APSVEMAMHHFHCLLHANLSGQ......................
AKZ66518|Triticum_monococcum_subsp._aegilopoides           ..............................................................
FAOM01578495|Triticum_aestivum_UpdateAMA02065              .............GRY..APSVEMAMHHFHCLLHANLSG.......................
EMT07994|Aegilops_tauschii_                                .............GRY..APSVEMAMHHFHCLLHANLSG.......................
ACZ92178|Triticum_turgidum_subsp._durum__durum_wh          .............GRY..APSVEMAMHHF.................................
KD266899|Triticum_urartu_UpdateEMS46986                    .............GRY..APSVEMAMHHFHCLLHANLSG.......................
ABV64740|Leymus_chinensis                                  .............GRY..APSVEMAMHHFHCLLHANLSG.......................
Brast07G193900.1|Brachypodium_stacei                       .............GRY..TPSVEMAMHHFHRLLHTNLSG.......................
XP_003563491|Brachypodium_distachyon__stiff_brome          .............GRY..APSVEMAMHHFHRLLHTNLCG.......................
XP_006657294|Oryza_brachyantha__malo_sina_                 .............GRY..APSVEMAMHHFHCLLHANLSGDDW....................
XP_015643170|Oryza_sativa_Japonica_Group__Japanes          .............GRY..APSVEMAMHHFHCLLHANLSG.DW....................
EEC81252|Oryza_sativa_Indica_Group__long-grained_          .............GRY..APSVEMAMHHFHCLLHANLSG.DW....................
XP_002437525|Sorghum_bicolor__sorghum_                     .............GRY..APSVEMAMHHFHCLIHGNLSG.......................
NP_001105926|Zea_mays                                      .............GRY..APSVEMAMHHFHRLMHANLSEY......................
Pavir.Db00260.1|Panicum_virgatum_CORR_316f                 .............GRY..APSVEMAMHHFHCLLHSNLSS.......................
LWDX02005496.1|Dichanthelium_oligosanthes_update_          .............GRY..APSVEMAMHHFHCLLHANLSG.......................
Sevir.4G257300.1|Setaria_viridis                           .............GRY..APSVEMAMHHFHCLLHANLSG.......................
XP_004966128|Setaria_italica__foxtail_millet_              .............GRY..APSVEMAMHHFHCLLHANLSG.......................
Oropetium_20150105_16264A|Oropetium_thomaeum               .............GRY..APSVEMAMHHFHCLLHADLSG.......................
Pahal.D00004.1|Panicum_hallii                              .............GRY..APSVEMAMHHFHCLLHSNLSS.......................
Pavir.J39646.1|Panicum_virgatum_Corr_363f                  ..............................................................
OAY65202|Ananas_comosus__pineapple_                        .............GRY..APSVEMAMHHFHSLLHASLVDT......................
XP_009410214|Musa_acuminata_subsp._malaccensis__w          .............GRY..APTVEMAMHHFHCRLHECLGNI......................
XP_010927268|Elaeis_guineensis__African_oil_palm_          .............GRY..APSVEMAMHHFHCLLHACLDDL......................
XP_008801514|Phoenix_dactylifera__date_palm_               .............GRY..APSVEMAMHHFHCLLHACLNDL......................
KMZ72255|Zostera_marina                                    .............GRY..SPSLEKAMHHFHCLLHDKLIGI......................
JAT51600|Anthurium_amnicola                                .............GRY..APNVEQAMHHFHCLLYKSLNKM......................
Spipo5G0003400|Spirodela_polyrhiza                         .............GRY..APGVEQAMHHFHCLLRESLSN.......................
XP_006845775|Amborella_trichopoda                          .............GRY..APTVEHAMRHFHCCLHQNLI........................
AFP19450|Camellia_sinensis                                 .............GRY..APSVEKAMHHFHCLLYHNLIN.......................
XP_017254907|Daucus_carota_subsp._sativus                  .............GRY..APAIEKAMHHFHCLLHKNLVNE......................
ABX57826|Chrysanthemum_lavandulifolium                     ..............................................................
LEKV01000036.1|Cynara_cardunculus_update_KVI11819          .............GRY..APMVEKAMHHFHSLLHQHLIK.......................
XP_008443631|Cucumis_melo__muskmelon_                      .............GRY..APSVENAMHHFHRLLHRNLTK.......................
XP_004139149|Cucumis_sativus__cucumber_                    .............GRY..APSVENAMHHFHRLLHCNLTK.......................
XP_008351406|Malus_domestica__apple_                       ..............................................................
XP_008391562|Malus_domestica__apple_                       .............GRY..APTVENAMHHFHCLLHRSLTE.......................
XP_009343851|Pyrus_x_bretschneideri__Chinese_whit          .............GRY..APTVENAMHHFHCLLHKGLTE.......................
AER10510|Pyrus_betulifolia_corr_405aa                      .............GRY..APTVENAMHHFHCLLHKGLTE.......................
XP_008223853|Prunus_mume__Japanese_apricot_                .............GRY..APTVENAMHHFHCLLHKSLKK.......................
ONI27175|Prunus_persica__peach_                            .............GRY..APTVENAMHHFHCLLHKSLKK.......................
```

```
XP_004301419|Fragaria_vesca_subsp._vesca            ............GRY..APTVENAMYHFHRLLHESLRE.......................
XP_015889685|Ziziphus_jujuba__common_jujube_        ............GRY..APAVENAMHHFHCLLYDSLTK.......................
NW_010360102.1|Morus_notabilis_corrected            ............GRY..APKVENAMHHFHCLLHSSLNE.......................
Lus10032689|Linum_usitatissimum                     ............GRY..APTVENAMYHFHQLLHSDLS........................
Lus10008571|Linum_usitatissimum_corrected           ............GRY..APTVENAMYHFHQLLHSDLS........................
XP_011020077|Populus_euphratica__Euphrates_poplar   ............GRY..APTVENAMHHFHQLLHHILKE.......................
XP_002308100|Populus_trichocarpa__Populus_balsami   ............GRY..APMVEHAMHHFHQLLHYILKE.......................
SapurV1A.0198s0060.1|Salix_purpurea                 ............GRY..APTVENAMHHFHQLLHDILKE.......................
OAY46667|Manihot_esculenta__cassava_                ............GRY..APTVEQAMHHFHLLLHHHLKM.......................
XP_002518256|Ricinus_communis__castor_bean_         ............GRY..APTVEKAMHHFHQLLHGKLKI.......................
XP_012074438|Jatropha_curcas                        ............GRY..APTVEKAMHHFHQLLYDNLKI.......................
XP_010046909|Eucalyptus_grandis                     ............GRY..APTVEIAMYHFHGLLYRNLKG.......................
ABS71853|Eucalyptus_camaldulensis_Murray_red_gum_   ............GRY..APTVEIAMYHFHGLLYRNLKG.......................
XP_006474292|Citrus_sinensis__sweet_orange_         ............GSITGIIKEEEGMEFKVKGILT...........................
XP_006453220|Citrus_clementina_reviewed             ............GRY..APSVEQAMYHFHSLLHCNLTN.......................
XP_017612508|Gossypium_arboreum                     ............GRY..APNVEKAMHHFHCLLYDNLIN.......................
XP_016686114|Gossypium_hirsutum__cotton_            ............GRY..APNVEKAMHHFHCLLYDNLIN.......................
XP_012459381|Gossypium_raimondii                    ............GRY..APNVEKAMHHFHCLLYDNLINFFCLPNAFLNCCLAFIE......
XP_016719040|Gossypium_hirsutum__cotton_            ............GRY..APNVEKAMHHFHCLLYDNLIN.......................
EOY05290|Theobroma_cacao__cacao_                    ............GRY..APTVEKAMHHFHCLLYDNLIN.......................
AWWV01015497-AWWV01004954_Corchorus_capsularis_39   ............GRY..APTVEKAMHHFHCLLYDNLIK.......................
OMO51681|Corchorus_olitorius                        ............GRY..APTVEKAMHHFHCLLYDNLIN.......................
GAV59711|Cephalotus_follicularis                    ............GRY..APTIEKAMHHFHCLLHNNLLNSK.....................
XP_018807260|Juglans_regia__English_walnut_         ............GRF..APTVEKAMYHFHGLLHQNLTNDCLV...................
XP_019078540|Vitis_vinifera__wine_grape_            ............GRY..APTVEMAMHHFHCLLHENLTK.......................
Aqcoe7G261800.1|Aquilegia_coerulea                  ............GRY..APTVEKAMHHFHCLLSENLTK.......................
XP_010276721|Nelumbo_nucifera__sacred_lotus_        ............GRY..APTVEKAMHHFHRLLYENLTK.......................
XP_016201893|Arachis_ipaensis                       ............GRY..APKVEQAMHHFHCLLYENLKK.......................
XP_015973237|Arachis_duranensis                     ............GRY..APKVEQAMHHFHCLLYENLKK.......................
Tp57577_TGAC_v2_mRNA5577|Trifolium_pratense         ............GRY..APKVEQAMHHFHCLLYENLTT.......................
GAU38011|Trifolium_subterraneum                     ............GRY..APKVEQAMHHFHRLLYENLTT.......................
XP_003610028|Medicago_truncatula__barrel_medic_     ............GRY..APKVEQAMHHFHCLLYENLTT.......................
XP_004507911|Cicer_arietinum__chickpea_             ............GRY..APKVEQAMHHFHCLLHENLTT.......................
XP_019463425|Lupinus_angustifolius__narrow-leaved   ............GRY..APRVEQAMHHFHCLLYENLTK.......................
KYP52554|Cajanus_cajan__pigeon_pea_                 ............GRY..APTVEQAMHHFHCLLYEDLTK.......................
XP_003549280|Glycine_max__soybean_                  ............GRY..APTVEQAMHHFHCLLYENLAK.......................
XP_007134857|Phaseolus_vulgaris_CHR1_corr_208aaf    .............................................................
XP_007154695|Phaseolus_vulgaris_CORR                ............GRY..APTVEQAMHHFHCLLYENLTK.......................
XP_017410379|Vigna_angularis__adzuki_bean_          ............GRY..APTVELAMHHFHCLLYENLTK.......................
XP_014508581|Vigna_radiata_var._radiata__mung_bea   ............GRY..APTVELAMHHFHCLLYENLTK.......................
AUSU01000986.1|Genlisea_aurea_corrected-f           ............GRY..SPSVETAMHHFHRLLHKNLSD.......................
OAE24413|CORR_Marchantia_polymorpha                 ............GRY..APKVEYAMHHFHVLLHEDLSM.......................
Sphfalx0064s0059.1|Sphagnum                         ............GRY..APRVEHAMHHFHSLLHNDLSS.......................
XP_001752587|Physcomitrella_patens                  ............GRY..APRVEQAMHHFHCLIYDDLSAGMIS...................
XP_002508933|Micromonas_sp._RCC299                  ............GRY..APAMEAAMYHFHRRLWRDLTAE......................
XP_003061830|Micromonas_pusilla_CCMP1545            ............GRY..APAVEAAMYHFHALL.............................
XP_005645368|Coccomyxa                              ............GRY..APGLEQPMFHFHKLLQRDLMGSAQLEA.................
ADIC01002027.1|Chlorella_variabilis_corrected_pre   ............GRY..APGVEGPMFHFHQLLHADLAAEC.....................
XP_013903967|Monoraphidium_neglectum_rev            ............GRY..APSLEQPMFHFHQLLHQAIVGAGDSNVDSSSSRFSFSSKKV...
XP_005644270|Coccomyxa_subellipsoidea_C-169         ............GRY..APSVEEPVFHFHKLVHRDLVGQ......................
NC_024001.1|Bathycoccus_prasinos_corrected          RSGSGGDEDRQEGLY..SPECESIMFAFHKRYYRAVLLPGE....................
EWM23896|Nannochloropsis_gaditana                   ............GRY..APRVEFLDHVFHQWLAQDLRAAVGKIQGREGTHRPRWGKRGRML
XP_002738379|Saccoglossus_kowalevskii               ............GRY..APGVEFADHMFHVKLAELFRRHLKIP..................
XP_006820984|Saccoglossus_kowalevskii               ............GRY..APQDEKPDYAFHRHLAEDFKQYLSLHRN................
XP_002737044|Saccoglossus_kowalevskii               ............GRY..APRIEYPQHEFHVRLGKEFRAYLKNKS.................
XP_002597329|Branchiostoma_floridae__Florida_lanc   ............GRY..LPAIEHGPHRFHIFLGTELRKGLKL...................
XP_019618440|Branchiostoma_belcheri__Belcher's_la   ............GRY..LPMIEHGPHMFHVFLGTELRKGLELGQ.................
XP_019644608|Branchiostoma_belcheri__Belcher's_la   ............GRY..APGPEMPVHAFHLQLARDLREGLEHHESMYSNN...........
XP_019644788|Branchiostoma_belcheri__Belcher's_la   ............GRY..APGPEMPVHAFHLQLARDLREGLEHHESMYSTN...........
XP_002610312|Branchiostoma_floridae__Florida_lanc   ............GRY..APGPEMPVHAFHLQLARDLRAGLESMDISC..............
XP_002599795|Branchiostoma_floridae__Florida_lanc   ............GRL..MF..........................................
XP_019633636|Branchiostoma_belcheri__Belcher's_la   ............GRY..APRVEMADHAFHLKLASDLKQGIRTDKLTNGITVPCMF......
XP_004333794|Acanthamoeba_castellanii_str._Neff     ............GRY..APQVETAAYAFHQLLASDFRHGLQPSQ.................
GAQ84770|Klebsormidium_flaccidum                    ............GRY..VAPQEEAPHHFHRLIHSDFVAYEKDL..................
```

Fig S1: Complete alignment of CMO protein sequences
