# Supplementary figures and images for: Bona fide choline monoxygenases evolved in Amaranthaceae plants from oxygenases of unknown function: Evidence from phylogenetics, homology modeling and docking studies
Source: PLoS One. 2018 Sep 26;13(9):e0204711. doi: 10.1371/journal.pone.0204711 (PMC6157903; doi:10.1371/journal.pone.0204711)

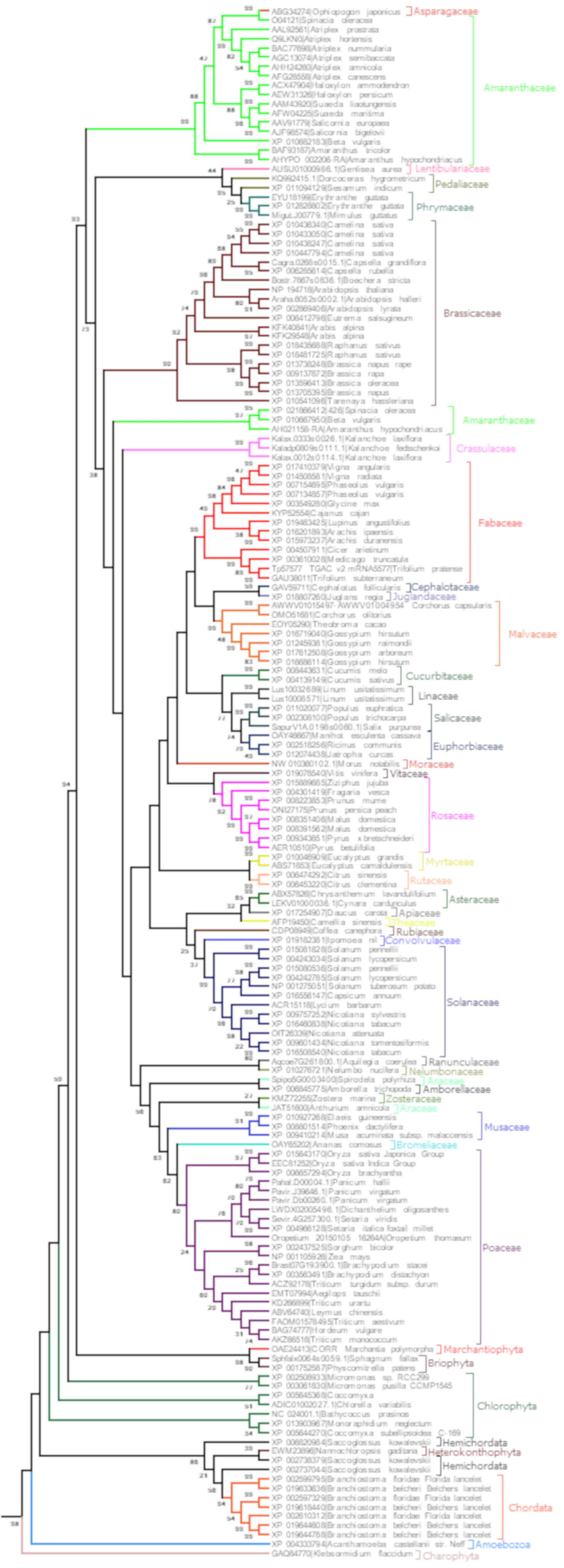

Supplement: S2 Fig — (TIFF) [file pone.0204711.s002.tiff]
