# Supplementary material for: Bona fide choline monoxygenases evolved in Amaranthaceae plants from oxygenases of unknown function: Evidence from phylogenetics, homology modeling and docking studies
Source: PLoS One. 2018 Sep 26;13(9):e0204711. doi: 10.1371/journal.pone.0204711 (PMC6157903; doi:10.1371/journal.pone.0204711)
Supplement: S1 Table — (PDF) [file pone.0204711.s003.pdf]

Supplementary Material

Bona fide choline monoxygenases evolved in Amaranthaceae plants from oxygenases of unknown function: evidence from phylogenetics, homology modeling and docking studies

Javier Carrillo-Campos[1], Héctor Riveros-Rosas[2], Rogelio Rodríguez-Sotres[1], and Rosario A. Muñoz-Clares[1*]

[1]Departamento de Bioquímica, Facultad de Química, Universidad Nacional Autónoma de México, Ciudad de México, 04510. México.

[2]Departamento de Bioquímica, Facultad de Medicina, Universidad Nacional Autónoma de México, Ciudad de México, Ciudad de México, 04510. México.

***Correspondence:** Rosario A. Muñoz-Clares, Departamento de Bioquímica, Facultad de Química, Universidad Nacional Autónoma de México, Ciudad de México, 04510. México.

***E-mail:** clares@unam.mx

Tel: +52 5556223718

Short title: Evolution of choline monooxygenase proteins

Supplementary Table S1. Proteins identified as members of the CMO subfamily

Amino acid sequences were retrieved from NCBI or from Phytozome 12.1.5. * Sequences corrected by us; in these cases, the number of residues given in the table corresponds to the corrected sequence. ** The two domains of this protein are reported as separate sequences in the NCBI database, each with different accession number.

SPECIES	ACCESSION NUMBER		LENGTH
	NCBI	PHYTOZOME	
**HETEROKONTHOPHYTA**			
Nannochloropsis gaditana	EWM23896	—	511 aa
**CHLOROPHYTA**			
Bathycoccus prasinos	XP_007511423	—	452 aa*
Coccomyxa subellipsoidea C-169	XP_005644270	—	368 aa
	XP_005645368	—	357 aa
Chlorella variabilis	XP_005845791	—	434 aa*
Micromonas pusilla CCMP1545	XP_003061830	—	338 aa
Micromonas sp. RCC299	XP_002508933	—	364 aa
Monoraphidium neglectum	XP_013903967	—	448 aa
**CHAROPHYTA**			
Klebsormidium flaccidum	GAQ84770	—	481 aa
**BRIOPHYTA**			
Physcomitrella patens subsp. *patens*	XP_001752587	Pp3c13_13580	414 aa
Sphagnum fallax	—	Sphfalx0064s0059	430 aa
**MARCHANTIOPHYTA**			
Marchantia polymorpha	OAE24413	Mapoly0089s0013	441 aa*
**LYCOPODIOPHYTA**			
Selaginella moellendorffii	XP_002981049	SELMODRAFT_114039	410 aa
**MONOCOTYLEDONS**			
Aegilops tauschii	EMT07994	—	342 aa
Amborella trichopoda	XP_006845775	evm_27.model.AmTr_v1.0_scaffold00019.418	422 aa
Ananas comosus	OAY65202	Aco012177LG02	420 aa
Anthurium amnicola	JAT51600	—	380 aa
Brachypodium distachyon	XP_003563491	Bradi1g33960	409 aa
Brachypodium stacei	—	Brast07G193900	408 aa
Dichanthelium oligosanthes	OEL37452	—	414 aa*
Elaeis guineensis	XP_010927268	—	426 aa
Hordeum vulgare subsp. *vulgare*	BAG74777	—	407 aa
Leymus chinensis	ABV64740	—	342 aa
Musa acuminata	XP_009410214	GSMUA_Achr7T19580_0	425 aa
Ophiopogon japonicus	ABG34274		438 aa
Oropetium thomaeum	—	Oropetium_20150105_16264	308 aa
Oryza brachyantha	XP_006657294	—	362 aa
Oryza sativa Japonica Group	XP_015643170	—	410 aa
Oryza sativa Indica Group	EEC81252	LOC_Os06g48510	410 aa

SPECIES	ACCESSION NUMBER		LENGTH
	NCBI	PHYTOZOME	
Panicum halli	—	Pahal.D00004	407 aa
Panicum virgatum	—	Pavir.J39646	363 aa*
	—	Pavir.Db00260	316 aa*
Phoenix dactylifera	XP_008801514	—	426 aa
Setaria italica	XP_004966128	Si006556m.g	411 aa
Setaria viridis	—	Sevir.4G257300	411 aa
Sorghum bicolor	XP_002437525	Sb10g028700	405 aa
Spirodela polyrrhiza	—	Spipo5G0003400	322 aa
Triticum aestivum	AMA02065	—	411 aa*
Triticum monococcum subsp. *aegilopoides*	AKZ66518	—	86 aa
Triticum urartu	EMS46986	—	365 aa*
Zea mays	NP_001105926	Zm00008a024486	408 aa
Zostera marina	KMZ72255	ZOSMA_169G00470	413 aa
**EUDICOTYLEDONS**			
Amaranthus hypochondriacus	—	AHYPO_002206-RA	439 aa
		AHYPO_004856-RA	421 aa
Amaranthus tricolor	BAF93187	—	442 aa
Aquilegia coerulea	—	Aqcoe7G261800	428 aa
Arabis alpina	KFK29548	—	420 aa
	KFK40841	—	236 aa
Arabidopsis halleri	—	Araha.6052s000	423 aa
Arabidopsis lyrata subsp. *lyrata*	XP_002869406	AL7G22360	422aa
Arabidopsis thaliana	NP_194718	At4g29890	422 aa
Arachis duranensis	XP_015973237	—	418 aa
Arachis ipaensis	XP_016201893	—	418 aa
Atriplex amnicola	AHH24260	—	438 aa
Atriplex canescens	AFG28558	—	438 aa
Atriplex hortensis	Q9LKN0	—	438 aa
Atriplex nummularia	BAC77698	—	438 aa
Atriplex prostrata	AAL9256	—	438 aa
Atriplex semibaccata	AGC13074	—	438 aa
Bassia scoparia	AAQ92313	—	290 aa
Beta vulgaris	XP_010682183	—	446 aa
	XP_010667950	—	418 aa
Boechera stricta	—	Bostr.7867s083	424 aa
Brassica napus	XP_013738248	—	440 aa
	XP_013705395	—	438 aa
Brassica oleraceae	XP_013596413	Bol033622	438 aa
Brassica rapa	XP_009137872	Brara.K00866	432 aa
Cajanus cajan	KYP52554	—	416 aa
Camellia sinensis	AFP19450	—	434 aa

SPECIES	ACCESSION NUMBER		LENGTH
	NCBI	PHYTOZOME	
Camelina sativa	XP_010433050	—	427 aa
	XP_010436340	—	286 aa
	XP_010438247	—	427 aa
	XP_010447794	—	427 aa
Capsella rubella	XP_006285614	Carubv10007064m.g	423 aa
Capsella grandiflora	—	Cagra.0268s0015	423 aa
Capsicum annuum	XP_016556147	—	422 aa
Cephalotus follicularis	GAV59711	—	418 aa
Chrysanthemum lavandulifolium	ABX57826	—	201 aa
Cicer arietinum	XP_004507911	—	414 aa
Citrus clementina	XP_006453220	Ciclev10008481m.p	408 aa
Citrus sinensis	XP_006474292	—	413 aa
Coffea canephora	CDP08949	—	431 aa
Corchorus capsularis	OMP06320-OMO52515**	—	395 aa
Corchorus olitorius	OMO51681	—	364 aa
Cynara cardunculus	KVI11819	—	411 aa*
Cucumis melo	XP_008443631	—	417 aa
Cucumis sativus	XP_004139149	Cucsa.356080	417 aa
Daucus carota	XP_017254907	DCAR_020143	415 aa
Dorcoceras hygrometricum	KZV50441	—	416 aa*
Eucalyptus camaldulensis	ABS71853	—	429 aa*
Eucalyptus grandis	XP_010046909	EUGRSUZ_C00087	429 aa
Eutrema salsugineum	XP_006412796	Thhalv10025251m	432 aa
Fragaria vesca subsp. *vesca*	XP_004301419	mrna12324.1-v1.0	410 aa
Glycine max	XP_003549280	Glyma.17G057700	418 aa
Gossypium arboreum	XP_017612508	—	444 aa
Gossypium hirsutum	XP_016719040	—	381 aa
	XP_016686114	—	409 aa
Gossypium raimondii	XP_012459381	Gorai.012G008200	428 aa
Haloxylon ammodendron	ACX47904	—	448 aa
Haloxylon persicum	AEW31326	—	447 aa
Ipomoea nil	XP_019182381	—	418 aa
Jatropha curcas	XP_012074438	—	414 aa
Juglans regia	XP_018807260	—	430 aa
Kalanchoe fedtschenkoi	—	Kaladp0809s0111	462 aa
Kalanchoe laxiflora	—	Kalax.0333s0026	462 aa
		Kalax.0012s0114	462 aa
Linum usitatissimum	—	Lus10032689	427 aa
		Lus10008571	427 aa
Lycium barbarum	ACR15118	—	427 aa

SPECIES	ACCESSION NUMBER		LENGTH
	NCBI	PHYTOZOME	
Lupinus angustifolius	XP_019463425	—	411 aa
Malus domestica	XP_008391562	MDP000022353	405 aa
	XP_008351406	MDP000031939	359 aa
Manihot esculenta	OAY46667	Manes.06G017600	417 aa
Medicago truncatula	XP_003610028	MTR_4g127130	411 aa
Mimulus guttatus	XP_012828802	MIMGU_mgv1a006513mg	433 aa
	EYU18199	MIMGU_mgv1a006978mg	424 aa
Morus notabilis	XP_010094241	—	407 aa*
Nelumbo nucifera	XP_010276721	—	420 aa
Nicotiana attenuata	OIT26339	—	480 aa
Nicotiana sylvestris	XP_009757252	—	427 aa
Nicotiana tabacum	XP_016508540	—	436 aa
	XP_016460838	—	427 aa
Nicotiana tomentosiformis	XP_009601434	—	436 aa
Phaseolus vulgaris	XP_007154695	Phvul.003G140100	416 aa
	XP_00713286	Phvul.011G131100	200 aa
	XP_00713485	Phvul.010G082000	188 aa
Populus euphratica	XP_011020077	—	424 aa
Populus trichocarpa	XP_002308100	POPTR_0006s07230	424 aa
Prunus persica	ONI27175	Prupe.1G072500	414 aa
Prunus mume	XP_008223853	—	414 aa
Pyrus betulifolia	AER10510	—	405 aa
Pyrus bretschneideri	XP_009343851	—	405 aa
Raphanus sativus	XP_018435688	—	457 aa
	XP_018481725	—	456 aa
Ricinus communis	XP_002518256	30198.t000028	417 aa
Salicornia bigelovii	AJF98574	—	439 aa
Salicornia europaea	AAV91779	—	442 aa
Salix purpurea	—	SapurV1A.0198s0060	424 aa
Sesamum indicum	XP_011094129	—	435 aa
Solanum lycopersicum	XP_004242785	Solyc07g008310	412 aa
	XP_004243034	Solyc07g009300	410 aa
Solanum penelli	XP_015080536	—	412 aa
	XP_015081828	—	410 aa
Solanum tuberosum	NP_001275051	PGSC0003DMT400051201	412 aa
Spinacia oleracea	XP_021849509	—	439 aa
	XP_021866412	—	426 aa
Suaeda liaotungensis	AAM43920	—	442 aa
Suaeda maritima	AFW04225	—	442 aa
Suaeda salsa	ABG43004	—	442 aa
Tarenaya hassleriana	XP_010541096	—	438 aa

SPECIES	ACCESSION NUMBER		LENGTH
	NCBI	PHYTOZOME	
Theobroma cacao	EOY05290	Thecc1EG020326t1	407 aa
Trifolium pratense	—	Tp57577_TGAC_v2_mRNA5577	432 aa
Trifolium subterraneum	GAU38011	—	390 aa
Vigna angularis	XP_017410379	—	416 aa
Vigna radiata	XP_014508581	—	416 aa
Vitis vinifera	XP_019078540	GSVIVT01029282001	441 aa
Ziziphus jujuba	XP_015889685	—	404 aa
**CHORDATA**			
Branchiostoma belcheri	XP_019633636	—	418 aa
	XP_019618440	—	409 aa
	XP_019644608	—	449 aa
	XP_019644788	—	442 aa
Branchiostoma floridae	XP_002599795		381 aa
	XP_002597329		410 aa
	XP_002610312		357 aa
**HEMICHORDATA**			
Saccoglossus kowalevskii	XP_002738379		400 aa
	XP_006820984		403 aa
	XP_002737044		416 aa
**AMOEBOZOA**			
Acanthamoeba castellanii	XP_004333794	—	458 aa
